# Influence of Ligand Isomerism on the Photophysical Properties of AIPE-Active Rhenium(I) Complexes: Investigations with a 2-(1,2,3-Triazol-1-yl)pyridine (Tapy)-Based Complex and Its Triazolylidene Derivatives

**DOI:** 10.3390/molecules30132776

**Published:** 2025-06-27

**Authors:** Abanoub Mosaad Abdallah, Mariusz Wolff, Nadine Leygue, Maëlle Deleuzière, Nathalie Saffon-Merceron, Charles-Louis Serpentini, Eric Benoist, Suzanne Fery-Forgues

**Affiliations:** 1 Laboratoire Synthèse et Physicochimie des Molécules d’Intérêt Biologique (SPCMIB), CNRS UMR 5068, Université de Toulouse, 118 Route de Narbonne, 31062 Toulouse CEDEX 9, France; aabdelmonaem90@gmail.com (A.M.A.); nadine.leygue@univ-tlse3.fr (N.L.); maelle.deleuziere@univ-tlse3.fr (M.D.); 2 Narcotic Research Department, National Center for Social and Criminological Research (NCSCR), Giza 11561, Egypt; 3 Institut für Funktionelle Materialien und Katalyse, Universität Wien, Währinger Straße 38-42, 1090 Wien, Austria; mariusz.jozef.wolff@univie.ac.at; 4 Institute of Chemistry, University of Silesia in Katowice, Szkolna 9th Street, 40-006 Katowice, Poland; 5 Service Diffraction des Rayons X, Institut de Chimie de Toulouse, ICT-UAR 2599, Université de Toulouse, 118 Route de Narbonne, 31062 Toulouse CEDEX 9, France; nathalie.saffon@utoulouse.fr; 6 Laboratoire de Chimie des Colloïdes, Polymères & Assemblages Complexes (SOFTMAT), CNRS UMR 5623, Université de Toulouse, 118 Route de Narbonne, 31062 Toulouse CEDEX 9, France; charles-louis.serpentini@univ-tlse3.fr

**Keywords:** rhenium complexes, crystallography, DFT calculations, photoluminescence

## Abstract

Due to their rare properties of solid-state luminescence enhancement (SLE), tricarbonylrhenium complexes are promising candidates for applications as photoluminescent materials. However, the effect of isomerism on optical properties is still not well known. The aim of this in-depth study is to explore the behavior of a 2-(1,2,3-triazol-1-yl)pyridine (tapy) complex and compare it with that of the isomers studied previously. Two derivatives that incorporate a mesoionic carbene ligand and represent an emerging class of molecules were also synthesized and compared with the corresponding isomers. The crystallographic data revealed that compounds in the solid state have little or no π–π interactions. The spectroscopic study was supported by DFT calculations. All the compounds were weakly phosphorescent in solution but exhibited a marked SLE effect. The Re-Tapy complex is an excellent solid-state emitter (PLQY = 0.62), well suited for applications related to aggregation-induced phosphorescence emission (AIPE). Its sensitivity to mechanical stimuli was unprecedented among the isomers considered to date. On the other hand, triazolylidene complexes are less emissive than their pyta_(1,2,3)_ counterparts. This study shows how the ligand isomerism influences the optical properties of tricarbonylrhenium(I) complexes. It indicates that selecting the right pattern is a key factor for the design of efficient photoluminescent materials.

## 1. Introduction

A number of tricarbonylrhenium(I) complexes emit light more efficiently in the solid/aggregate state than in solution. They occupy a special position among the few transition metal complexes that exhibit solid-state luminescence enhancement (SLE) [1], or more precisely, aggregation-induced phosphorescence emission (AIPE) properties [2,3,4,5,6]. These air and water stable complexes are phosphorescent at room temperature, their long emission lifetimes and large Stokes shifts allow easy detection, and their emission efficiency can be finely tuned by varying the nature of their ligands. Many of them are photochemically stable and biocompatible, allowing for prolonged use even in biological environments. These characteristics make them promising candidates for various solid-state applications, including light-emitting devices [7,8,9,10,11,12,13,14], and sensors for the detection of biomolecules [15,16,17,18,19] and explosives [20,21,22]. In addition, our group showed that some of these complexes exhibit mechanoresponsive luminescence (MRL) [23,24,25,26,27], i.e., a particular sensitivity to mechanical stimuli, which makes them potential materials for pressure sensing, anti-counterfeiting and optical data recording [28,29]. However, the development of these complexes is hampered by the fact that solid-state photoluminescence (PL) properties depend on very small changes in molecular geometry and intermolecular arrangement, which are difficult to predict.

**Figure 1:**
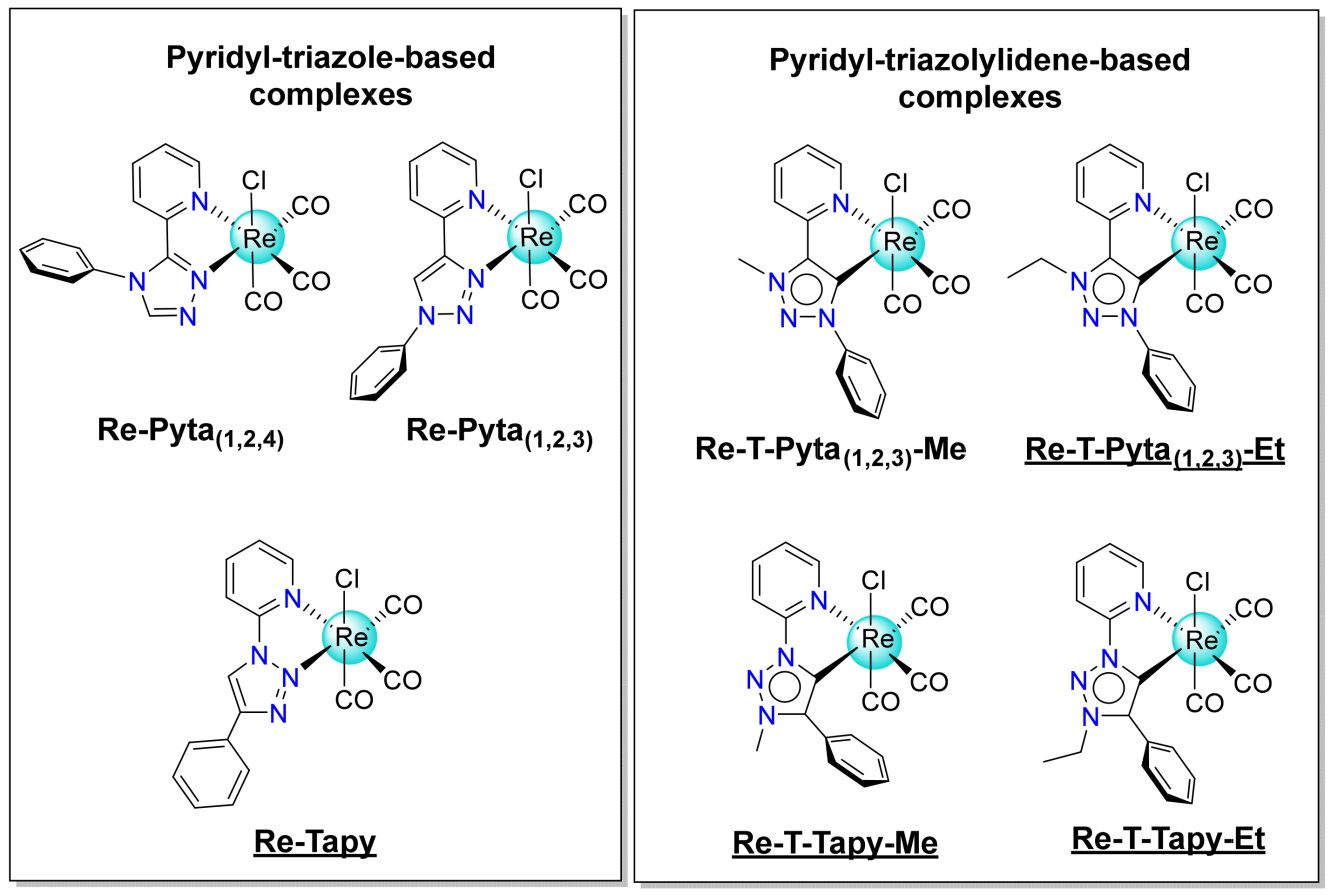
Chemical structures of the pyridyl-triazole-based complexes (**Re-Pyta_(1,2,4)_ [24]**, **Re-Pyta_(1,2,3)_ [32]**, and **Re-Tapy**) and pyridyl-triazolylidene-based complexes (**Re-T-Pyta_(1,2,3)_-Me** [32], **Re-T-Pyta_(1,2,3)_-Et**, **Re-T-Tapy-Me**, and **Re-T-Tapy-Et**). The new complexes synthesized for this study are underlined.

To date, the vast majority of tricarbonylrhenium(I) complexes studied for their solid state properties contain a diimine ligand, which sometimes exists in the form of several isomers. The comparison of isomers can provide valuable information on how minor structural differences influence PL properties, but such studies are still rare. The Machura team has shown that PL properties are sensitive to the position of a nitrogen atom in a pyridine ring borne by a 2,2′:6′,2″-terpyridine (terpy) ligand [9] and a 2,6-di(thiazol-2-yl)pyridine (dtpy) ligand [11]. The substitution pattern of a naphthyl or methoxynaphthyl moiety on terpy, dtpy, and 2,6-di(pyrazin-2-yl)pyridine (dppy) frameworks also plays a role in the emission of the corresponding complexes as solids and/or frozen solutions [10,30]. In recent years, our team has been interested in complexes that feature a bidentate pyridyl-triazole (pyta) moiety, which has excellent chelating ability and adaptability to chemical modifications. We showed that complexes containing a 1,2,4-triazole fragment (e.g., **Re-Pyta_(1,2,4)_**, Figure 1) do not behave like their 1,2,3-triazole analogs (e.g., **Re-Pyta_(1,2,3)_**) [31,32]. In the pyta_(1,2,4)_ series, the position of a methyl substituent [26] and the way to insert a benzoxazole heterocycle [33] completely alter the PL properties. Major differences were also reported for isomeric dinuclear Re(I) complexes, whose PL efficiency and MRL properties depend on whether the coordination spheres are inserted in the *para* or *meta* position of a central phenyl ring [27]. In closely related fields, it has been shown that a rhodium(III) complex incorporating a 4-(3,5-dimethyl-1H-pyrazol-1-yl)-2,5-diphenylpyrimidine ligand emits light in the solid state, while the 2,6-diphenylpyrimidine isomer is not emissive at all [34]. In another study, the phosphorescent iridium(III) complex with a 2-(1,3-diphenyl-1H-1,2,4-triazol-5-yl)pyridine ligand has MRL properties, which is not the case for the isomer containing a 2-(3,5-diphenyl-1H-1,2,4-triazol-1-yl)pyridine [35].

The PL properties of Re(I) complexes incorporating a 1,2,3-triazol-5-ylidene ligand have also been very little investigated. These mesoionic carbene ligands are modularly synthesized by Cu(I)-catalyzed “click” reactions and offer a broad selection of substituents. Their remarkable sigma electron donation capabilities make them excellent ligands for a variety of transition metals, resulting in complexes with very attractive physicochemical properties [36,37,38]. However, there are few examples of rhenium(I) complexes constructed with these ligands [39,40]. To the best of our knowledge, our team was the first to report their value as solid-state emitters [32], and isomerism has not yet been taken into account.

In the continuation of our study on the effect of isomerism, we focused our attention on a new complex that incorporates a 2-(1,2,3-triazol-1-yl)pyridine (tapy) ligand. The potential of the tapy ligand was first demonstrated by Bertrand et al., who compared the properties of alkyl-substituted pyta and tapy complexes in solution for bio-imaging [41,42]. In parallel, Crowley and co-workers studied aryl-substituted tapy complexes, and showed that in solution, the photophysical properties are very close to those of 1,2,3-pyta analogs, with the exception of a red shift in absorption and emission spectra [43]. Solid-state properties have hardly been studied. Only a recent work by Neumann et al. has reported for long-chain Re(I) complexes that the lengthening of solid-state emission lifetimes relative to solutions is greater in the pyta series than in the tapy series [44]. Therefore, complex **Re-Tapy**, which is the exact isomer of our complexes **Re-Pyta_(1,2,4)_** and **Re-Pyta_(1,2,3)_**, was synthesized and studied in solid state. The aromatic ring was initially introduced to promote an intermolecular arrangement favorable to solid-state light emission, and allow subsequent substitutions to modulate the physicochemical properties. The chloride ancillary ligand endows the complexes with excellent photochemical stability. Then, the corresponding pyridyl-triazolylidene-based complex **Re-T-Tapy-Me** was prepared, as well as the derivative bearing an ethyl group instead of a methyl group on the triazolylidene ring (**Re-T-Tapy-Et**), to see if such a negligible structural modification can play a role. Solid-state molecular arrangements were deduced from crystallographic data and theoretical calculations were used to explain the spectroscopic behavior. Finally, the PL properties of these three new complexes were compared with those of the corresponding isomers, including the triazolylidene complex **Re-T-Pyta_(1,2,3)_-Et**, which bears an ethyl chain and was synthesized for the sake of comparison. This comprehensive analysis provides deeper insights into the design of rhenium(I) complexes with tailored emission properties, potentially guiding the development of new materials for advanced photonic applications.

## 2. Results and Discussion

### 2.1. Synthesis and Characterization

**Scheme 1:**
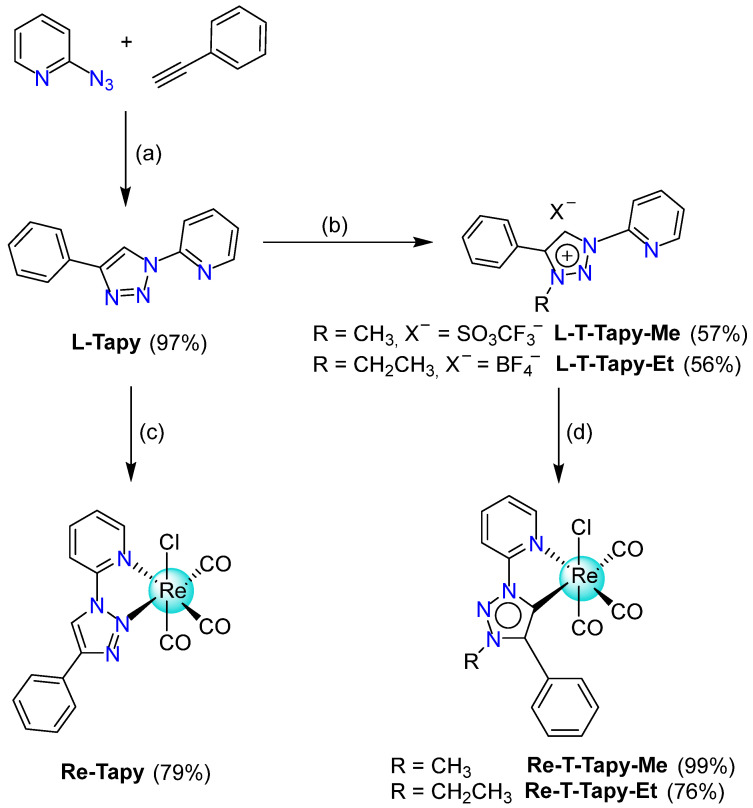
Synthesis of tricarbonylrhenium(I) complexes **Re-Tapy**, **Re-T-Tapy-Me**, and **Re-T-Tapy-Et**. Conditions: (a) Cu(I) acetate, Toluene, reflux, 24 h; (b) F_3_CSO_3_CH_3_ or Et_3_OBF_4_, DCM, RT, 1 day; (c) [Re(CO)_5_Cl], MeOH, reflux, 20 h; (d) [Re(CO)_5_Cl], NEt_3_, Toluene, reflux, 3 days.

Ligands **L-Pyta_(1,2,3)_** and **L-Tapy** were synthesized through Cu(I)-catalyzed cycloaddition reactions between the appropriate organic azide and alkyne according to established procedures of click chemistry [43,45]. They were obtained with a yield of 98% and 97%, respectively. The ethylated mesoionic carbene ligand **L-T-Pyta-Et** was prepared from **L-Pyta_(1,2,3)_** using a three-step procedure involving (*i*) protection of the pyridine ring by oxidation to *N*-oxide using *m*-chloroperoxybenzoic acid (*m*-CPBA), (*ii*) one-pot ethylation using Et_3_OBF_4_, and (*iii*) reductive cleavage of the N-O bond in pyridine *N*-oxide in the presence of hexacarbonyl molybdenum [Mo(CO)_6_] (Scheme S1) [46]. In contrast, the synthesis of the mesoionic carbene ligands **L-T-Tapy-Me** and **L-T-Tapy-Et** was carried out by direct alkylation using MeOTf and Et_3_OBF_4_, respectively (Scheme 1). This selectivity can be attributed to the reduced nucleophilicity of the pyridine lone pair due to the electronegative triazole nitrogen atom directly attached to the pyridine ring in the tapy ligand [46].

Then, **L-Tapy** was reacted with [Re(CO)_5_Cl] in refluxing methanol as described in ref. [43] to produce complex **Re-Tapy** with a 79% yield (Scheme 1). The triazol-5-ylidene ligands were reacted with [Re(CO)_5_Cl] with an excess of NEt_3_ in refluxing toluene for three days [39], resulting in the formation of **Re-T-Pyta_(1,2,3)_-Et** (Scheme S1), **Re-T-Tapy-Me**, and **Re-T-Tapy-Et** (Scheme 1) with yields of 98%, 99%, and 76%, respectively. It should be noted that using this synthesis strategy, adapted from optimized and well-established methods [39,43,45,46], ligands and their corresponding Re(I) complexes were prepared at the millimolar scale, with a satisfactory overall yield, without needing specialized equipment. Column chromatography was preferred as a purification method due to its high reproducibility and the high purity of the products obtained. More environmentally friendly alternatives, including a microwave-assisted synthesis and/or crystallization process and/or greener solvents will be explored in future work.

The structures of the newly synthesized compounds were elucidated by ^1^H and ^13^C NMR spectroscopy, high resolution mass spectrometry, and FT-IR spectroscopy (Figures S1–S25). Noticeably, the average of the CO stretching frequencies measured in solution for **Re-T-Tapy-Me** and **Re-T-Tapy-Et** (ν_C=O_~1940 cm^−1^) were lower than for **Re-Tapy** (ν_C=O_~1960 cm^−1^). The evolution of this parameter, which reflects the electron density on the metal center, confirms that the introduction of the mesoionic carbene leads to an increase in the overall donor capacity of the organic ligand [39]. The purity of the complexes was verified by elemental analysis.

### 2.2. Crystal Structures

Single crystals suitable for X-ray analysis were successfully grown for all new complexes from dimethylsulfoxyde (DMSO)/petroleum ether (**Re-Tapy**), CH_2_Cl_2_ (**Re-T-Tapy-Me**), acetone (**Re-T-Tapy-Et**), and DMSO/heptane (**Re-T-Pyta_(1,2,3)_-Et**).

The coordination sphere of all complexes has a quasi-octahedral geometry, as confirmed by the distortion parameters of the tapy derivatives (Table S1). The rhenium atom is coordinated with three carbonyl groups in a *fac* configuration, one chlorine atom and the nitrogen atom of the pyridyl ring. For **Re-Tapy**, the rhenium atom is also coordinated with the triazole nitrogen atom N3. It is coordinated with the C9 atom for **Re-T-Tapy-Me** and **Re-T-Tapy-Et**, and with the C10 atom for **Re-T-Pyta_(1,2,3)_-Et**. The distances and angles of the coordination sphere are quite close for the various compounds, and the pyridyl-triazole and pyridyl-triazolylidene moieties are almost planar for all compounds.

**Figure 2:**
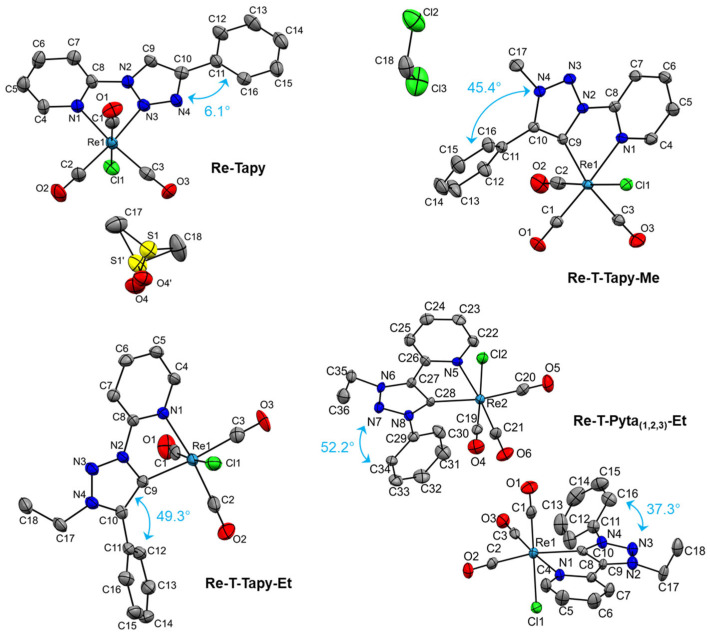
Asymmetric units of complexes **Re-Tapy**, **Re-T-Tapy-Me**, **Re-T-Tapy-Et**, and **Re-T-Pyta_(1,2,3)_-Et** with the dihedral angle α indicated in blue ink. Hydrogen atoms are not represented for the sake of clarity. Displacement ellipsoids are drawn at 30% probability.

A striking feature of the complex geometry is that the whole organic ligand of **Re-Tapy** is almost flat, with a dihedral angle α between the phenyl ring and triazole moiety as small as 6°. In this respect, **Re-Tapy** differs greatly from the previously studied isomers **Re-Pyta_(1,2,3)_** and **Re-Pyta_(1,2,4)_**, in which these moieties were twisted by 34.6° [32] and ~68° [24], respectively. For the triazolylidene derivatives **Re-T-Tapy-Me** and **Re-T-Tapy-Et**, this dihedral angle α is 45.4° and 49.3°, respectively, indicating that the alkyl group induces significant steric hindrance on the phenyl ring (Figure 2). The crystal structures are stabilized by weak hydrogen bonds, including C–H∙∙∙Cl, C–H∙∙∙O and C–H∙∙∙N type short contacts (Table S2). C–H∙∙∙*π* (Table S3) and *π*∙∙∙*π* stacking interactions (Table S4) further stabilize the 3D networks. In the **Re-Tapy** crystal lattice, a DMSO molecule connects two Re(I) complexes, forming an infinite staircase-like 1D chain, propagating in the *ac* direction (Figure S26). neighboring 1D chains interact further via parallel-displaced *π*_(Ph)_···*π*_(py)_ stacking interactions between the phenyl and pyridine rings of two adjacent molecules with a centroid-to-centroid distance of ca. 3.96 Å. Weak C17–H17_(DMSO)_···*π*_(Ph)_ intramolecular interactions were also detected (Figure S27). In **Re-T-Tapy-Me**, two complex molecules and two DCM molecules self-assemble to form a tetramer. Then, two adjacent complex molecules in antiparallel array, each belonging to a distinct tetramer, interact via two C5–H5_(py)_···O3_(CO)_ intermolecular interactions. They thus form a cyclic dimer with $R_{2}^{2}(14)$ motif, and connect the tetramers that arrange in a 2D supramolecular layer along the ab plane (Figure S28). Moreover, a weak C16–H16_(Ph)_···*π*_(trz)_ intermolecular interaction is observed in this structure. Remarkably, no *π*∙∙∙*π* stacking interaction is detected. Finally, in the **Re-T-Tapy-Et** crystal, C4–H4_(py)_···O3_(CO)_ intermolecular interactions link molecules into centrosymmetric dimers, creating $R_{2}^{2}(12)$ ring motifs. In addition, these dimers are connected by C14–H14_(Ph)_···O2_(CO)_ and C15–H15_(Ph)_···Cl1 intermolecular interactions, forming a three-dimensional network. The parallel-displaced *π*_(py)_···*π*_(py)_ stacking interactions between pyridine rings of adjacent molecules (with an intercentroid distance of *ca*. 3.49 Å) and the weak C16–H16···*π*_(Ph)_ intermolecular interactions contribute to stabilizing the crystal structure (Figure S29).

**Figure 3:**
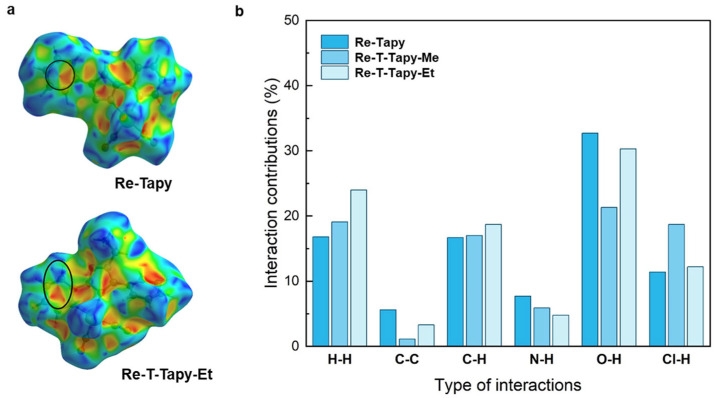
(**a**) Hirshfeld surfaces of **Re-Tapy** and **Re-T-Tapy-Et** plotted over shape index. (**b**) Comparison of contributions of various interactions to the HS of **Re-Tapy**, **Re-T-Tapy-Me**, and **Re-T-Tapy-Et**.

Hirshfeld surfaces [47] were used to visualize and quantify intermolecular interactions, as specified in the ESI. They were mapped over *d*_norm_, *d*_i_, *d*_e_ (Figure S30), curvedness (Figure S31) and shape-index (Figure 3a). The two-dimensional fingerprint plots for global interactions and individual interactions in crystal packing are given in Figures S32–S34. The HS analysis confirms the nature of the intermolecular interactions detailed above, and, in particular, the existence of *π*···*π* stacking interactions, which appear as characteristic complementary red/blue triangles on the shape index map of **Re-Tapy** and **Re-T-Tapy-Et**, while this pattern was not detected for **Re-T-Tapy-Me** (Figure 3a). The percentage contributions of the various intermolecular interactions to HS are shown in Figure 3b for the three complexes.

Finally, molecules of **Re-T-Pyta_(1,2,3)_-Et** were considered. They are strongly twisted, the phenyl-triazole dihedral angle α being ~64° instead of ~40° for **Re-T-Pyta_(1,2,3)_-Me [32]**. In some respects, the molecular arrangement of these two complexes is quite similar (Figure S35). In the asymmetric unit, the **Re-T-Pyta_(1,2,3)_-Et** molecules are displayed on two separate planes. C-H···O intermolecular interactions take place between the phenyl ring of a molecule and the oxygen atom of the equatorial carbonyl group of a neighboring molecule, and induce the formation of an infinite one-dimensional chain. Numerous C-H···Cl intermolecular interactions stabilize the 3D structure. A *π*∙∙∙*π* stacking interaction was detected between the triazole ring of one molecule and the pyridyl ring of a neighboring molecule, despite the molecular slippage. The presence of an additional methylene group thus induces significant variations in molecular geometry and crystal arrangement.

### 2.3. Electronic Properties

**Figure 4:**
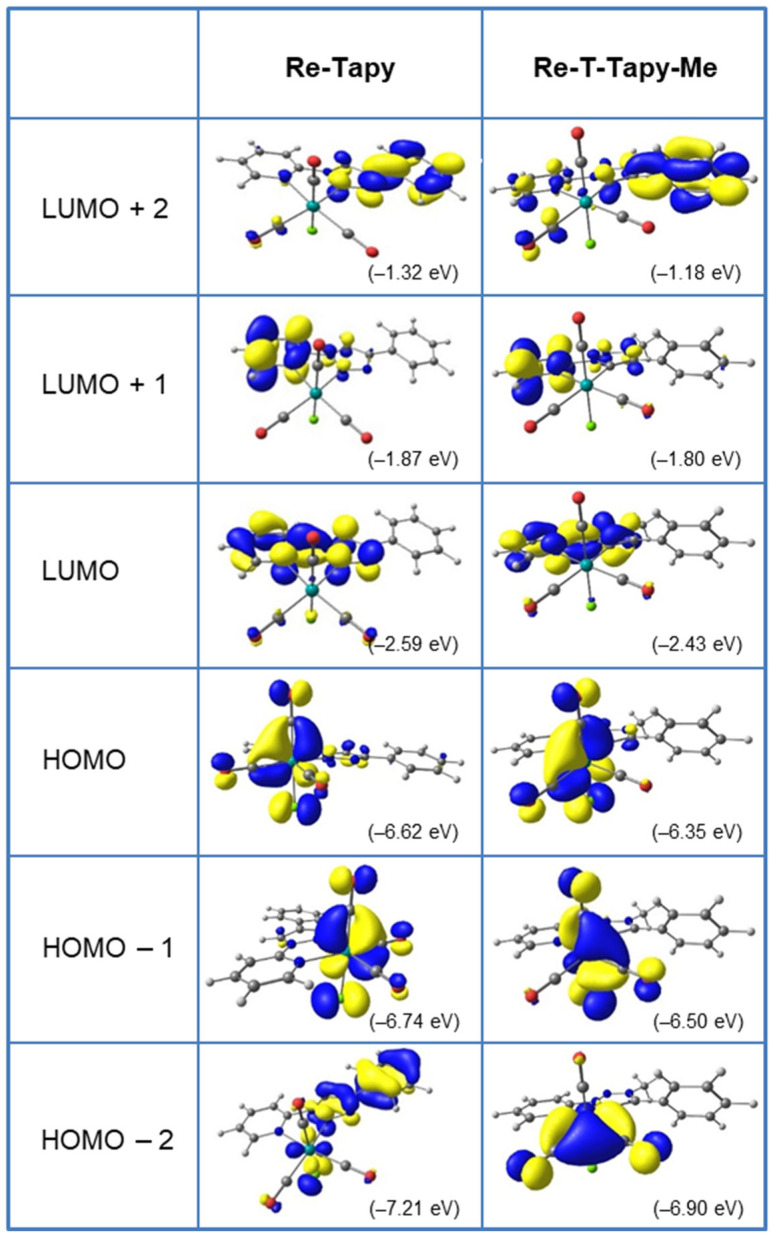
Isodensity plots (isovalue = 0.03 e bohr^−3^) and energy levels of the first frontier molecular orbitals, for complexes **Re-Tapy** and **Re-T-Tapy-Me** in DCM, according to DFT calculations at the PBE1PBE/LANL2DZ level of theory.

Computational studies of **Re-Tapy** and **Re-T-Tapy-Me** complexes in dichloromethane were performed using the time-dependent density functional theory (TD-DFT) method. The complete data are given and commented in the ESI (Tables S5–S16, Figures S36–S45). The electronic properties of **Re-T-Tapy-Et** can be considered very close to those of its methylated analog. Similarly, the properties of **Re-T-Pyta_(1,2,3)_-Et** should be almost identical to those of **Re-T-Pyta_(1,2,3)_-Me**, reported in a previous work [32]. The calculated bond lengths and angles of **Re-Tapy** and **Re-T-Tapy-Me** were in excellent agreement with the experimental data (Tables S5 and S6), as were their FT-IR spectra (Figures S44 and S45). There are great similarities in the composition of the frontier molecular orbitals of these complexes (Tables S8 and S9, Figure 4 and Figures S36 and S37). The electron density of the two highest occupied molecular orbitals (HOMO, H − 1) is located on the rhenium atom and on the chloride and carbonyl ligands, as is generally the case for tricarbonylrhenium(I) complexes. The difference between the complexes is due to the inverted composition of the H − 2 and H − 3 orbitals. In both complexes, the electron density of the LUMO and L + 1 is centered on the tapy/triazolylidene-pyridyl (trzpy) moieties, that of the L + 2 orbital is shifted towards the phenyl ring, and that of the higher orbitals L + 3 to L + 5 is centered on the Re(CO)_3_ fragment.

The most active low-energy transitions come from a metal-to-ligand charge transfer (MLCT), halogen-to-ligand charge transfer (XLCT) and ligand-to-ligand charge transfer (LLCT) admixture. At higher energy, very active transitions of various nature appear, including those having strong intra (organic) ligand charge transfer (ILCT) character (Tables S10 and S11).

This assignment was supported by the analysis of natural transition orbitals (NTOs) [48] based on calculated transition density matrices. In this method, the occupied and unoccupied NTOs are referred to as ‘hole’ and ‘electron’ transition orbitals, respectively, and optical excitations occur from the ‘hole’ to the ‘electron’ transition orbitals. NTOs provide a concise way of interpreting the transition between the ground state and the excited state by reducing the excitation to its most significant orbital contributions. This facilitates the understanding of the nature of excited states, especially when multiple transitions are involved. NTOs for **Re-Tapy** and **Re-T-Tapy-Me** are presented in Figures S40 and S42. For both complexes, the ‘hole’ NTOs associated with the lowest-energy absorption bands are mainly localized on the rhenium center, carbonyl ligands and chloride ligand, while the ‘electron’ NTOs are largely delocalized over the diimine ligand π* orbitals. This spatial distribution indicates that low-energy absorptions can be characterized as an admixture of ^3^MLCT, ^3^XLCT and ^3^LLCT states. At higher energy levels, absorptions are characterized by ^1^ILCT and purely intraligand (^1^IL) transitions.

The HOMO-LUMO gap (Table S16) of **Re-Tapy** (4.03 eV) is only slightly greater than that of **Re-Pyta_(1,2,4)_** (4.01 eV) [27] and much less that of **Re-Pyta_(1,2,3)_** (4.43 eV) [32]. This indicates that, among the three isomers, chemical stability increases in the following order: **Re-Pyta_(1,2,4)_** ≤ **Re-Tapy** < **Re-Pyta_(1,2,3)_**. The same trend is observed between the **Re-T-Tapy-Me** (3.92 eV) and **Re-T-Pyta_(1,2,3)_-Me** (4.12 eV) [32] triazolylidene complexes. The comparison between **Re-Tapy** and **Re-T-Tapy-Me** also confirms that triazolylidene-based complexes are less kinetically stable and more chemically reactive than their triazole analogs.

### 2.4. UV-Visible Absorption and Emission Properties

**Figure 5:**
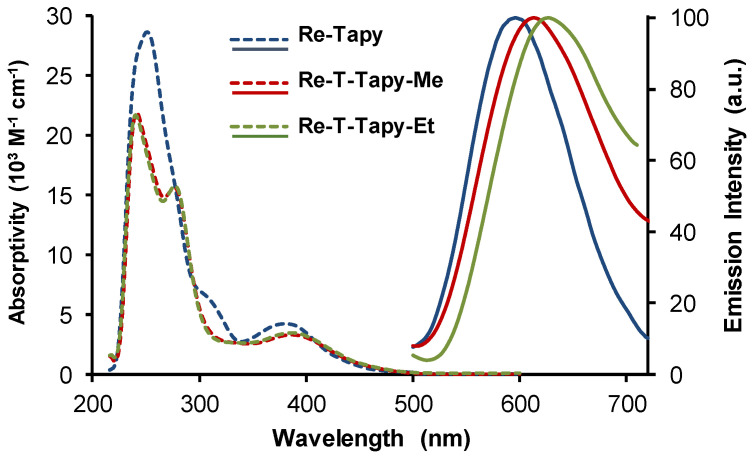
UV-vis absorption spectra of **Re-Tapy**, **Re-T-Tapy-Me**, and **Re-T-Tapy-Et** (~4 × 10^−5^ M) in dichloromethane and normalized emission spectra of these complexes (~2 × 10^−5^ M) in aerated (solid lines) solutions. λ_ex_ = 390 nm.

Although several triazolylidene-based tricarbonylrhenium complexes have been reported to be light reactive [49,50,51], no photochemical instability was detected here during spectroscopic measurements. The dilute dichloromethane solutions of **Re-Tapy**, **Re-T-Tapy-Me**, and **Re-T-Tapy-Et** were yellow in daylight. Experimental absorption spectra were recorded. Those of **Re-Tapy** and **Re-T-Tapy-Me** were in good agreement with the calculated ones and allowed precise attributions (Figures S40 and S41). For **Re-Tapy**, an intense unresolved band was observed at high energy, followed by a shoulder and a moderately intense ^1^MLCT/^1^LLCT band mainly situated in the near-UV range (Figure 5 and Table 1). For the two triazolylidene complexes, two distinct peaks can be distinguished in the high-energy band and the ^1^MLCT/^1^LLCT band was slightly shifted towards the visible region.

When illuminated by UV light (365 nm), the three complexes emitted very weakly. The emission spectra showed a single unresolved band (Figure 5). For **Re-Tapy**, the orange emission had a maximum around 600 nm, intermediate between the yellow-green emission of the **Re-Pyta_(1,2,3)_** isomer (λ_em_ = 546 nm), and the red emission of **Re-Pyta_(1,2,4)_** (λ_em_ = 626 nm). The emission quantum yield of **Re-Tapy** was about 2.2 × 10^−2^, close to that of the other two isomers, but the emission lifetime greater than 300 ns, characteristic of a phosphorescence emission, was significantly longer. The comparison of the deactivation rate constants *k_r_* and *k_nr_* shows that, among the three isomers, **Re-Tapy** is the least intrinsically emissive, but also the one for which the minimum energy is dispersed in non-radiative deactivation processes. The emission quenching effect of dissolved dioxygen was measured. The emission intensity of **Re-Tapy** was multiplied by 5.4 after bubbling the solution with argon for 3 min. This effect is by far the strongest observed among the three isomers, probably because the lifetime of the excited triplet state of **Re-Tapy** is long enough to promote significant intermolecular energy transfer to dioxygen.

Regarding the triazolylidene complexes, the emission maxima of **Re-T-Tapy-Me** and **Re-T-Tapy-Et** were significantly shifted to red (614 nm and 624 nm, respectively) relative to **Re-Tapy**. This trend was predicted by DFT and TD-DFT calculations through the analysis of spin-density distributions (Figure S38) and natural transition orbitals (NTOs) (Figures S41 and S43) in the T_1_ state, under the assumption that emission originates from the lowest ^3^MLCT/^3^LLCT state, although the experimental wavelength values were longer than those calculated (Table S13). The emission quantum yields were three times weaker than for **Re-Tapy**. The decays were triexponential (Figure S46). Both triazolylidene complexes had a lifetime of about 25 ns, which can be assigned to solubilized molecules. A long lifetime, the contribution of which is strongly variable, can be attributed to the presence of aggregates that emit much more strongly than solubilized molecules (*vide supra*). Finally, a small contribution of fluorescence is particularly visible when recording emission at short wavelengths. The origin of this fluorescence emission was not determined. Tricarbonylrhenium(I) complexes are well known for their complicated photophysics involving several mixed excited states [52]. Furthermore, although the compounds have been carefully purified, the presence of trace impurities that perturb the very weak emission signal cannot be excluded. The calculation of the deactivation constants showed that triazolylidene derivatives lose a lot of excitation energy via non-radiative deactivation pathways, as is also the case in the pyta_(1,2,3)_ series.

As concerns the effect of dioxygen, the emission intensity of **Re-T-Tapy-Me** and **Re-T-Tapy-Et** was multiplied by a factor of 2.9 and 1.6, respectively, for argon-bubbled solutions. It appeared that the position of the emission spectra was concomitantly shifted to blue, so that the emission spectra of argon-bubbled solutions of both complexes were 604 and 608 nm, respectively. This effect was perfectly reversible by aerating the solutions again. It could indicate an unusual sensitivity of our complexes to dioxygen, and would deserve to be confirmed with compounds that emit more intensively, for a more precise measurement. 

The batch-to-batch reproducibility was very good for spectroscopic studies in solutions. However, small variations were observed for solid-state emission, which is highly dependent on crystallization procedures. The values given below are an average of many measurements. 

**Figure 6:**
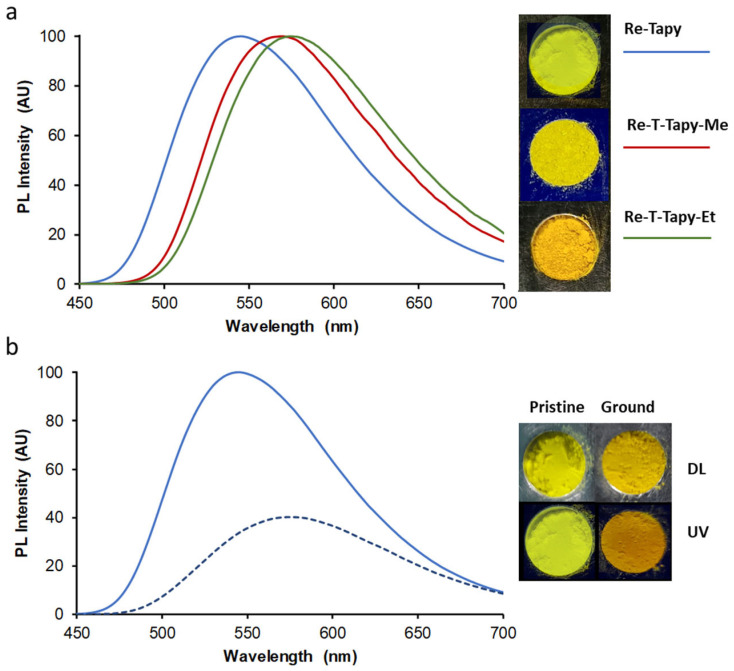
(**a**) Normalized emission spectra of **Re-Tapy** (blue line), **Re-T-Tapy-Me** (red line) and **Re-T-Tapy-Et** (green line) as pristine powders (λ_ex_ = 380 nm) and images of the corresponding samples under UV illumination (365 nm). (**b**) Emission spectra of **Re-Tapy** as pristine powder (solid line) and after grinding (dotted line). The spectrum height at the emission maximum is proportional to PLQY. Inset: images of the corresponding samples in the daylight and under UV illumination.

All tapy complexes were emissive in the form of microcrystalline powders (Table 2 and Figure 6a). The emission band showed no vibrational resolution. **Re-Tapy**, which emits a bright yellow-green light, has an emission maximum at 545 nm and an excellent PL quantum yield (PLQY) of 0.62. The triazolylidene complexes emitted orange-yellow light, with a maximum at 569 nm and 574 nm for **Re-T-Tapy-Me** and **Re-T-Tapy-Et**, respectively, and a moderate PLQY equal to or less than 0.1. Compared to solutions, the PL spectra of solid complexes were strongly blue-shifted and much more intense. Lifetimes were above microseconds, indicating a strong stabilization of the excited state responsible for emission. The origin of this clear SLE effect is the same in all three cases. The stiffening of the medium and the absence of solvent prevent non-radiative deactivations, which are in solution mainly due to the vibrations of the carbon skeleton around the metal, vibrations of the CO bonds and multiple interactions with solvent molecules [53,54,55]. In addition, the molecular arrangement is favorable to light emission. In this regard, the planar **Re-Tapy** molecules have very little overlap in the crystals so that only one *π*···*π* stacking interaction, potentially detrimental to emission, was detected. The comparison with isomers shows that **Re-Tapy** is the best solid-state emitter after **Re-Pyta_(1,2,3)_**, for which no *π*···*π* stacking interaction was detected. It turns out to be a much better emitter than **Re-Pyta_(1,2,4)_**, whose twisted structure initially seemed optimal for the spacing of molecules. Regarding triazolylidene derivatives, the better PLQY of **Re-T-Pyta_(1,2,3)_-Et** compared to **Re-T-Pyta_(1,2,3)_-Me** can be explained by the absence of *π*···*π* stacking interactions in the former case. Qualitatively, these molecules behave like their **Re-T-Pyta_(1,2,3)_** triazolylidene analogs, although they are less emissive in the solid state.

The influence of a mechanical stimulus was studied by grinding the pristine powders of the complexes with a pestle in a mortar, to induce partial amorphization of the samples. The ground powder of **Re-Tapy** turned orange in daylight as emission was red shifted by 30 nm and became much weaker (Figure 6b). This effect was perfectly reversible upon THF fuming.

**Re-Tapy** therefore exhibits significant mechanoresponsive luminescence (MRL) behavior. By comparison, the **Re-Pyta_(1,2,4)_** isomer only showed a moderate MRL effect with a spectral shift less than 16 nm, and **Re-Pyta_(1,2,3)_** showed none. The **Re-T-Tapy-Me/Et** triazolylidene derivatives showed a weak MRL effect with wavelength shift around 6 nm (Figure S47). The latter was slightly more pronounced for the **Re-T-Pyta_(1,2,3)_-Me/Et** isomers, which also have better PL efficiency (Figure S48).

**Figure 7:**
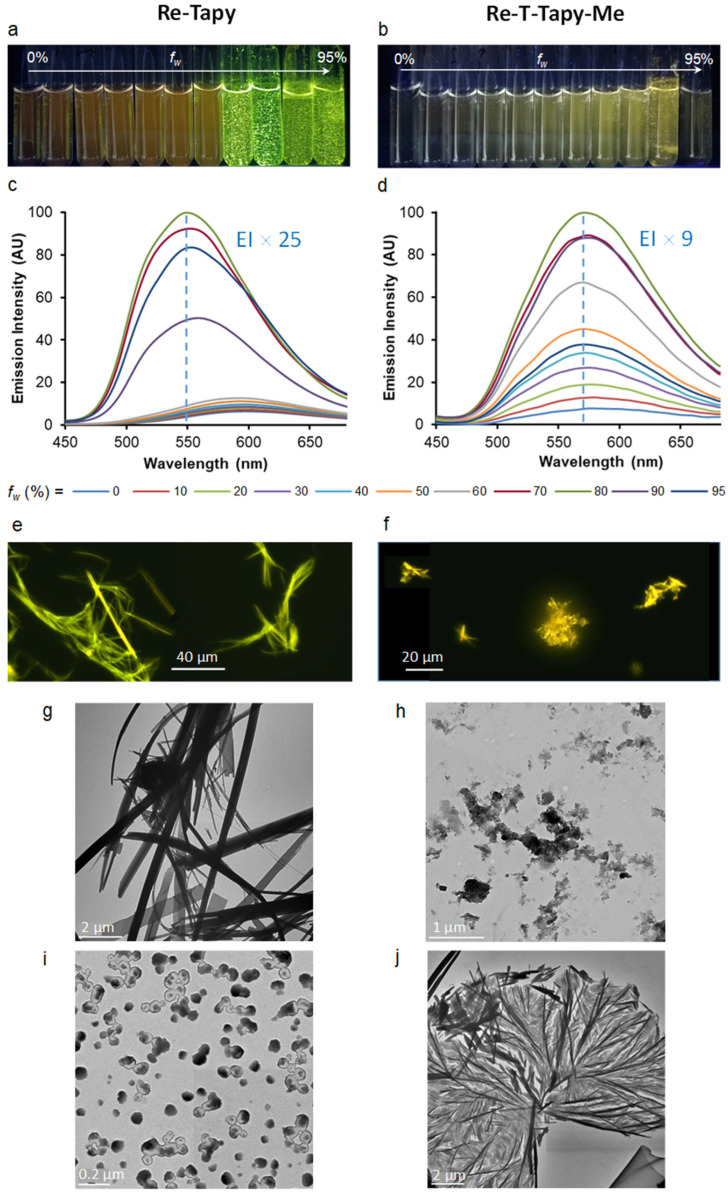
Samples of (**a**) **Re-Tapy** (5 × 10^−5^ M) and (**b**) **Re-T-Tapy-Me** (5 × 10^−5^ M) in acetonitrile solutions containing 0 to 95% water, illuminated by UV light (365 nm); (**c**,**d**) evolution of the corresponding normalized emission spectra (*λ*_ex_ = 380 nm and 360 nm, respectively, same color code); (**e**,**f**) fluorescence microscopy images of the suspensions at *f_w_* = 80%. (**g**,**h**) TEM images of the suspensions at *f_w_* = 80%. (**i**,**j**) TEM images of the suspensions at *f_w_* = 95%.

It was shown in previous work that the **Re-Pyta_(1,2,4)_** complex and its derivatives, as well as **Re-Pyta_(1,2,3)_** and **Re-T-Pyta_(1,2,3)_-Me**, form aggregates in aqueous medium. Their spectroscopic performance is parallel to that of pristine powders [19,32]. Therefore, it seemed instructive to test the AIPE properties of **Re-Tapy** and **Re-T-Tapy-Me**, to see if they behave similarly to their isomers. To do so, these complexes were dissolved in acetonitrile, where they were weakly emissive (*Φ*_P_ = 8.7 × 10^−3^ and 3.6 × 10^−3^, respectively) and chemically and photochemically very stable (virtually no change in the UV-vis spectra was detected after a 3-day irradiation with a 385 nm emitting diode). Then, the water fraction *f*_w_ in acetonitrile solutions was gradually increased. For **Re-Tapy**, the weak orange-red emission centered at 594 nm abruptly turned into a strong green-yellow emission with a maximum at 548 nm for *f*_w_ > 80%. The emission intensity was then further reduced at high water fractions (Figure 7a,c). In contrast, the emission of **Re-T-Tapy-Me** at 576 nm in pure acetonitrile was only shifted to 570 nm with a gradual increase in intensity up to *f*_w_ = 90%, followed by a decrease at *f*_w_ = 95% (Figure 7b,d). The intensity of the PL signal at the maximum wavelength of the most intense band was multiplied by 25 and 9 for **Re-Tapy** and **Re-T-Tapy-Me**, respectively.

If we compare the spectroscopic properties of suspensions of the two complexes with those of their corresponding pristine powders, it appears that the maximum emission wavelengths are similar. The quantum yield values are quite close for **Re-Tapy**, but different for **Re-T-Tapy-Me**, which is significantly less emissive in suspension than in powder form. The lifetimes of the suspensions are systematically shorter than those of the powders. Their distribution is reminiscent of that of the powder for **Re-Tapy**, while it is close to that of the dissolved complex for **Re-T-Tapy-Me**. Most likely, the emission of suspensions arises from solid particles in both cases, but **Re-T-Tapy-Me** is less hydrophobic than **Re-Tapy** and forms fewer particles.

Evidence was then given for the formation of particles. The spectroscopic changes were accompanied by large variations in absorption spectra, typical for aggregation (Figure S49). The samples became cloudy and particles were detected by the naked eye for **Re-Tapy**. Fluorescence microscopy of the **Re-Tapy** suspension at *f*_w_ = 80% suggested the presence of highly emissive microfibrils (Figure 7e). Transmission electronic microscopy TEM) confirmed the presence of thin particles that measure between 1.4 µm long and 80 nm wide, and assemble to give elongated shapes of many tens of µm long (Figure 7g and Figure S52†). Interestingly, only spherical nanoparticles measuring less than 100 nm were observed in suspensions at *f*_w_ = 95% (Figure 7j and Figure S53). Probably, in this case, the Re-Tapy molecules precipitate easily and do not have time to organize. The different nature of the particles formed may explain the emission decrease observed at high water content. Meanwhile, suspensions of Re-T-Tapy-Me at *f*_w_ = 80% observed under the fluorescence microscope showed some rare agglomerates of elongated particles (Figure 7f). TEM revealed diffuse shapes where small rectangular objects, probably microcrystals, can be distinguished (Figure 7h and Figure S54). On the other hand, at *f*_w_ = 95%, long fibers measuring several tens of µm were observed (Figure 7i and Figure S55). For this weakly hydrophobic complex, the formation of long particles is favored by a high water content, and it is not associated with strong emission. Dynamic light scattering (DLS) measurements on filtered suspensions confirmed in most cases the presence of particles with an average size less than 150 nm (Table S17).

In summary, a clear AIPE effect was obtained for both complexes, but it was much stronger for **Re-Tapy**, the complex that is the best emitter in powder form. This study confirms the hypothesis of the link between the two areas.

## 3. Conclusions

This work is the third part of our study on the influence of isomerism in photoluminescent rhenium complexes. It confirms that isomerism does play a very important role. It was previously shown in pyta series that the dihedral angle between the phenyl and triazole rings modulates the solid-state emission properties [26,32]. The hypothesis that had been made is that the very good PL properties observed for **Re-Pyta_(1,2,4)_** and **Re-Pyta_(1,2,3)_** are related to the torsion of the organic ligand, which allows a more favorable molecular arrangement, with little *π*∙∙∙*π* stacking [24,25,26,27,31,32,33,56]. Indeed, **Re-Pyta_(1,2,3)_**, for which no *π*∙∙∙*π* staking has been detected, is the most emissive isomer [32]. Unexpectedly, the present work shows that **Re-Tapy** also has a favorable arrangement and very good solid-state emission despite the practically planar geometry of its organic ligand. This shows how difficult it is to predict the PL behavior of a molecule. It appeared that **Re-Tapy** has very good value for AIPE-related applications. This observation sheds a new light on the behavior of its derivatives reported in the literature, for which the strong emission increase in aqueous solution depends on hydrophobicity [41,42,43,44]. It would now be necessary to know whether the good SLE and AIPE properties of **Re-Tapy** are fully preserved after substitution of this molecule, which is not the case for the derivatives of **Re-Pyta_(1,2,4)_** and **Re-Pyta_(1,2,3)_** that we have studied so far [19,32]. The three isomers would also deserve to be studied in polymer matrices that are relevant for practical use in the fields of sensors and optoelectronics [7,8,9,10,11,12,13,14]. Thanks to its ease of synthesis, **Re-Tapy** could then become a very good basic pattern for future applications.

This study also confirms that, like their pyta_(1,2,3)_ isomers, triazolylidenes **Re-T-Tapy-Me** and **Re-T-Tapy-Et** do not have very attractive spectroscopic properties. On the other hand, the origin of their photochemical stability, compared to the reactivity of closely related complexes in the literature, deserves to be better understood. In general, it would be instructive to perform for all our compounds temperature-dependent spectra and transient photoluminescence spectroscopy (which exceed our equipment facilities) to further explore the nature of the excited states involved and the mechanisms underlying non-radiative emission and decays. Given the rich photophysics of rhenium complexes, this type of exploration is extremely fruitful [7,8,9,10,11,12,13,14,57,58]. It would also be interesting to test these triazolylidene-based complexes in therapeutic chemistry to see if they are as promising in this area as their pyta_(1,2,3)_ counterparts [32].

## 4. Experimental Section

### 4.1. General Methods

All purchased chemicals were of the highest purity commercially available and were used without further purification. All synthesis experiments were conducted under argon atmosphere. The reactions were monitored by TLC on silica gel Alugram^®^ Xtra SIL G/UV254 (Macherey–Nagel, Hoerdt, France). Column chromatography was performed on Macherey–Nagel silica gel. NMR, mass and infrared spectra were obtained in the ‘Services communs de l’Institut de Chimie de Toulouse’. NMR spectra were recorded on a Bruker Avance 300 MHz instrument (Bruker France SAS, Wissembourg, France), with chemical shifts reported in ppm and referenced using residual solvent signals as internal standards. The signals are described as follows: s, singlet; d, doublet; t, triplet; and m, multiplet. High-resolution mass spectrometry (HRMS) data was obtained using a Xero G2 QTOF (Waters) instrument (Waters Corporation, Manchester, UK). Infrared spectra were recorded on a Thermoscientific Nicolet iS50 FT-IR spectrometer (Madison Connecticut, USA). Microanalyses were performed at the ‘Service d’analyse du Laboratoire de Chimie de Coordination de Toulouse (LCC)’ using a Perkin Elmer 2400 series II analyzer (Perkin Elmer Scientific (France) SAS, Villepinte, France).

### 4.2. Synthesis

1-Phenyl-4-(2-pyridyl)-1,2,3-triazole (**L-Pyta_(1,2,3)_**) [59], 2-(4-phenyl-1,2,3-triazol-1-yl)pyridine (**L-Tapy**) [43], and complex **Re-Tapy** [43] were prepared as described in the literature.

- 2-(1-phenyl-1*H*-1,2,3-triazol-4-yl)pyridine-N-oxide, (**1**)

A mixture of **L-Pyta_(1,2,3)_** (1 eq; 364 mg, 1.64 mmol) and *m*-CPBA (2 eq; 566 mg, 3.28 mmol) in CHCl_3_ (28 mL) was refluxed at 62 °C for 30 min. After cooling to rt, a 0.5 M aqueous solution of Na_2_S_2_O_3_ solution (50 mL) was added to the reaction mixture. After stirring for 10 min, the product was extracted with CH_2_Cl_2_ (2 × 50 mL). The combined organic layers were washed with aqueous NaOH (1 M) and concentrated under vacuum to yield pure compound **1** as a white solid (341 mg, 1.43 mmol, 87%). ^1^H NMR (300 MHz, CDCl_3_) δ: 9.48 (s, 1H), 8.58 (dd, *J =* 8.2, 1.8 Hz, 1H), 8.36–8.34 (m, 1H), 7.86–7.83 (m, 2H), 7.59–7.52 (m, 2H), 7.49–7.39 (m, 2H), 7.29–7.23 (m, 1H); ^13^C NMR (75 MHz, CDCl_3_) δ: 141.1, 140.0, 139.4, 136.8, 129.8, 128.9, 125.8, 124.24, 124.20, 124.0, 120.6; HRMS (ESI): *m*/*z* 239.0940 [M + H]^+^ (calc. for C_13_H_11_N_4_O 239.0933).

- 3-Ethyl-1-phenyl-4-(pyridin-2-yl)-1*H*-1,2,3-triazolium tetrafluoroborate, (**L-T-Pyta_(1,2,3)_-Et**)

A mixture of **1** (1 eq; 150 mg, 0.63 mmol) and Et_3_OBF_4_ (4 eq; 479 mg, 2.52 mmol) in dry CH_2_Cl_2_ (2 mL) was stirred at rt for four days. After evaporation of CH_2_Cl_2_, the residue was dissolved in EtOH (26 mL) and [Mo(CO)_6_] (1 eq; 167 mg, 0.63 mmol) was added. The mixture was refluxed for 1 h. The solvent was removed and the product was purified by silica gel column chromatography (DCM/MeOH 10:0.2 *v*/*v*), affording pure **L-T-Pyta-Et** (156 mg, 0.46 mmol, 73%) as a white solid. ^1^H NMR (300 MHz, CDCl_3_) δ: 9.71 (s, 1H), 8.77 (ddd, *J* = 4.8, 1.7, 0.9 Hz, 1H), 8.43 (d, *J* = 8.0 Hz, 1H), 8.10–8.07 (m, 2H), 8.03 (td, *J* = 7.9, 1.8 Hz, 1H), 7.73–7.66 (m, 3H), 7.52 (ddd, *J* = 7.7, 4.8, 1.0 Hz, 1H), 5.31 (q, *J* = 7.0 Hz, 2H), 1.77 (t, *J* = 7.2 Hz, 3H); ^13^C NMR (75 MHz, CDCl_3_) δ: 149.7, 142.5, 141.4, 138.8, 134.6, 132.3, 130.6, 127.5, 126.1, 126.0, 121.4, 50.3, 14.4; HRMS (ESI): *m*/*z* 251.1299 [M]^+^ (calc. for C_15_H_15_N_4_ 251.1297).

- Tricarbonylrhenium(I) complex [Re(CO)_3_(L-T-pyta_(1,2,3)_-Et)Cl], (**Re-T-Pyta_(1,2,3)_-Et**)

To a suspension of ligand **L-T-Pyta_(1,2,3)_-Et** (1 eq; 86 mg, 0.254 mmol) and Re(CO)_5_Cl (1.1 eq; 102 mg, 0.28 mmol) in toluene (13 mL), an excess of triethylamine (0.7 mL) was added. The reaction mixture was then refluxed for three days. After cooling to rt, the toluene was evaporated under reduced pressure. Silica gel column chromatography (DCM/acetone 9.5:0.5 *v*/*v*) afforded pure **Re-T-Pyta_(1,2,3)_-Et** as a yellow solid (139 mg, 0.248 mmol, 98%). ^1^H NMR (300 MHz, CDCl_3_) δ: 9.20–9.18 (m, 1H), 8.04 (td, *J* = 7.9, 1.6 Hz, 1H), 7.97–7.91 (m, 2H), 7.80 (d, *J* = 8.1 Hz, 1H), 7.64–7.59 (m, 3H), 7.41 (ddd, *J* = 7.6, 5.5, 1.2 Hz, 1H), 4.89–4.82 (m, 2H), 1.83 (t, *J* = 7.3 Hz, 3H); ^13^C NMR (75 MHz, CDCl_3_) δ:198.3, 197.4, 189.7, 184.9, 156.2, 149.8, 149.0, 139.1, 138.7, 130.8, 129.8, 125.8, 125.4, 120.6, 48.0, 14.1; HRMS (ESI): *m*/*z* 521.0627 [M − Cl]^+^ (calc. for C_18_H_14_N_4_O_3_Re: 521.0624), *m*/*z* 574.0655 [M + NH_4_]^+^ (calc. for C_18_H_18_N_5_O_3_ClRe: 574.0647). Anal. calcd (%) for C_19_H_16_N_4_O_3_Cl_3_Re (C_18_H_14_N_4_O_3_ClRe + CH_2_Cl_2_): C 35.61, H 2.52, N 8.74; found: C 35.71, H 2.13, N 8.08. FT-IR (CH_2_Cl_2_) ν_C=O_: 2016, 1915, 1885 cm^−1^.

- 3-Methyl-4-phenyl-1-(pyridin-2-yl)-*1H*-1,2,3-triazol-3-ium triflate, (**L-T-Tapy-Me**)

Ligand **L-Tapy** (1 eq; 333 mg, 1.5 mmol) in dry DCM (4.5 mL) was purged with dry argon with stirring at 0 °C for a few minutes. Methyl triflate (F_3_CSO_3_CH_3_) (1.5 eq; 369 mg, 0.25 mL, 2.25 mmol) was added and the mixture was stirred at 0 °C for an additional 30 min and after that at rt for 1 day. The solvent was removed and the product was purified by silica gel column chromatography (starting with petroleum ether/ethyl acetate 7:3 *v*/*v* to elute the unreacted **L-Tapy**, then DCM/MeOH 10:0.5 *v*/*v*), affording pure **L-T-Tapy-Me** (328 mg, 0.85 mmol, 57%) as a white solid. ^1^H NMR (300 MHz, CDCl_3_) δ: 9.03 (s, 1H), 8.56 (ddd, *J* = 4.8, 1.7, 0.8 Hz, 1H), 8.15 (dt, *J* = 8.2, 0.9 Hz, 1H), 8.04 (ddd, *J* = 8.2, 7.6, 1.8 Hz, 1H), 7.73–7.71 (m, 2H), 7.64–7.51 (m, 4H), 4.38 (s, 3H); ^13^C NMR (75 MHz, CDCl_3_) δ: 149.1, 146.4, 144.2, 140.4, 132.0, 129.66, 129.63, 127.0, 124.2, 121.6, 115.1, 39.3; HRMS (ESI): *m*/*z* 237.1144 [M]^+^ (calc. for C_14_H_13_N_4_ 237.1140).

- 3-Ethyl-4-phenyl-1-(pyridin-2-yl)-*1H*-1,2,3-triazol-3-ium tetrafluoroborate, (**L-T-Tapy-Et**)

Ligand **L-Tapy** (1 eq; 400 mg, 1.8 mmol) in dry DCM (5.4 mL) was purged with dry argon and stirred at 0 °C for a few minutes. Then, Et_3_OBF_4_ (1.5 eq; 513 mg, 2.70 mmol) was added and the mixture was stirred at 0 °C for an additional 30 min and after that at rt for 1 day. The solvent was removed and the product was purified by silica gel column chromatography (starting with petroleum ether/ethyl acetate 7:3 *v*/*v* to elute the unreacted **L-Tapy**, then DCM/MeOH 10:1 *v*/*v*), affording pure **L-T-Tapy-Et** (340 mg, 1.01 mmol, 56%) as a white solid. ^1^H NMR (300 MHz, CDCl_3_) δ: 8.97 (s, 1H), 8.57–8.55 (dq, 1H), 8.19–8.17 (dt, 1H), 8.08–8.02 (td, 1H), 7.70–7.67 (dt, 2H), 7.65–7.54 (m, 4H), 4.73–4.66 (q, 2H), 1.66–1.61 (t, 3H); ^13^C NMR (75 MHz, CDCl_3_) δ: 149.2, 146.8, 143.8, 140.6, 132.1, 129.9, 129.8, 127.2, 124.7, 122.0, 115.6, 48.4, 14.0; HRMS (ESI): *m*/*z* 251.1298 [M]^+^ (calc. for C_15_H_15_N_4_ 251.1297).

- Tricarbonylrhenium(I) complex [Re(CO)_3_(L-T-tapy-Me)Cl], (**Re-T-Tapy-Me**)

To a suspension of ligand **L-T-Tapy-Me** (1 eq; 200 mg, 0.52 mmol) and [Re(CO)_5_Cl] (1.1 eq; 206 mg, 0.57 mmol) in toluene (25.2 mL), an excess of triethylamine (1.4 mL) was added. The reaction mixture was then refluxed for three days. After cooling to rt, the toluene was evaporated under reduced pressure. Silica gel column chromatography (DCM/acetone 9.5:0.5 *v*/*v*) afforded pure **Re-T-Tapy-Me** as a yellow solid (277 mg, 0.51 mmol, 99%). ^1^H NMR (300 MHz, CDCl_3_) δ: 9.00 (ddd, *J* = 5.5, 1.5, 0.8 Hz, 1H), 8.24–8.12 (m, 2H), 7.75–7.70 (m, 2H), 7.63–7.56 (m, 2H), 7.52 (ddd, *J* = 7.2, 5.5, 1.6 Hz, 1H), 4.22 (s, 3H); ^13^C NMR (75 MHz, CDCl_3_) δ: 198.1, 197.2, 189.8, 179.0, 154.1, 151.9, 148.8, 141.0, 130.9, 130.5, 129.4, 126.4, 126.1, 114.5, 38.0; HRMS (ESI): *m*/*z* 543.0229 [M + H]^+^ (calc. for C_17_H_13_N_4_O_3_ClRe: 543.0225), *m*/*z* 507.0467 [M − Cl]^+^ (calc. for C_17_H_12_N_4_O_3_Re: 507.0467). Anal. calcd (%) for C_17_H_12_N_4_O_3_ClRe + 0.25 CH_3_COCH_3_: C 38.31, H 2.45, N 10.07; found: C 38.57, H 1.87, N 10.06. FT-IR (CH_2_Cl_2_) ν_C=O_: 2017, 1919, 1886 cm^−1^.

- Tricarbonylrhenium(I) complex [Re(CO)_3_(L-T-Tapy-Me)Cl], (**Re-T-Tapy-Et**)

To a suspension of ligand **L-T-Tapy-Me** (1 eq; 200 mg, 0.59 mmol) and [Re(CO)_5_Cl] (1.1 eq; 235 mg, 0.65 mmol) in toluene (29 mL), an excess of triethylamine (1.6 mL) was added. The reaction mixture was then refluxed for three days. After cooling to rt, the toluene was evaporated under reduced pressure. Silica gel column chromatography (DCM/acetone 9.5:0.2 *v*/*v*) afforded pure **Re-T-Tapy-Me** as a yellow solid (250 mg, 0.45 mmol, 76%). ^1^H NMR (300 MHz, CDCl_3_) δ: 9.01 (ddd, *J* = 5.5, 1.5, 0.8 Hz, 1H), 8.23 ((ddd, *J* = 8.3, 1.4, 0.8 Hz, 1H), 8.15 (ddd, *J* = 8.3, 7.4, 1.6 Hz, 1H), 7.72–7.67 (m, 2H), 7.62–7.55 (m, 3H), 7.52 (ddd, *J* = 7.2, 5.5, 1.5 Hz, 1H), 4.55 (qd, *J* = 7.3, 1.1 Hz, 2H). 1.65 (t, *J* = 7.3 Hz, 3H). ^13^C NMR (75 MHz, CDCl_3_) δ: 197.9, 196.9, 189.7, 178.8, 153.9, 151.9, 148.0, 140.8, 130.6, 130.3, 129.2, 126.2, 114.3, 46.5, 15.4. HRMS (ESI): *m*/*z* 521.0622 [M − Cl]^+^ (calc. for C_18_H_14_N_4_O_3_Re: 521.0623), *m*/*z* 574.0649 [M + NH_4_]^+^ (calc. for C_18_H_18_N_5_O_3_ClRe: 574.0656). Anal. calcd (%) for C_18_H_14_N_4_O_3_ClRe + 0.25 CH_3_COCH_3_: C 39.47, H 2.74, N 9.82; found: C 39.14, H 2.18, N 9.87. FT-IR (CH_2_Cl_2_) ν_C=O_: 2017, 1918, 1886 cm^−1^.

### 4.3. Crystallography

Crystallographic data were collected at low temperature (193 K) with an Oxford Instruments Cryostream 700+ Series device using MoK_α_ radiation (wavelength = 0.71073 Å) on a Bruker APEX II Quazar diffractometer equipped with a 30 W air-cooled microfocus (**Re-T-Tapy-Me** and **Re-T-Pyta_(1,2,3)_-Et**) or CuK_α_ radiation (wavelength = 1.54178 Å) on a Bruker-AXS D8-Venture diffractometer equipped with a PHOTON III-C14 detector (**Re-Tapy** and **Re-T-Tapy-Et**). Phi- and omega-scans were used. The space group was determined on the basis of systematic absences and intensity statistics. An empirical absorption correction was employed [60]. The structures were solved using an intrinsic phasing method (ShelXT 2018/2 software) [61]. All non-hydrogen atoms were refined anisotropically using the least-square method on *F*^2^ [62]. Hydrogen atoms were refined isotropically at calculated positions using a riding model. The structure of compound **Re-Tapy** was found to be disordered (solvent molecule). Several restraints (SAME, SIMU, DELU) and equal xyz and U_ij_ constraints (EXYZ and EADP) were applied to refine some moieties of the molecules and to avoid the collapse of the structure during the least-squares refinement by the large anisotropic displacement parameters.

Supplementary crystallographic data can be obtained free of charge from the Cambridge Crystallographic Data Centre via https://www.ccdc.cam.ac.uk/structures/, accessed on 28 May 2025. Selected crystallographic data are collected in Table 3.

Analysis of intermolecular interactions was performed by PLATON program (Version: 141123) [63]. Graph-set theory was used to assign supramolecular motifs [64,65,66,67,68]. Then, 3D Hirshfeld surfaces (HS) and 2D fingerprint plots (FP) [69] were generated with high resolution based on the crystallographic information file (CIF) using Crystal Explorer 17.5 software [70]. HS were mapped over d_norm_, shape-index, and curvedness. The normalized contact distance (d_norm_) is defined by d_i_ and d_e_, which are the distances from the surface to the nearest atom interior and exterior to the surface, respectively. The vdW radii of atoms is defined according to Equation (1).

(1) $$d_{norm}=\frac{d_{i}-r_{i}^{vdW}}{r_{i}^{vdW}}+\frac{d_{e}-r_{e}^{vdW}}{r_{e}^{vdW}}$$

### 4.4. Computational Details

The GAUSSIAN16 program package [71] was employed for all calculations (the geometry optimization, the ground-state and excited-state electronic structures, and optical spectra) with the aid of the ChemCraft visualization program [72]. The ground state (S_0_), the first excited state (S_1_) and the lowest triplet state (T_1_) geometries of **Re-Tapy** and **Re-T-Tapy-Me** were fully optimized with the restricted and unrestricted density functional theory (R-DFT and U-DFT) method using the Perdew–Burke–Ernzerhof PBE1PBE functional with no symmetry constraints [73]. In all calculations, the “double-ζ” quality basis set LANL2DZ with Hay and Wadt’s relative effective core potential ECP (outer-core [(5s^2^5p^6^)] electrons and (5d^6^) valence electrons) [74,75] was employed for the Re atom. The 6−311+G** basis set for H, C, N, and O atoms was used [76]. The solvent effect (dichloromethane, *ε* = 8.93) was simulated using the Self-Consistent Reaction Field (SCRF) under the Conductor Polarizable Continuum Model (CPCM) [77,78,79]. The vibrational frequencies calculations were performed using the optimized structural parameters of the complexes, to confirm that each optimized structure represents a local minimum on the potential energy surface and all eigenvalues are non-negative. To obtain a good agreement with the experimental vibrational frequencies in solid state, the theoretically calculated vibrational frequencies were scaled with a scaling factor of 0.9485. The optimized Cartesian coordinates of the complexes are included in the ESI part (see Tables S18–S23). On the basis of the optimized ground and excited state geometries, the absorption and emission properties were calculated by the time dependent density functional theory (TD-DFT) method at the PBE1PBE/LANL2DZ/6−311+G** level. These methods have already shown good agreement with experimental studies for different rhenium(I) complexes [80]. The natural transition orbitals of the low-lying singlet and triplet transitions were generated using *Chemcraft 1.8*.

### 4.5. Spectroscopy

UV-visible absorption spectra and emission spectra of the solutions were measured at 20 °C with a Xenius spectrofluorometer from SAFAS, Monaco, using cells of 1 cm optical pathway. All emission spectra were corrected. The emission quantum yields of solution (*Φ*) were determined using the classical formula:

*Φ_x_* = (*A_s_* × *I_x_* × *n_x_*^2^ × *Φ_s_*)/(*A_x_* × *I_s_* × *n*_s_^2^) (2)

where *A* is the absorbance at the excitation wavelength, *I* the integrated emission intensity, and *n* the refractive index. Subscripts *s* and *x* refer to the standard and to the sample of unknown quantum yield, respectively. Coumarin 153 (*Φ_s_* = 0.53) in ethanol was used as a standard [81]. The absorbance of the solutions was equal to or less than 0.06 at the excitation wavelength. The error on quantum yield values is estimated to be about 10%. The rate constants for radiative (*k_r_*) and nonradiative (*k_nr_*) decay were calculated using the following equation:

*k_r_* = *Φ*/*τ* and k*_n_*_r_ = (1 − *Φ)*/*τ* (3)

with *Φ* the emission quantum yield and *τ* the luminescence lifetime in solution. The photochemical stability was measured by irradiating with a fiber-coupled Nanoled-370 (Opton Laser Int., Les Ulis, France).

For AIPE measurements, ultrapure water (18 MΩ) was prepared using a Purelab Flex apparatus from Elga/Veolia, Antony, France. A small volume (30 µL) of a concentrated solution of complex in acetonitrile was injected into 2.97 mL of various acetonitrile/water mixtures. After stirring for 3 h in the dark, samples were sonicated for 5 min before optical measurement. The suspension quantum yields were measured using Equation (3), after appropriate correction of absorbance and inner-filter effect [82]. The error is estimated to be around 20%. Fluorescence microscopy was performed with a Leitz Laborlux D fluorescence microscope equipped with an Andor Luca camera (*λ*_ex_~450–490 nm, *λ*_em_ > 500 nm). Transmission electron microscopy (TEM) samples were prepared by the drop casting method onto 3.05 mm copper grids with a 400-mesh density (supplied by Pelanne Instruments, Toulouse, France). These grids were pre-coated with a collodion film approximately 20–50 nm thick. TEM images were recorded on a MET JEOL JEM 1011 instrument (JEOL SAS, Croissy-sur-Seine, France). The hydrodynamic size of particles was measured by dynamic light scattering (DLS), using a Zetasizer NanoZ device (Malvern instruments, UK). Dilute suspensions were prepared ultrapure water and filtered through a 0.45 µm hydrophilic PTFE filter prior to analysis.

Solid state spectra were recorded on a Fluorolog 3-2iHR320 spectrofluorometer from HORIBA Instruments Inc., Edison, NJ, USA, and were corrected. The absolute photoluminescence quantum yield values (*Φ_PL_*) were determined using the Xenius SAFAS spectrofluorometer, provided with an integrating sphere, by a method based on the one developed by De Mello et al. [83], as described elsewhere [24]. The error was estimated to be about 20%. Emission decay curves were recorded using the time-correlated single photon counting method (TCSPC) on the Horiba Fluorolog 3-2iHR320 spectrofluorometer equipped with a Nanoled-370 (HORIBA Jobin Yvon IBH Ltd., Glasgow, UK. λ_ex_ = 371 nm). Emitted photons were detected at 90° by means of a Hamamatsu R928 photomultiplier. Emission was recorded near the maximum with a bandpass of 4 nm. The instrumental response was recorded directly before each decay curve, using the scattering of the sample at 371 nm. All analyses were recorded using the Datastation v2.7 software from Horiba. The decay curves were analyzed with reconvolution and global non-linear least-squares minimization method using DAS6 v6.8 software. The absorbance of solutions at λ_ex_ was lower than 0.1. Solid state samples were deposited on a quartz holder.

## Figures and Tables

**Table 1:** Spectroscopic data of all complexes in DCM: Maximum absorption wavelength (*λ*_abs_), molar extinction coefficient (*ε*), maximum wavelength of phosphorescence emission (*λ*_P_), emission quantum yield (*Φ*_P_), lifetime (*τ*) with relative amplitude (*f*) and chi square (χ^2^) values, mean lifetime (<*τ*>), radiative (*k_r_*), and non-radiative (*k_r_*) rate constants. Concentrations: ~4 × 10^−5^ M for absorption, ~2 × 10^−5^ M for emission.

Compound	*λ*_abs_ (nm)	*ε* (M^−1^ cm^−1^)	*λ*_P_ (nm)	*Φ*_P_	*τ* (ns), *f* (%) [χ^2^]	*<τ>* (ns)	*k_r_* (s^−1^)	*k_nr_* (s^−1^)
**Re-Tapy**	252 306 (sh) 380	28,600 6300 3900	600	2.2 × 10^−2^	306 [1.03]	306	7.19 × 10^4^	3.19 × 10^6^
**Re-T-Tapy-Me**	242 276 386	21,800 14,700 3100	614	7.1 × 10^−3^	25 (*22.4*) 315 (*75.1*) [1.11] ^c^	248	2.86 × 10^4^	4.00 × 10^6^
**Re-T-Tapy-Et**	240 278 388	21,700 15,200 3100	626	7.5 × 10^−3^	23 (*70.4*) 272 (*21.6*) [1.12] ^c^	91	8.24 × 10^4^	1.09 × 10^7^
**Re-Pyta_(1,2,3)_** ^a^	242 278 340	20,600 18,300 4400	546	1.7 × 10^−2^	154 [1.08]	154	1.10 × 10^5^	6.38 × 10^6^
**Re-T-Pyta_(1,2,3)_-Me** ^a^	240 288 354	20,700 13,700 4400	616	9.7 × 10^−3^	47 [1.70]	47	2.06 × 10^5^	2.10 × 10^7^
**Re-T-Pyta_(1,2,3)_-Et**	240 290 366	18,700 12,100 3800	612	1.2 × 10^−2^	41 (*77.6*) 393 (*17.6*) [1.09] ^c^	101	1.18 × 10^5^	9.78 × 10^6^
**Re-Pyta_(1,2,4)_** ^b^	240 282 382	15,100 11,600 4000	626	2.0 × 10^−2^	75 [1.29]	75	2.70 × 10^5^	1.31× 10^7^

a*:* From ref. [32]; b: From ref. [24]; c: Decays are triexponential. The full decay data are given in the ESI (Figure S46). sh = shoulder.

**Table 2:** Photoluminescence maximum wavelength (*λ*_PL_), emission quantum yield (*Φ*_PL_), and lifetime (*τ*) with relative amplitude (*f*) and chi square (χ^2^) values of all complexes in the aggregated state (5 × 10^−5^ M in water/acetonitrile 80:20 *v*/*v*) and in the solid state (pristine and ground powders).

Complex	Suspensions				Pristine				Ground	
	*λ*_PL_ (nm)	*Φ*_PL_	*τ_PL_* (ns), *f* (%) [χ^2^]	*<τ>* (ns)	*λ*_PL_ (nm)	*Φ*_PL_	*τ_PL_* (ns), *f* (%) [χ^2^]	*<τ>* (ns)	*λ*_PL_ (nm)	*Φ*_PL_
**Re-Tapy**	548	0.41	1.9 (*0.9*), 115 (*3.9*) 1198 (*95.2*) [1.20]	1145	545	0.62	6.0 (*0.6*), 252 (*9.7*) 2728 (*89.7*) [1.41]	2471	575	0.25
**Re-T-Tapy-Me**	570	0.014	2.6 (*1.3*), 189 (*78.0*) 662 (*20.7*) [1.12]	285	569	0.10	5.0 (*0.9*), 243 (*19.7*) 1356 (*79.4*) [1.39]	1124	576	0.059
**Re-T-Tapy-Et**	---	---	---	---	574	0.056	24.7 (*1.6*), 305 (*19.7*) 1625 (*78.7*) [1.30]	1340	580	0.055
**Re-Pyta_(1,2,3)_**	508 ^a^	---	---	---	510 ^a^	0.69 ^a^	0.9 (*0.3*), 32 (*0.6*) 1164 (*99.1*) [1.28] ^a^	1153	513	0.50
**Re-T-Pyta_(1,2,3)_-Me**	536 ^a^	---	---	---	537 ^a^	0.16 ^a^	1.3 (*0.4*), 64 (*3.2*) 711 (*96.4*) [1.07] ^a^	687	553	0.17
**Re-T-Pyta_(1,2,3)_-Et**	---	---	---	---	545	0.21	16.7 (*0.7*), 301 (*14.9*) 1371 (*84.4*) [1.29]	1202	554	0.21
**Re-Pyta_(1,2,4)_**	562 ^b^	0.50 ^b^	16 (*1.3*), 363 (*98.7*) [1.32] ^b^	358	550 ^c^ 542 ^b^	0.42 ^c^	1.2 (*0.2*), 60 (*2.3*) 639 (*97.5*) [1.07] ^d^	624	560 ^c^	0.13 ^c^

a*:* from ref. [32]; b: from ref. [19]; c: from ref. [24]; d: from ref. [56]. The full decay data are given in the ESI (Figures S50 and S51).

**Table 3:** Selected crystallographic data of Re(I) complexes **Re-Tapy**, **Re-T-Tapy-Me**, **Re-T-Tapy-Et**, and **Re-T-Pyta_(1,2,3)_-Et**.

	Re-Tapy	Re-T-Tapy-Me	Re-T-Tapy-Et	Re-T-Pyta_(1,2,3)_-Et
Empirical formula	C_16_H_10_ClN_4_O_3_Re • C_2_H_6_OS	C_17_H_12_ClN_4_O_3_Re • CH_2_Cl_2_	C_18_H_14_ClN_4_O_3_Re	C_18_H_14_ClN_4_O_3_Re
Formula weight	606.06	626.88	555.98	555.98
Crystal system	Monoclinic	Triclinic	Monoclinic	Triclinic
Space group	*P21*	$P\bar{1}$	*P21*/*c*	$P\bar{1}$
Unit cell dimensions				
*a* (Å)	6.8027(3)	7.9671(4)	8.3767(4)	9.3125(4)
*b* (Å)	14.4708(6)	11.1386(6)	7.3859(3)	12.2753(5)
*c* (Å)	11.1451(5)	12.6625(7)	30.3253(13)	16.5599(6)
*α* (°)	90	89.6778(16)	90	88.7470(10)
*β* (°)	107.0980(15)	79.3892(14)	95.9593(17)	88.4940(10)
*γ* (°)	90	75.9949(14)	90	79.6560(10)
Volume (Å^3^)	1048.64(8)	1070.76(10)	1866.07(14)	1861.33(13)
Z	2	2	4	4
Density (calculated) (Mg/m^3^)	1.919	1.944	1.979	1.984
Crystal size (mm^3^)	0.450 × 0.120 × 0.040	0.200 × 0.160 × 0.040	0.180 × 0.160 × 0.100	0.080 × 0.080 × 0.050
Reflections collected	24,760	37,881	47,266	60,807
Independent reflections	4394 R_int_ = 0.0414	5431 R_int_ = 0.0342	3406 R_int_ = 0.0568	9383 R_int_ = 0.0751
Restraints/parameters	115/281	0/263	0/245	0/489
Final R1 index I > 2σ(I)	0.0257	0.0151	0.0267	0.0317
wR2 (all data)	0.0652	0.0354	0.0663	0.0547
Largest diff. peak and hole (e Å^−3^)	0.461 and −1.221	0.794 and −0.748	0.666 and −1.533	0.880 and −0.988
CCDC	2,454,799	2,454,800	2,454,801	2,454,802

## Data Availability

Dataset available on request from the authors.

## Supplementary Materials

The following supporting information can be downloaded at: https://www.mdpi.com/article/10.3390/molecules30132776/s1.
