# Supplementary material for: Influence of Ligand Isomerism on the Photophysical Properties of AIPE-Active Rhenium(I) Complexes: Investigations with a 2-(1,2,3-Triazol-1-yl)pyridine (Tapy)-Based Complex and Its Triazolylidene Derivatives
Source: Molecules. 2025 Jun 27;30(13):2776. doi: 10.3390/molecules30132776 (PMC12251393; doi:10.3390/molecules30132776)

checkCIF/PLATON report

Structure factors have been supplied for datablock(s) Re_T_Pyta_1_2_3_Et, Re_T_Tapy_Et, Re_T_Tapy_Me, Re_Tapy

THIS REPORT IS FOR GUIDANCE ONLY. IF USED AS PART OF A REVIEW PROCEDURE FOR PUBLICATION, IT SHOULD NOT REPLACE THE EXPERTISE OF AN EXPERIENCED CRYSTALLOGRAPHIC REFEREE.

No syntax errors found.   CIF dictionary   Interpreting this report

Datablock: Re_Tapy

Bond precision: C-C = 0.0113 A   Wavelength=1.54178

Cell:	a=6.8027(3)	b=14.4708(6)	c=11.1451(5)
	alpha=90	beta=107.0980(15)	gamma=90
Temperature:	193 K		

	Calculated	Reported
Volume	1048.64(8)	1048.64(8)
Space group	P 21	P 21
Hall group	P 2yb	P 2yb
Moiety formula	C16 H10 Cl N4 O3 Re, C2 H6 O S	?
Sum formula	C18 H16 Cl N4 O4 Re S	C18 H16 Cl N4 O4 Re S
Mr	606.07	606.06
Dx,g cm-3	1.919	1.919
Z	2	2
Mu (mm-1)	13.722	13.722
F000	584.0	584.0
F000'	576.29	
h,k,lmax	8,18,14	8,18,14
Nref	4469[2326]	4394
Tmin,Tmax	0.173,0.578	0.465,0.754
Tmin'	0.002	

Correction method= # Reported T Limits: Tmin=0.465 Tmax=0.754
AbsCorr = MULTI-SCAN

Data completeness= 1.89/0.98   Theta(max)= 77.447

R(reflections)= 0.0257(4310)

wR2(reflections)= 0.0652(4394)

S = 1.061 Npar= 281

The following ALERTS were generated. Each ALERT has the format
test-name_ALERT_alert-type_alert-level.
Click on the hyperlinks for more details of the test.

[IMAGE] **Alert level C**

PLAT342_ALERT_3_C Low Bond Precision on C-C Bonds .... 0.01133 Ang.

[IMAGE] **Alert level G**

PLAT002_ALERT_2_G Number of Distance or Angle Restraints on AtSite 8 Note
PLAT003_ALERT_2_G Number of Uiso or U(i,j) Restrained non-H-Atoms 8 Report
PLAT168_ALERT_4_G The CIF-Embedded .res File Contains EXYZ Records 2 Report
PLAT171_ALERT_4_G The CIF-Embedded .res File Contains EADP Records 2 Report
PLAT175_ALERT_4_G The CIF-Embedded .res File Contains SAME Records 1 Report
PLAT177_ALERT_4_G The CIF-Embedded .res File Contains DELU Records 1 Report
PLAT178_ALERT_4_G The CIF-Embedded .res File Contains SIMU Records 1 Report
PLAT188_ALERT_3_G A Non-default SIMU Restraint Value has been used 0.0100 Report
PLAT302_ALERT_4_G Anion/Solvent/Minor-Residue Disorder (Resd 2) 100% Note
PLAT302_ALERT_4_G Anion/Solvent/Minor-Residue Disorder (Resd 3) 100% Note
PLAT304_ALERT_4_G Non-Integer Number of Atoms in ..... (Resd 2) 9.18 Check
PLAT304_ALERT_4_G Non-Integer Number of Atoms in ..... (Resd 3) 0.82 Check
PLAT432_ALERT_2_G Short Inter X...Y Contact S1' ..C2 . 3.19 Ang.
-x,1/2+y,-z = 2_555 Check
PLAT432_ALERT_2_G Short Inter X...Y Contact O4' ..C9 . 2.97 Ang.
x,y,-1+z = 1_554 Check
PLAT860_ALERT_3_G Number of Least-Squares Restraints .... 115 Note
PLAT912_ALERT_4_G Missing # of FCF Reflections Above STh/L= 0.600 9 Note
PLAT933_ALERT_2_G Number of HKL-OMIT Records in Embedded .res File 3 Note
5-12 1, 1 -2 0, 3 -3 10,
PLAT969_ALERT_5_G The 'Henn et al.' R-Factor-gap value .... 2.336 Note
Predicted wR2: Based on SigI**2 2.79 or SHELX Weight 6.15
PLAT978_ALERT_2_G Number C-C Bonds with Positive Residual Density. 1 Info

---

0 **ALERT level A** = Most likely a serious problem - resolve or explain
0 **ALERT level B** = A potentially serious problem, consider carefully
1 **ALERT level C** = Check. Ensure it is not caused by an omission or oversight
19 **ALERT level G** = General information/check it is not something unexpected

0 ALERT type 1 CIF construction/syntax error, inconsistent or missing data
6 ALERT type 2 Indicator that the structure model may be wrong or deficient
3 ALERT type 3 Indicator that the structure quality may be low
10 ALERT type 4 Improvement, methodology, query or suggestion
1 ALERT type 5 Informative message, check

Datablock: Re_T_Tapy_Me

Bond precision: C-C = 0.0033 A Wavelength=0.71073

Cell:	a=7.9671(4)	b=11.1386(6)	c=12.6625(7)
	alpha=89.6778(16)	beta=79.3892(14)	gamma=75.9949(14)
Temperature:	193 K		

	Calculated	Reported
Volume	1070.76(10)	1070.76(10)
Space group	P -1	P -1
Hall group	-P 1	-P 1
Moiety formula	C17 H12 Cl N4 O3 Re, C H2 Cl2	?
Sum formula	C18 H14 Cl3 N4 O3 Re	C18 H14 Cl3 N4 O3 Re
Mr	626.89	626.88
Dx,g cm-3	1.944	1.944
Z	2	2
Mu (mm-1)	6.075	6.075
F000	600.0	600.0
F000'	599.11	
h,k,lmax	10,15,17	10,14,17
Nref	5512	5431
Tmin,Tmax	0.324,0.784	0.522,0.746
Tmin'	0.285	

Correction method= # Reported T Limits: Tmin=0.522 Tmax=0.746
AbsCorr = MULTI-SCAN

Data completeness= 0.985 Theta(max)= 28.669

R(reflections)= 0.0151(5136) wR2(reflections)= 0.0354(5431)

S = 1.027 Npar= 263

The following ALERTS were generated. Each ALERT has the format
test-name_ALERT_alert-type_alert-level.
Click on the hyperlinks for more details of the test.

[IMAGE] **Alert level C**
PLAT244_ALERT_4_C Low 'Solvent' Ueq as Compared to Neighbors of C18 Check

---

[IMAGE] **Alert level G**
PLAT232_ALERT_2_G Hirshfeld Test Diff (M-X) Re1 --Cl1 . 7.0 s.u.
PLAT232_ALERT_2_G Hirshfeld Test Diff (M-X) Re1 --C1 . 8.8 s.u.
PLAT232_ALERT_2_G Hirshfeld Test Diff (M-X) Re1 --C2 . 8.1 s.u.
PLAT232_ALERT_2_G Hirshfeld Test Diff (M-X) Re1 --C3 . 6.6 s.u.

```
PLAT432_ALERT_2_G Short Inter X...Y Contact  O2       ..C10      .       2.94 Ang.
                                                    1-x,1-y,1-z  =      2_666 Check
PLAT910_ALERT_3_G Missing # of FCF Reflection(s) Below Theta(Min).          3 Note
                0  1  0,   0 -1  1,   0  0  1,
PLAT912_ALERT_4_G Missing # of FCF Reflections Above STh/L=  0.600         75 Note
PLAT933_ALERT_2_G Number of HKL-OMIT Records in Embedded .res File          2 Note
                0  1  0,   0 -1  1,
PLAT969_ALERT_5_G The 'Henn et al.' R-Factor-gap value ..........      1.606 Note
              Predicted wR2: Based on SigI**2  2.21 or SHELX Weight  3.45
PLAT978_ALERT_2_G Number C-C Bonds with Positive Residual Density.          7 Info
```

```
   0 ALERT level A = Most likely a serious problem - resolve or explain
   0 ALERT level B = A potentially serious problem, consider carefully
   1 ALERT level C = Check. Ensure it is not caused by an omission or oversight
  10 ALERT level G = General information/check it is not something unexpected

   0 ALERT type 1 CIF construction/syntax error, inconsistent or missing data
   7 ALERT type 2 Indicator that the structure model may be wrong or deficient
   1 ALERT type 3 Indicator that the structure quality may be low
   2 ALERT type 4 Improvement, methodology, query or suggestion
   1 ALERT type 5 Informative message, check
```

Datablock: Re_T_Tapy_Et

Bond precision: C-C = 0.0057 A Wavelength=1.54178

Cell:	a=8.3767(4)	b=7.3859(3)	c=30.3253(13)
	alpha=90	beta=95.9593(17)	gamma=90
Temperature:	193 K		

	Calculated	Reported
Volume	1866.07(14)	1866.07(14)
Space group	P 21/c	P 21/c
Hall group	-P 2ybc	-P 2ybc
Moiety formula	C18 H14 Cl N4 O3 Re	?
Sum formula	C18 H14 Cl N4 O3 Re	C18 H14 Cl N4 O3 Re
Mr	555.99	555.98
Dx,g cm-3	1.979	1.979
Z	4	4
Mu (mm-1)	14.291	14.291
F000	1064.0	1064.0
F000'	1047.03	
h,k,lmax	10,8,36	10,8,36
Nref	3409	3406
Tmin,Tmax	0.145,0.240	0.399,0.753
Tmin'	0.040	

Correction method= # Reported T Limits: Tmin=0.399 Tmax=0.753
AbsCorr = MULTI-SCAN

Data completeness= 0.999 Theta(max)= 68.258

R(reflections)= 0.0267(3321) wR2(reflections)= 0.0663(3406)

S = 1.131 Npar= 245

The following ALERTS were generated. Each ALERT has the format
test-name_ALERT_alert-type_alert-level.
Click on the hyperlinks for more details of the test.

[IMAGE] **Alert level C**

```
PLAT972_ALERT_2_C Check Calcd Resid. Dens.  0.72Ang From Re1           -1.55 eA-3
```

[IMAGE] **Alert level G**

```
PLAT230_ALERT_2_G Hirshfeld Test Diff for    O3       --C3       .        6.7 s.u.
PLAT232_ALERT_2_G Hirshfeld Test Diff (M-X)  Re1      --Cl1      .        9.0 s.u.
PLAT232_ALERT_2_G Hirshfeld Test Diff (M-X)  Re1      --C1       .        7.1 s.u.
PLAT232_ALERT_2_G Hirshfeld Test Diff (M-X)  Re1      --C3       .        8.5 s.u.
PLAT910_ALERT_3_G Missing # of FCF Reflection(s) Below Theta(Min).          1 Note
                0  0  2,
PLAT912_ALERT_4_G Missing # of FCF Reflections Above STh/L=  0.600          1 Note
PLAT933_ALERT_2_G Number of HKL-OMIT Records in Embedded .res File          2 Note
                0  0  2,   1  1  0,
PLAT969_ALERT_5_G The 'Henn et al.' R-Factor-gap value ..........       2.718 Note
              Predicted wR2: Based on SigI**2  2.44 or SHELX Weight  5.86
PLAT978_ALERT_2_G Number C-C Bonds with Positive Residual Density.          0 Info
```

---

0 **ALERT level A** = Most likely a serious problem - resolve or explain
0 **ALERT level B** = A potentially serious problem, consider carefully
1 **ALERT level C** = Check. Ensure it is not caused by an omission or oversight
9 **ALERT level G** = General information/check it is not something unexpected

0 ALERT type 1 CIF construction/syntax error, inconsistent or missing data
7 ALERT type 2 Indicator that the structure model may be wrong or deficient
1 ALERT type 3 Indicator that the structure quality may be low
1 ALERT type 4 Improvement, methodology, query or suggestion
1 ALERT type 5 Informative message, check

Datablock: Re_T_Pyta_1_2_3_Et

Bond precision: C-C = 0.0072 A Wavelength=0.71073

Cell:	a=9.3125(4)	b=12.2753(5)	c=16.5599(6)
	alpha=88.747(1)	beta=88.494(1)	gamma=79.656(1)
Temperature:	193 K		

	Calculated	Reported
Volume	1861.33(13)	1861.33(13)
Space group	P -1	P -1
Hall group	-P 1	-P 1
Moiety formula	C18 H14 Cl N4 O3 Re	?
Sum formula	C18 H14 Cl N4 O3 Re	C18 H14 Cl N4 O3 Re
Mr	555.99	555.98
Dx,g cm-3	1.984	1.984
Z	4	4
Mu (mm-1)	6.698	6.698
F000	1064.0	1064.0
F000'	1061.05	
h,k,lmax	12,16,22	12,16,22
Nref	9482	9383
Tmin,Tmax	0.591,0.715	0.644,0.746
Tmin'	0.579	

Correction method= # Reported T Limits: Tmin=0.644 Tmax=0.746
AbsCorr = MULTI-SCAN

Data completeness= 0.990 Theta(max)= 28.540

R(reflections)= 0.0317(6911) wR2(reflections)= 0.0547(9383)

S = 1.021 Npar= 489

The following ALERTS were generated. Each ALERT has the format
test-name_ALERT_alert-type_alert-level.
Click on the hyperlinks for more details of the test.

[IMAGE] **Alert level C**

```
PLAT911_ALERT_3_C Missing FCF Refl Between Thmin & STh/L=    0.600          5 Report
                1  0  0,   1  1  0,   0  0  2,   2  1  2,   2  3  2,
```

[IMAGE] **Alert level G**

```
PLAT154_ALERT_1_G The s.u.'s on the Cell Angles are Equal ..(Note)      0.001 Degree
PLAT230_ALERT_2_G Hirshfeld Test Diff for    O3       --C3       .        6.0 s.u.
PLAT232_ALERT_2_G Hirshfeld Test Diff (M-X)  Re1      --C3       .        7.0 s.u.
PLAT232_ALERT_2_G Hirshfeld Test Diff (M-X)  Re2      --C20      .        5.3 s.u.
PLAT910_ALERT_3_G Missing # of FCF Reflection(s) Below Theta(Min).          1 Note
                0  0  1,
PLAT912_ALERT_4_G Missing # of FCF Reflections Above STh/L=  0.600         92 Note
```

```
PLAT933_ALERT_2_G Number of HKL-OMIT Records in Embedded .res File          4 Note
                0  0  2,   0  0  1,   2  3  2,   2  1  2,
PLAT969_ALERT_5_G The 'Henn et al.' R-Factor-gap value ..........      1.536 Note
              Predicted wR2: Based on SigI**2  3.56 or SHELX Weight  5.35
PLAT978_ALERT_2_G Number C-C Bonds with Positive Residual Density.          2 Info
```

```
  0 ALERT level A = Most likely a serious problem - resolve or explain
  0 ALERT level B = A potentially serious problem, consider carefully
  1 ALERT level C = Check. Ensure it is not caused by an omission or oversight
  9 ALERT level G = General information/check it is not something unexpected

  1 ALERT type 1 CIF construction/syntax error, inconsistent or missing data
  5 ALERT type 2 Indicator that the structure model may be wrong or deficient
  2 ALERT type 3 Indicator that the structure quality may be low
  1 ALERT type 4 Improvement, methodology, query or suggestion
  1 ALERT type 5 Informative message, check
```

It is advisable to attempt to resolve as many as possible of the alerts in all categories. Often the minor alerts point to easily fixed oversights, errors and omissions in your CIF or refinement strategy, so attention to these fine details can be worthwhile. In order to resolve some of the more serious problems it may be necessary to carry out additional measurements or structure refinements. However, the purpose of your study may justify the reported deviations and the more serious of these should normally be commented upon in the discussion or experimental section of a paper or in the "special_details" fields of the CIF. checkCIF was carefully designed to identify outliers and unusual parameters, but every test has its limitations and alerts that are not important in a particular case may appear. Conversely, the absence of alerts does not guarantee there are no aspects of the results needing attention. It is up to the individual to critically assess their own results and, if necessary, seek expert advice.

Publication of your CIF in IUCr journals

A basic structural check has been run on your CIF. These basic checks will be run on all CIFs submitted for publication in IUCr journals (*Acta Crystallographica*, *Journal of Applied Crystallography*, *Journal of Synchrotron Radiation*); however, if you intend to submit to *Acta Crystallographica Section C* or *E* or *IUCrData*, you should make sure that full publication checks are run on the final version of your CIF prior to submission.

Publication of your CIF in other journals

Please refer to the *Notes for Authors* of the relevant journal for any special instructions relating to CIF submission.

PLATON version of 02/02/2025; check.def file version of 02/02/2025

Datablock Re_Tapy - ellipsoid plot

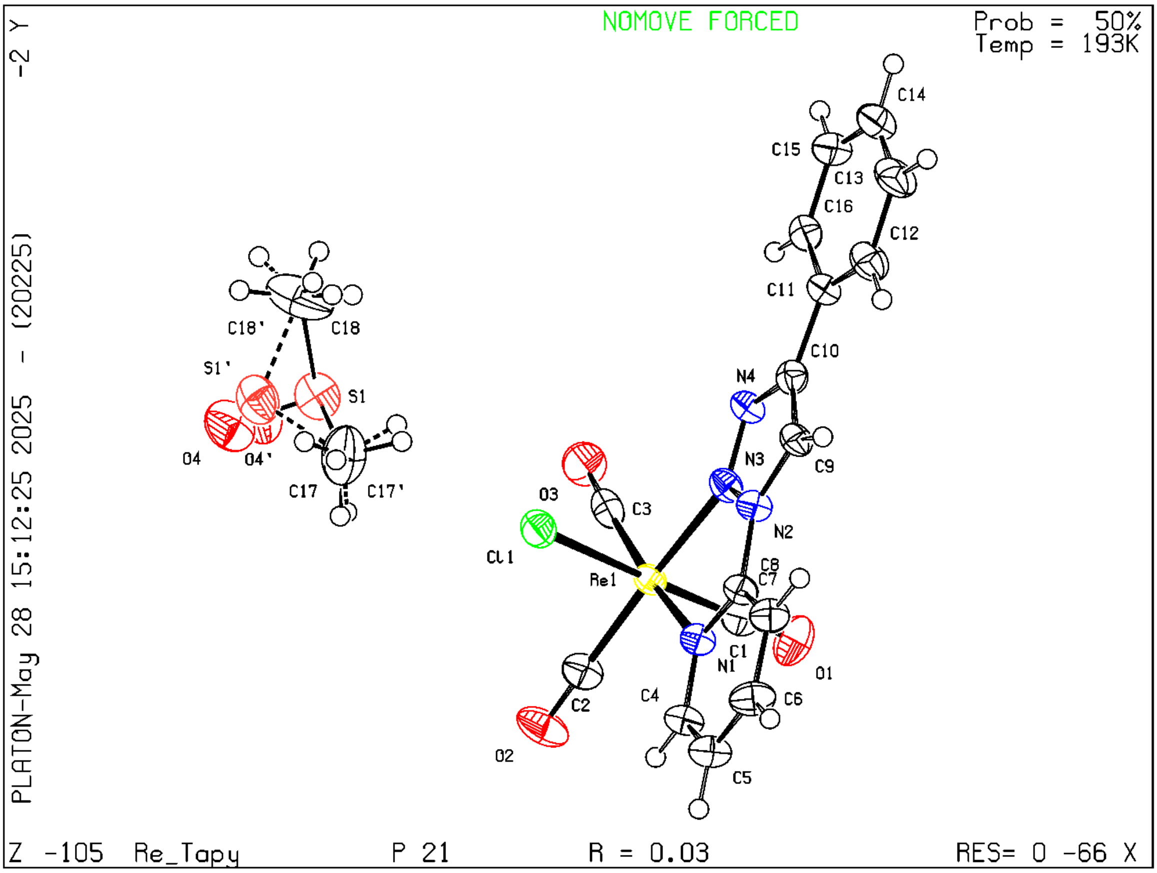

Datablock Re_T_Tapy_Me - ellipsoid plot

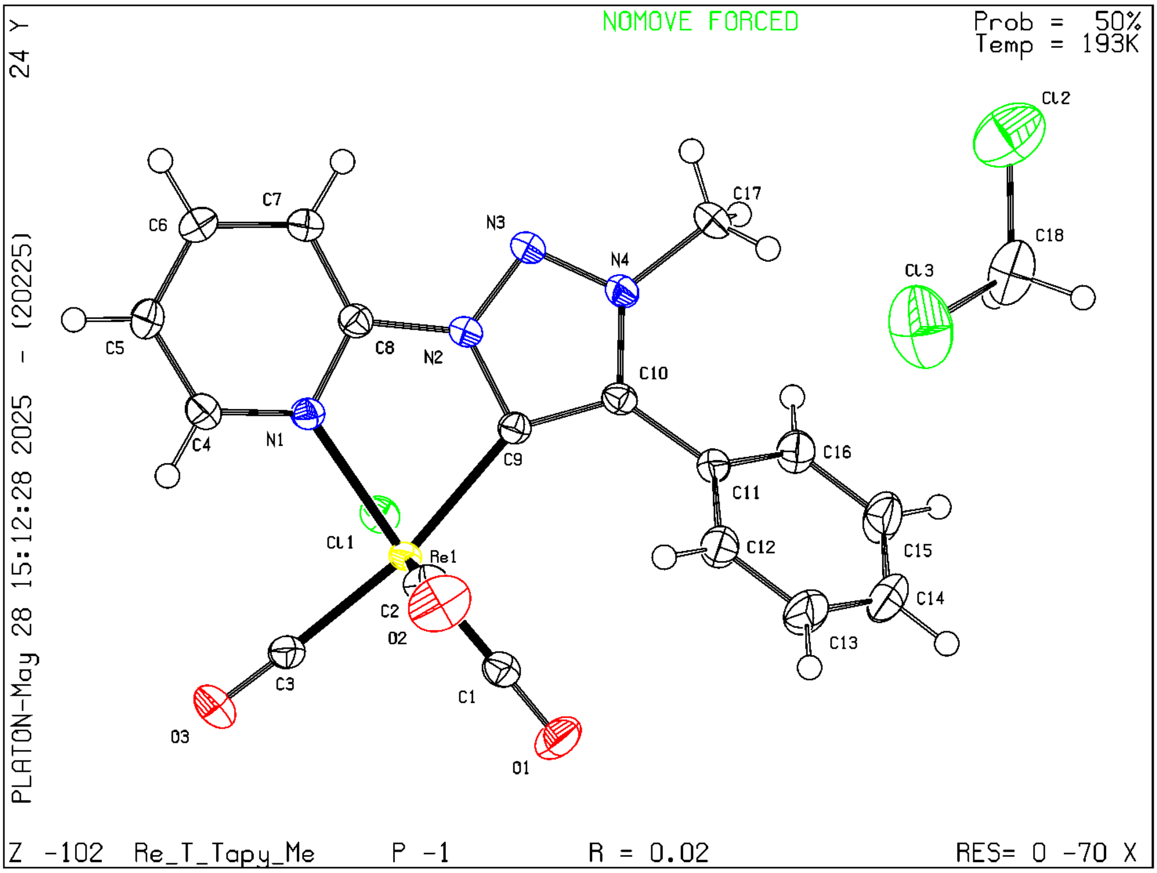

Datablock Re_T_Tapy_Et - ellipsoid plot

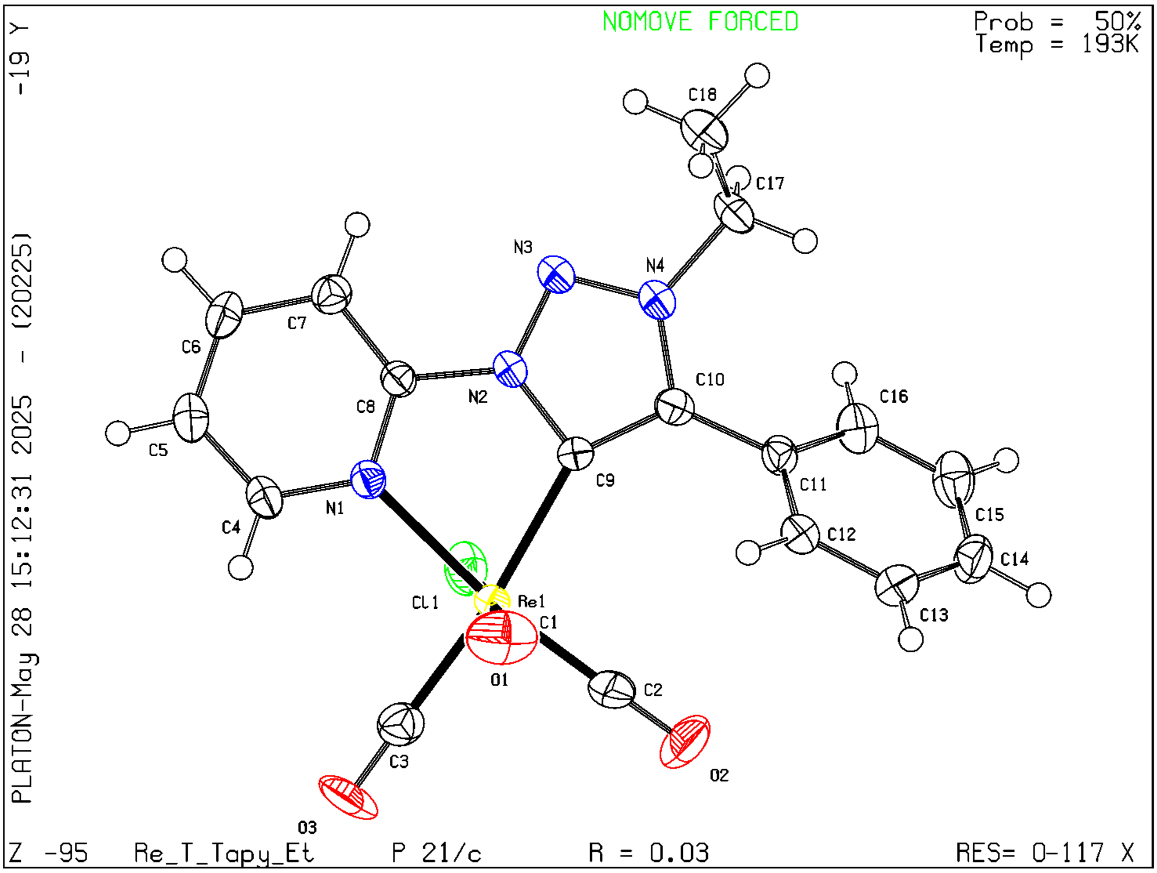

Datablock Re_T_Pyta_1_2_3_Et - ellipsoid plot

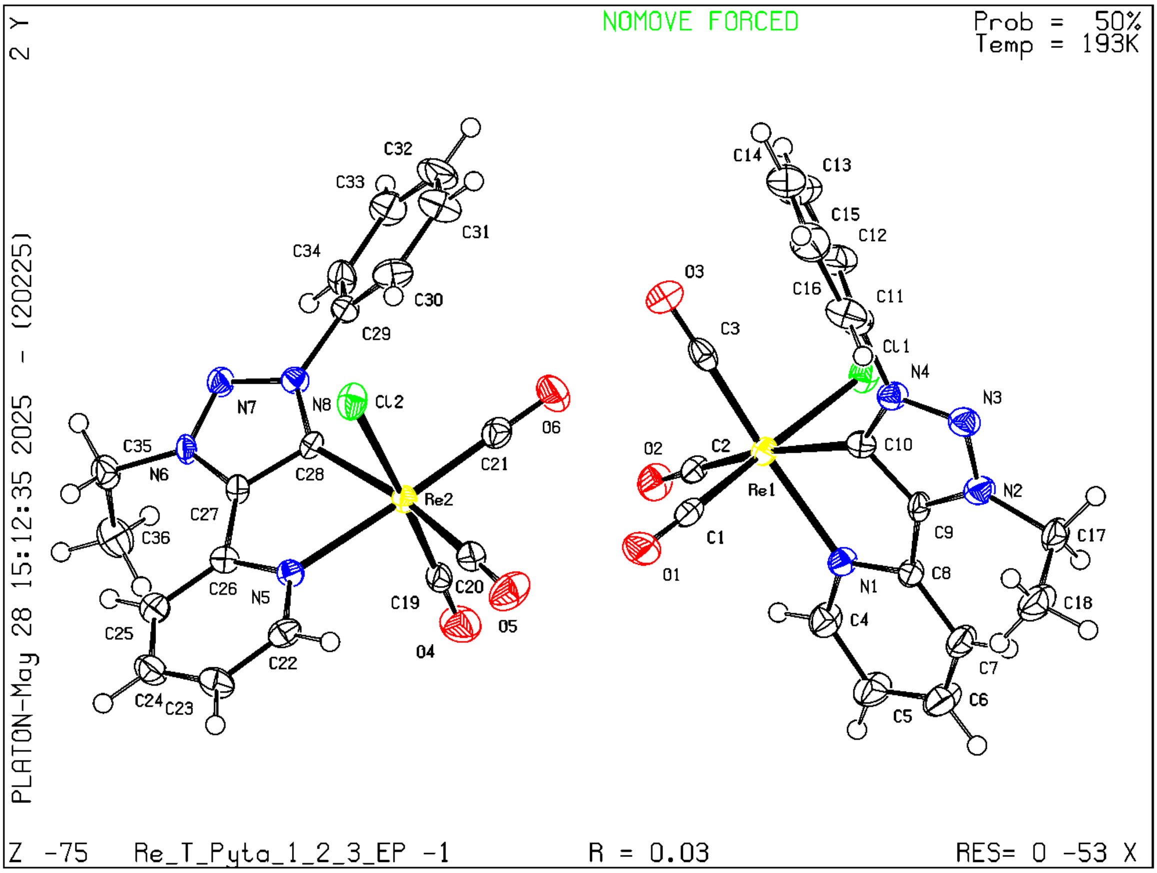

Supplement: Supplementary file 1 [file molecules-30-02776-s001.zip › checkcif_global_CCDC.pdf]
