# Supplementary material for: Influence of Ligand Isomerism on the Photophysical Properties of AIPE-Active Rhenium(I) Complexes: Investigations with a 2-(1,2,3-Triazol-1-yl)pyridine (Tapy)-Based Complex and Its Triazolylidene Derivatives
Source: Molecules. 2025 Jun 27;30(13):2776. doi: 10.3390/molecules30132776 (PMC12251393; doi:10.3390/molecules30132776)
Supplement: Supplementary file 1 [file molecules-30-02776-s001.zip › AA1_ESI (Revised).pdf]

Electronic Supplementary Information

Influence of Ligand Isomerism on the Photophysical Properties of AIPE-active Rhenium(I) Complexes. Investigations with a 2-(1,2,3-triazol-1-yl)pyridine (Tapy)-based Complex and its Triazolylidene Derivatives

Abanoub Mosaad Abdallah, Mariusz Wolff, Nadine Leygue, Maëlle Deleuzière, Nathalie Saffon-Merceron, Charles-Louis Serpentini, Eric Benoist and Suzanne Fery-Forgues

Synthesis and characterization

Scheme S1. Synthesis of tricarbonylrhenium(I) complex **Re-T-Pyta$_{(1,2,3)}$-Et** 3
Figure S1. ^{1}H NMR spectrum of compound **1** in $CDCl_3$ 3
Figure S2. ^{13}C Jmod NMR and HSQC spectra of compound **1** in $CDCl_3$ 4
Figure S3. HRMS spectrum of compound **1**.. 5
Figure S4. ^{1}H NMR and ^{13}C Jmod NMR spectrra of **L-T-Pyta$_{(1,2,3)}$-Et** in $CDCl_3$ 6
Figure S5. HSQC spectrum of **L-T-Pyta$_{(1,2,3)}$-Et** in $CDCl_3$ 7
Figure S6. HRMS spectrum of **L-T-Pyta$_{(1,2,3)}$-Et** 8
Figure S7. ^{1}H NMR and ^{13}C Jmod NMR spectra of **Re-T-Pyta$_{(1,2,3)}$-Et** in $CDCl_3$ 9
Figure S8. HSQC spectrum of **Re-T-Pyta$_{(1,2,3)}$-Et** in $CDCl_3$ 10
Figure S9. FT-IR spectrum of **Re-T-Pyta$_{(1,2,3)}$Et** 10
Figure S10. HRMS spectrum of **Re-T-Pyta$_{(1,2,3)}$-Et** (data) 11
Figure S11. HRMS spectrum of **Re-T-Pyta$_{(1,2,3)}$-Et** (calculations) 12
Figure S12. ^{1}H NMR and ^{13}C Jmod NMR spectra of **L-T-Tapy-Me** in $CDCl_3$ 13
Figure S13. HSQC spectrum of **L-T-Tapy-Me** in $CDCl_3$ 14
Figure S14. High resolution mass spectrum of **L-T-Tapy-Me** 15
Figure S15. ^{1}H NMR and ^{13}C Jmod NMR spectra of **L-T-Tapy-Et** in $CDCl_3$ 16
Figure S16. HSQC spectra of **L-T-Tapy-Et** in $CDCl_3$ 17
Figure S17. ^{1}H NMR spectrum of **Re-T-Tapy-Me** in $CDCl_3$ 17
Figure S18. ^{13}C Jmod NMR and HSQC spectra of **Re-T-Tapy-Me** in $CDCl_3$ 18
Figure S19. High resolution mass spectrum of **Re-T-Tapy-Me** 19
Figure S20. FT-IR spectrum of **Re-T-Tapy-Me** 20
Figure S21. ^{1}H NMR spectrum of **Re-T-Tapy-Et** in $CDCl_3$ 20
Figure S22. ^{13}C Jmod NMR HSQC spectrum of **Re-T-Tapy-Et** in $CDCl_3$............................. 21
Figure S23. FT-IR spectrum of **Re-T-Tapy-Et** 22
Figure S24. High resolution mass spectrum of **Re-T-Tapy-Et** 22
Figure S25. High resolution mass spectrum of **Re-T-Tapy-Et** 23

Crystallography

Octahedral distortion parameters of **Re-Tapy**, **Re-T-Tapy-Me** and **Re-T-Tapy-Et** 24
Table S1. Octahedral distortion parameters of **Re-Tapy**, **Re-T-Tapy-Me** and **Re-T-Tapy-Et** 24
Intermolecular interactions in crystal structures 24
Table S2. Short contacts detected in structures of the four complexes 24
Table S3. C–H···π interactions detected in structures of the three complexes 25
Table S4. π···π interactions in structures of **Re-Tapy**, **Re-T-Tapy-Et** and **Re-T-Pyta$_{(1,2,3)}$-Et** ... 25
Figure S26. Intermolecular interactions stabilizing the structure of **Re-Tapy** 25
Figure S27. Weak intramolecular interactions stabilizing the structure of **Re-Tapy** 26
Figure S28. Intermolecular interactions stabilizing the structure of **Re-T-Tapy-Me** 26
Figure S29. Intermolecular interactions stabilizing the structure of **Re-T-Tapy-Et** 27
Hirshfeld surface analysis 27
Figure S30. HS plotted over d_{norm}, d_{i}, d_{e} of **Re-Tapy**, **Re-T-Tapy-Me**, and **Re-T-Tapy-Et** 28
Figure S31. HS plotted over curvedness and shape index of **Re-Tapy** and **Re-T-Tapy-Et** 28
Analysis of the HS of **Re-Tapy** 29
Figure S32. 2D-Fingerprint plots for interactions in crystal packing of **Re-Tapy** 30
Analysis of the HS of **Re-T-Tapy-Me** 30
Figure S33. 2D-fingerprint plots for interactions in crystal packing of **Re-Tapy** 31

Analysis of the HS of **Re-T-Tapy-Et** …………………………………………………………. 31
Figure S34. 2D-fingerprint plots for interactions in crystal packing of **Re-Tapy** ………………. 32
Figure S35. Molecular arrangement of **Re-T-Pyta$_{(1,2,3)}$-Et** ……………………………………... 33

DFT and TD-DFT Calculations

Table S5. Selected calculated bond lengths and angles in the S_0, S_1 and T_1 states for **Re-Tapy** …... 34
Table S6. Selected calculated bond lengths and angles in the S_0, S_1 and T_1 states for **Re-T-Tapy-Me** ……………………………………………………………………………………. 35
Table S7. Calculated dihedral angle for the S_0, S_1 and T_1 states for **Re-Tapy** and **Re-T-Tapy-Me** 36
Table S8. Frontier molecular orbital composition and energy levels for **Re-Tapy** ……………….. 36
Table S9. Frontier molecular orbital composition and energy levels for **Re-T-Tapy-Me** ……….. 36
Table S10. Main electronic transitions for **Re-Tapy** ……………………………………………... 37
Table S11. Main electronic transitions for **Re-T-Tapy-Me** ……………………………………… 37
Table S12. Excitation energies and oscillator strengths calculated on the optimized geometry of the S_1 state of complexes **Re-Tapy** and **Re-T-Tapy-Me** ………………………………………. 38
Table S13. Phosphorescence emission energies of **Re-Tapy** and **Re-T-Tapy-Me** ……………… 38
Table S14. Natural populations of 5d orbitals of the central atom in **Re-Tapy** and **Re-T-Tapy-Me** …………………………………………………………………………………………… 38
Table S15. Atomic charges from Natural Population Analysis for **Re-Tapy** and **Re-T-Tapy-Me** . 39
Table S16. HOMO-LUMO gap, absolute electronegativity, absolute hardness, dipole moment, electrophilicity index and global softness of **Re-Tapy** and **Re-T-Tapy-Me** ……………………… 39
Figure S36. Isodensity plots of the frontier MO of **Re-Tapy** ……………………………………. 40
Figure S37. Isodensity plots of the frontier MO of **Re-T-Tapy-Me** ……………………………... 41
Figure S38. Spin density distribution for T_1 of **Re-Tapy** and **Re-T-Tapy-Me** …………………… 42
Figure S39. Molecular Electrostatic Potential (MEP) of **Re-Tapy** and **Re-T-Tapy-Me** ………… 42
Figure S40. The natural transition orbitals (NTOs) of transitions $S_0 \rightarrow S_1$, $S_0 \rightarrow S_2$, $S_0 \rightarrow S_{12}$, $S_0 \rightarrow S_{13}$, $S_0 \rightarrow S_{16}$ and $S_0 \rightarrow S_{18}$ in **Re-Tapy** ……………………………………………………....... 43
Figure S41. The natural transition orbitals (NTOs) of transition $S_0 \rightarrow T_1$ in **Re-Tapy** …................. 43
Figure S42. The natural transition orbitals (NTOs) of transitions $S_0 \rightarrow S_1$, $S_0 \rightarrow S_2$, $S_0 \rightarrow S_{17}$ and $S_0 \rightarrow S_{19}$ in **Re-T-Tapy-Me** ……………………………………………………………………….. 44
Figure S43. The natural transition orbitals (NTOs) of transition $S_0 \rightarrow T_1$ in **Re-T-Tapy-Me** ……... 44
Figure S44. Exp. and simulated FT-IR and UV-vis absorption spectra of **Re-Tapy** in DCM 45
Figure S45. Exp. and simulated FT-IR and UV-vis absorption spectra of **Re-T-Tapy-Me** in DCM 45

Spectroscopy

Figure S46. Emission decays of the four complexes in dichloromethane ………………………… 46
Figure S47. Solid-state emission spectra of **Re-T-Tapy-Me** and **Re-T-Tapy-Et** ………………... 47
Figure S48. Solid-state emission spectra of **Re-T-Pyta$_{(1,2,3)}$-Me** and **Re-T-Pyta$_{(1,2,3)}$-Et** ………... 47
Figure S49. Absorption spectra of **Re-Tapy** and **Re-T-Tapy-Me** in water/acetonitrile mixtures . 47
Figure S50. Emission decays of the four complexes in the microcrystalline state ……………… 48
Figure S51. Emission decays of **Re-Tapy** and **Re-T-Tapy-Me** suspensions in water/acetonitrile 48
Figure S52. TEM images of the suspension of **Re-Tapy** in water/acetonitrile 80:20 v/v ……….. 49
Figure S53. TEM images of the suspension of **Re-Tapy** in water/acetonitrile 95:5 v/v ………….. 49
Figure S54. TEM images of the suspension of **Re-T-Tapy-Me** in water/acetonitrile 80:20 v/v … 50
Figure S55. TEM images of the suspension of **Re-T-Tapy-Me** in water/acetonitrile 95:5 v/v …... 51
Table S17. Dynamic light scattering (DLS) results for suspensions of **Re-Tapy** and **Re-T-Tapy** .. 51

Cartesian coordinates

Table S18. Cartesian coordinates of **Re-Tapy** in S_0 (in dichloromethane) ……………………… 52
Table S19. Cartesian coordinates of **Re-Tapy** in S_1 (in dichloromethane) ……………………... 53
Table S20. Cartesian coordinates of **Re-Tapy** in T_1 (in dichloromethane) ……………………… 54
Table S21. Cartesian coordinates of **Re-T-Tapy-Me** in S_0 (in dichloromethane) ……….……... 55
Table S22. Cartesian coordinates of **Re-T-Tapy-Me** in S_1 (in dichloromethane) ……………..... 56
Table S23. Cartesian coordinates of **Re-T-Tapy-Me** in T_1 (in dichloromethane) ……..……... 57

Synthesis and characterization

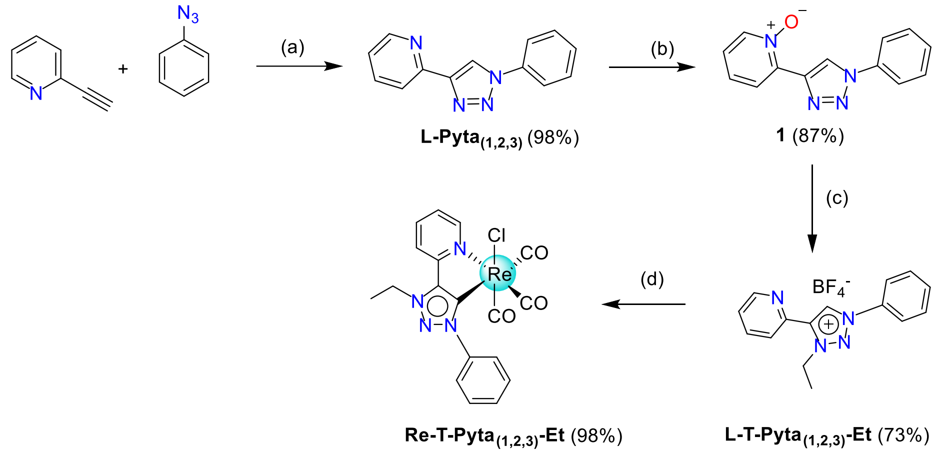

Scheme S1. Synthesis of tricarbonylrhenium(I) complex **Re-T-Pyta$_{(1,2,3)}$-Et**. Conditions: (a) $CuSO_4.5H_2O$, Na Ascorbate, *tert*-butanol/H_2O; (b) *m*-chloro-perbenzoic acic (*m*-CPBA), $CHCl_3$, reflux, 30 min; (c) (i) Et_3OBF_4, DCM, RT, 4 days; (ii) [$Mo(CO)_6$], EtOH, reflux, 1h; (d) [$Re(CO)_5Cl$], NEt_3 Toluene, reflux, 3 days.

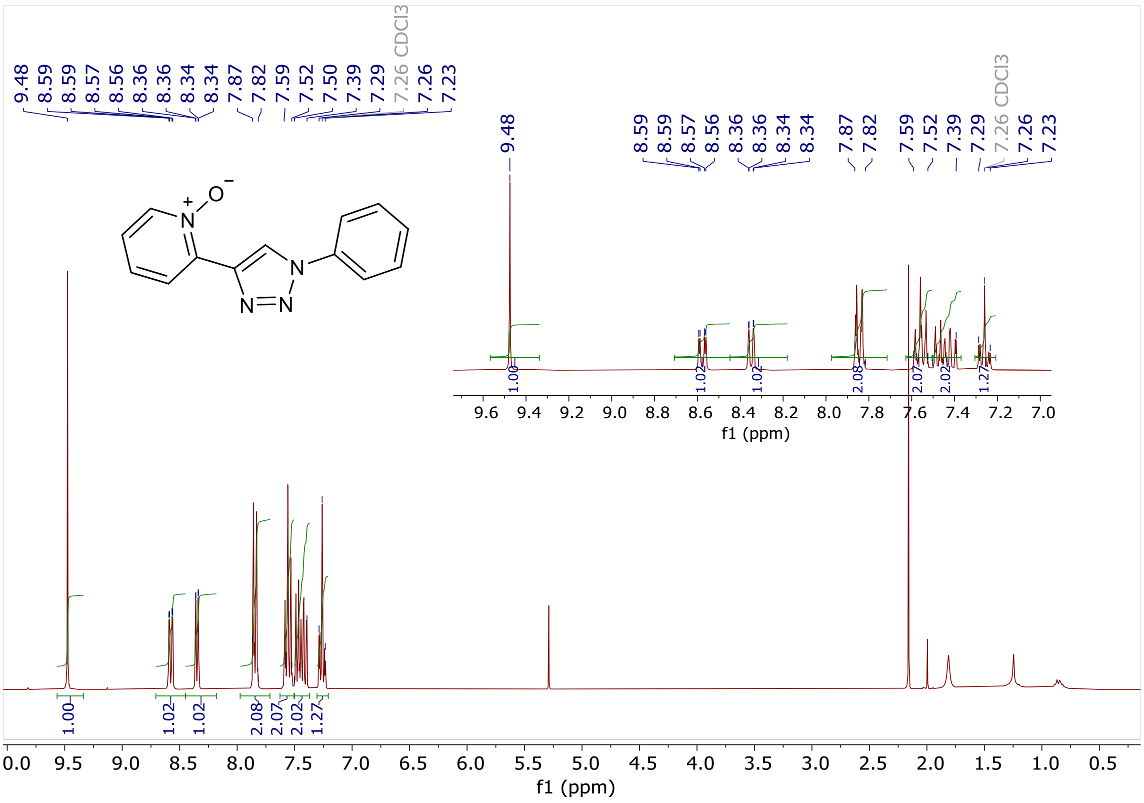

Figure S1. ^{1}H NMR spectrum of compound **1** in $CDCl_3$.

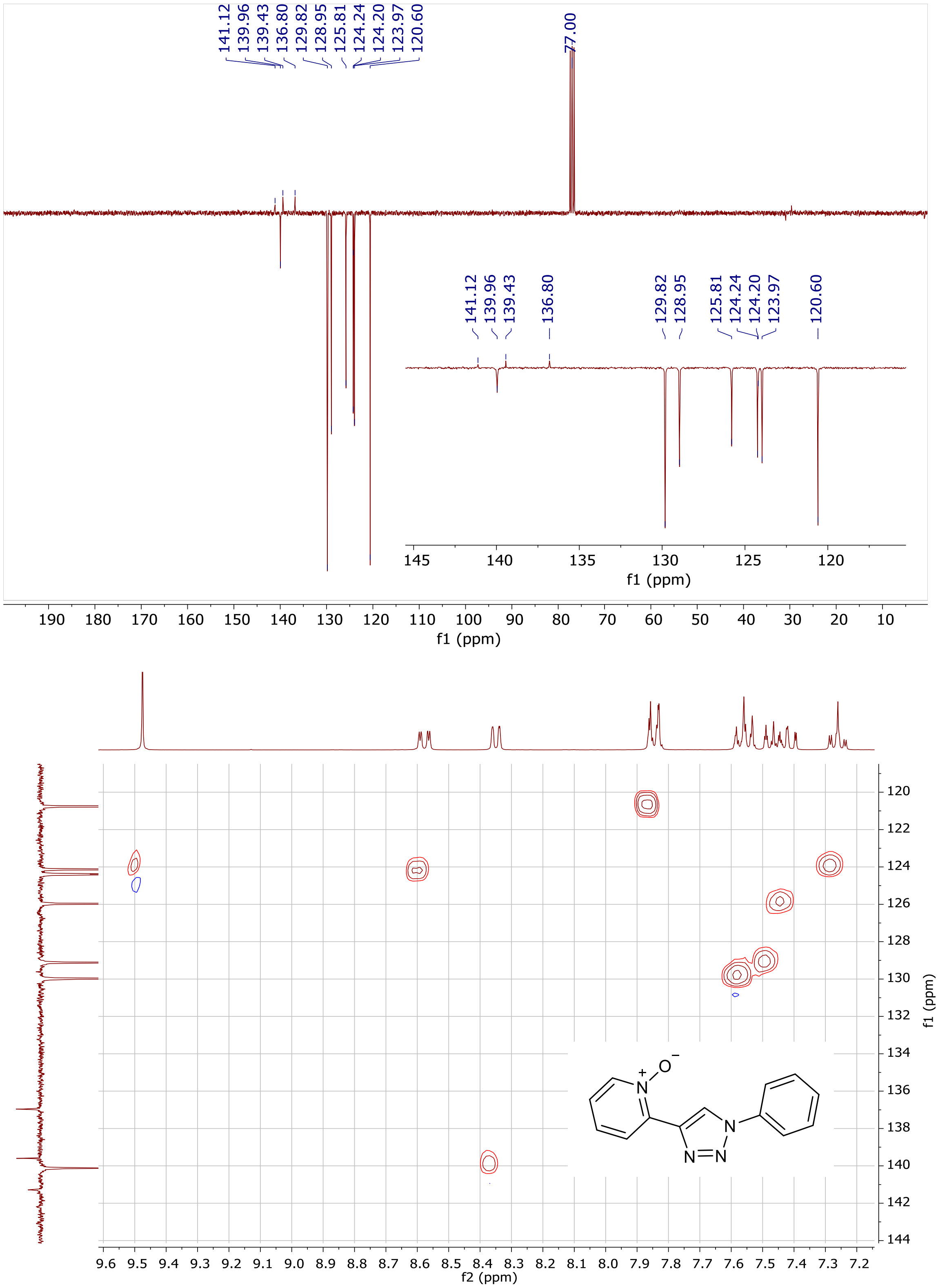

Figure S2. ^{13}C Jmod NMR (top) and HSQC (bottom) spectra of compound **1** in $CDCl_3$.

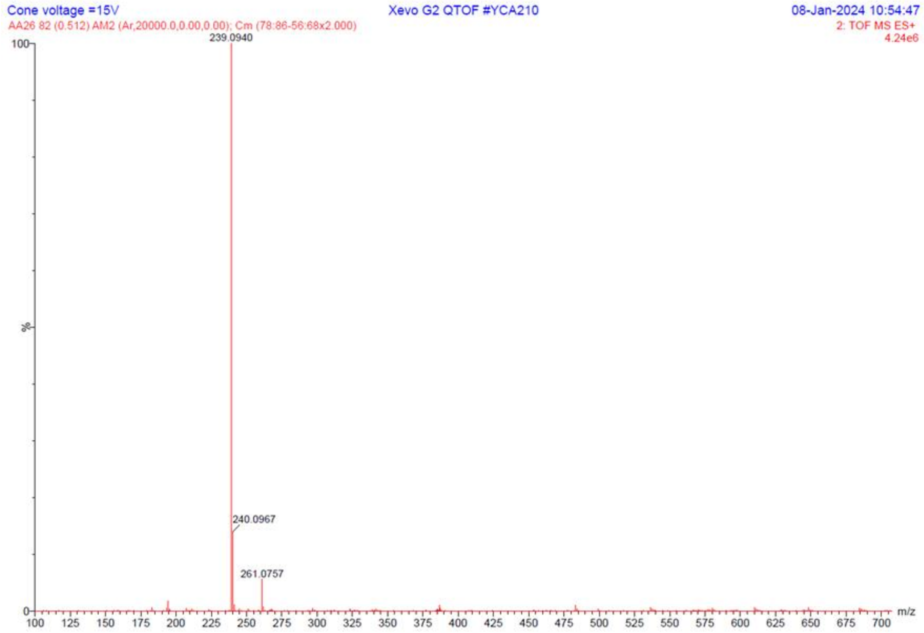

Elemental Composition Report

Single Mass Analysis
Tolerance = 3.0 PPM / DBE: min = -5.0, max = 100.0
Element prediction: Off
Number of isotope peaks used for i-FIT = 3

Monoisotopic Mass, Even Electron Ions
328 formula(e) evaluated with 1 results within limits (up to 50 closest results for each mass)
Elements Used:
C: 0-80 H: 0-100 N: 0-10 O: 0-10

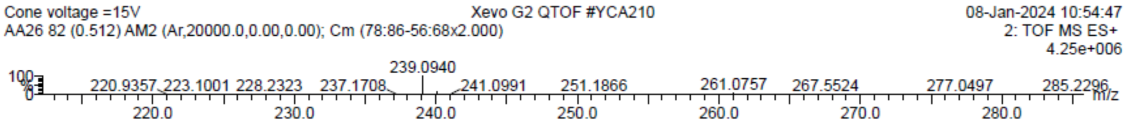

Minimum:				-5.0				
Maximum:		3.0	3.0	100.0				
Mass	Calc. Mass	mDa	PPM	DBE	i-FIT	Norm	Conf(%)	Formula
239.0940	239.0933	0.7	2.9	10.5	517.6	n/a	n/a	C13 H11 N4 O

Figure S3. High resolution mass spectrum of compound **1**.

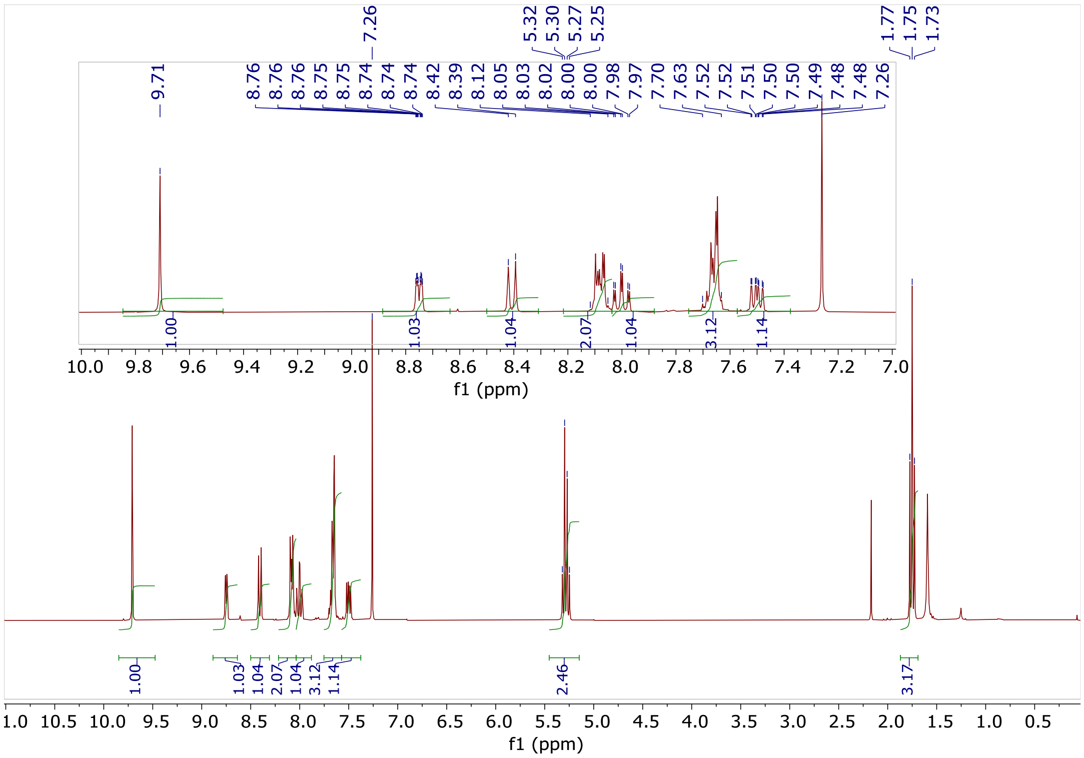

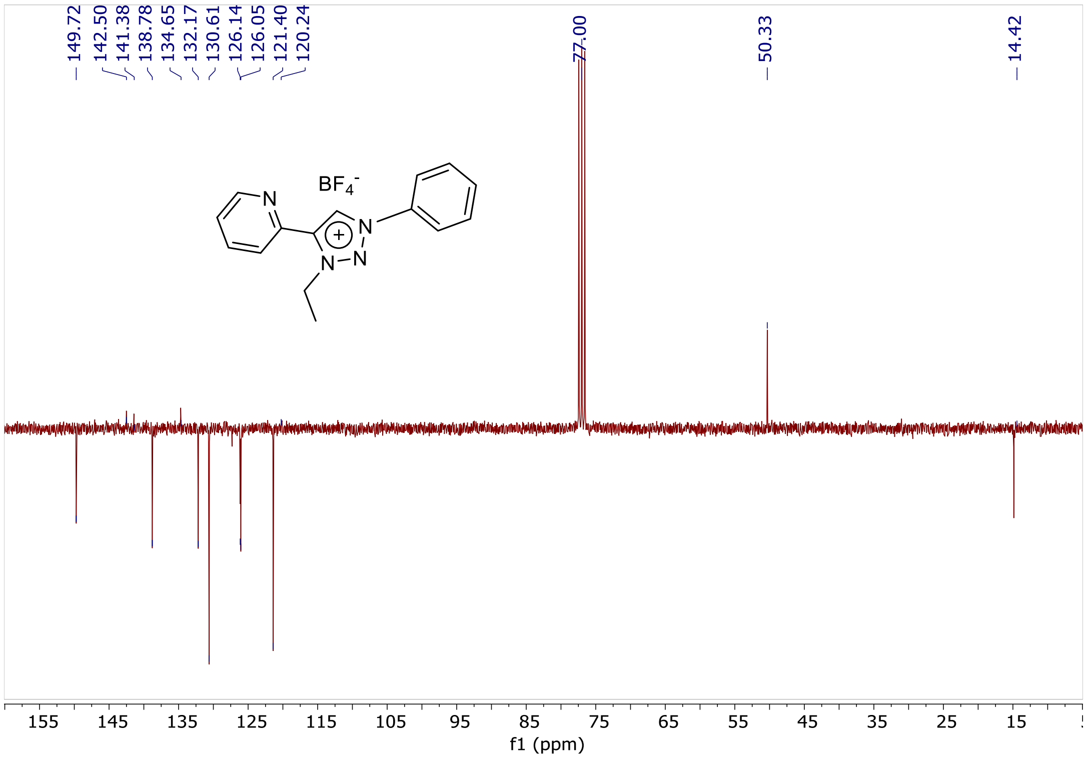

Figure S4. ^{1}H NMR (top) and ^{13}C Jmod NMR (bottom) spectra of **L-T-Pyta$_{(1,2,3)}$-Et** in $CDCl_3$.

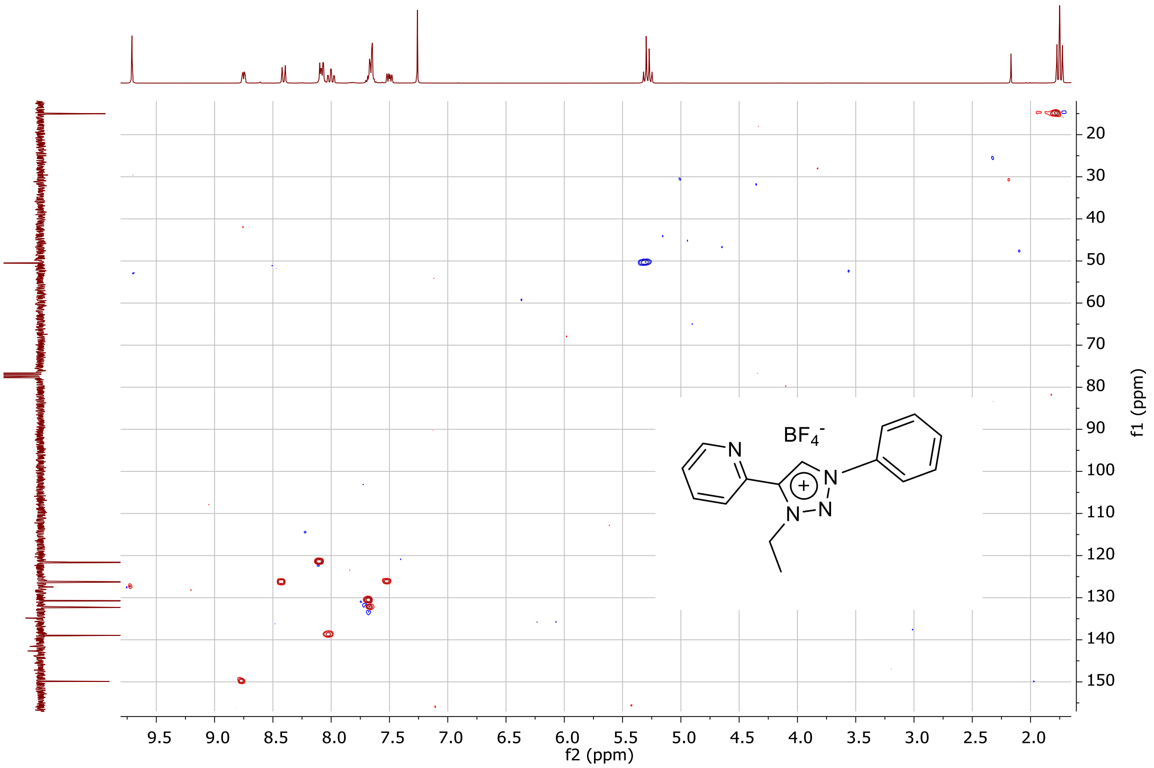

Figure S5. HSQC spectrum of **L-T-Pyta$_{(1,2,3)}$-Et** in $CDCl_3$.

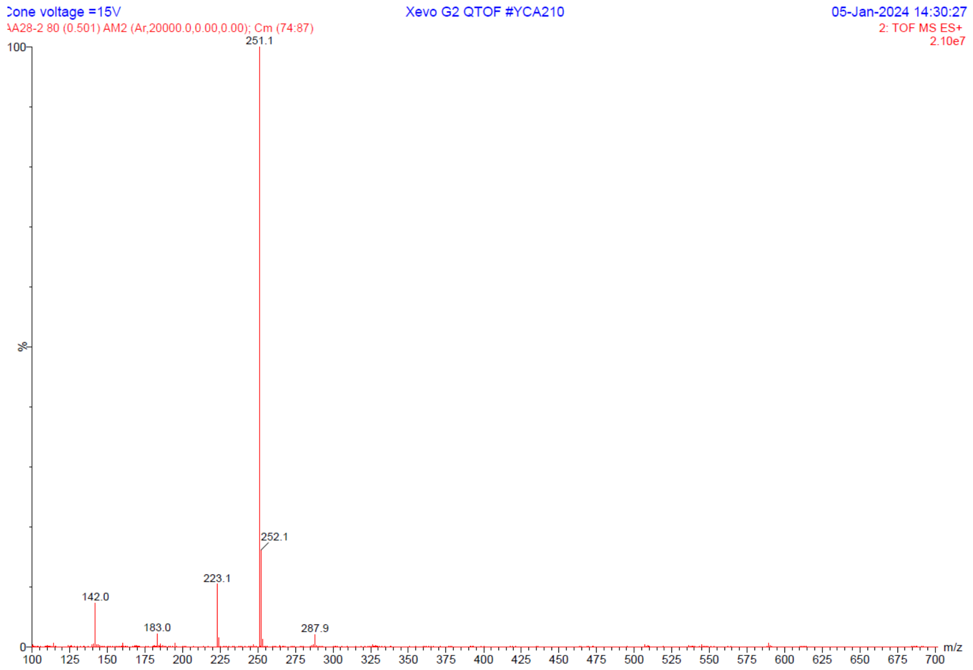

Elemental Composition Report

Single Mass Analysis

Tolerance = 3.0 PPM / DBE: min = -5.0, max = 100.0
Element prediction: Off
Number of isotope peaks used for i-FIT = 3

Monoisotopic Mass, Even Electron Ions
352 formula(e) evaluated with 1 results within limits (up to 50 closest results for each mass)
Elements Used:
C: 0-80 H: 0-100 N: 0-10 O: 0-10

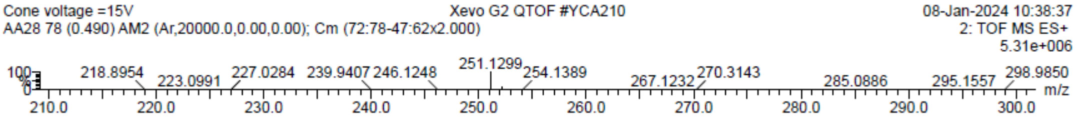

Minimum:				-5.0				
Maximum:		3.0	3.0	100.0				
Mass	Calc. Mass	mDa	PPM	DBE	i-FIT	Norm	Conf(%)	Formula
251.1299	251.1297	0.2	0.8	10.5	603.1	n/a	n/a	C15 H15 N4

Figure S6. High resolution mass spectrum of **L-T-Pyta$_{(1,2,3)}$-Et**.

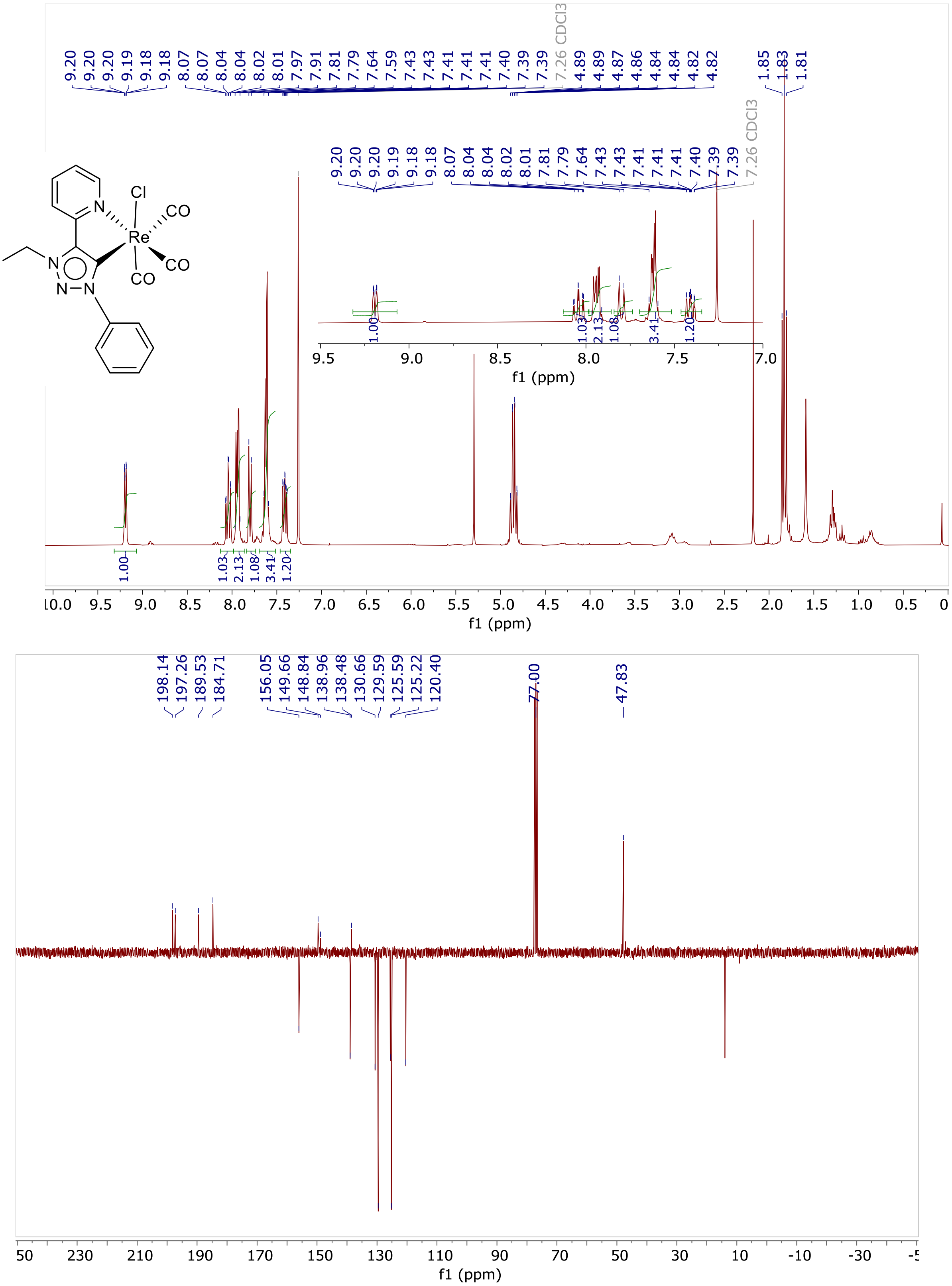

Figure S7. ^{1}H NMR (top) and ^{13}C Jmod NMR (bottom) spectra of **Re-T-Pyta$_{(1,2,3)}$-Et** in $CDCl_3$.

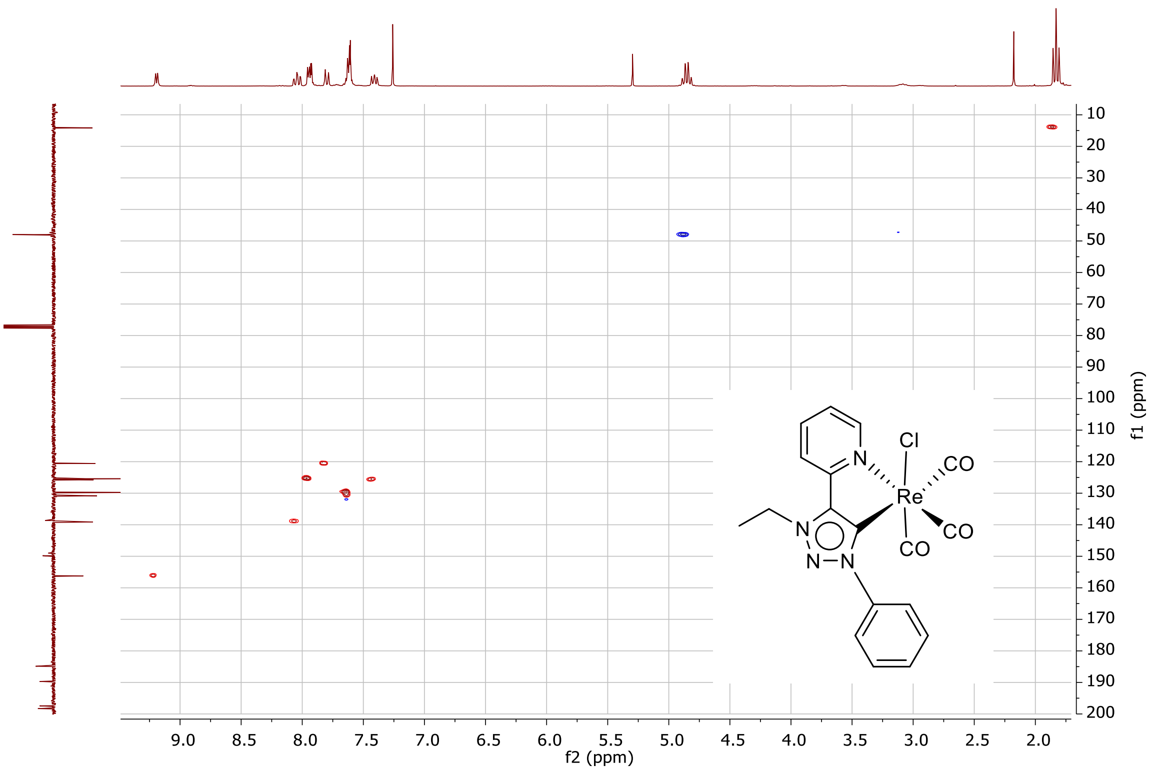

Figure S8. HSQC (bottom) spectrum of **Re-T-Pyta$_{(1,2,3)}$-Et** in $CDCl_3$.

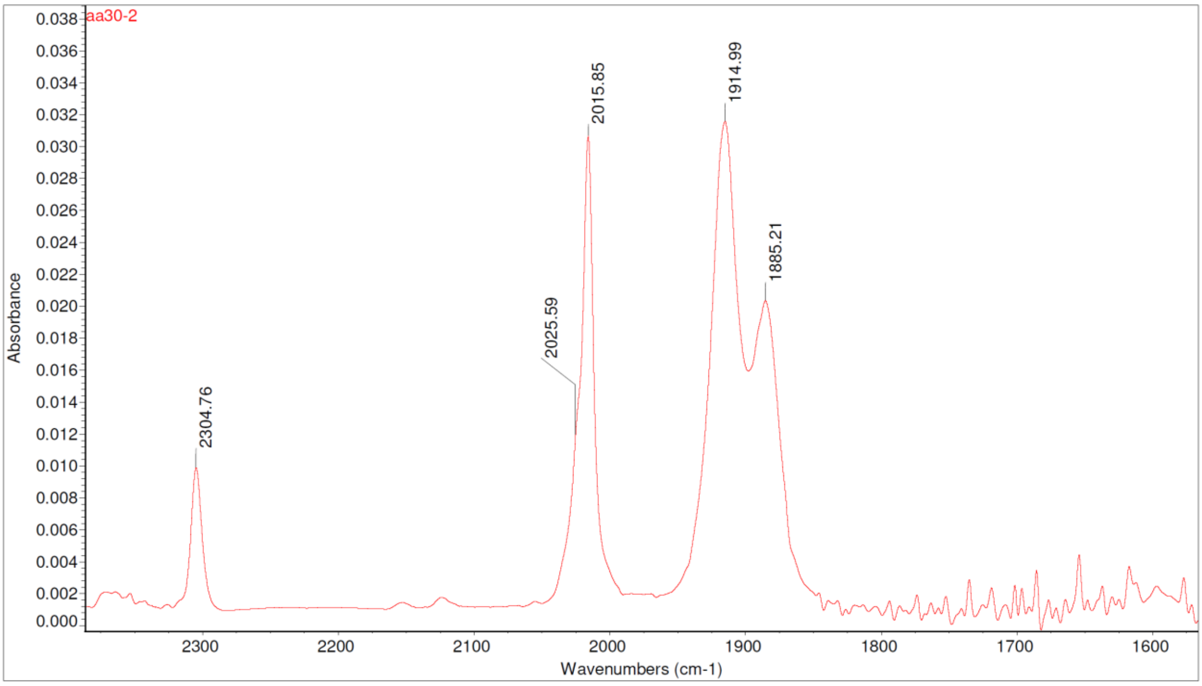

Figure S9. FT-IR spectrum of **Re-T-Pyta$_{(1,2,3)}$-Et**.

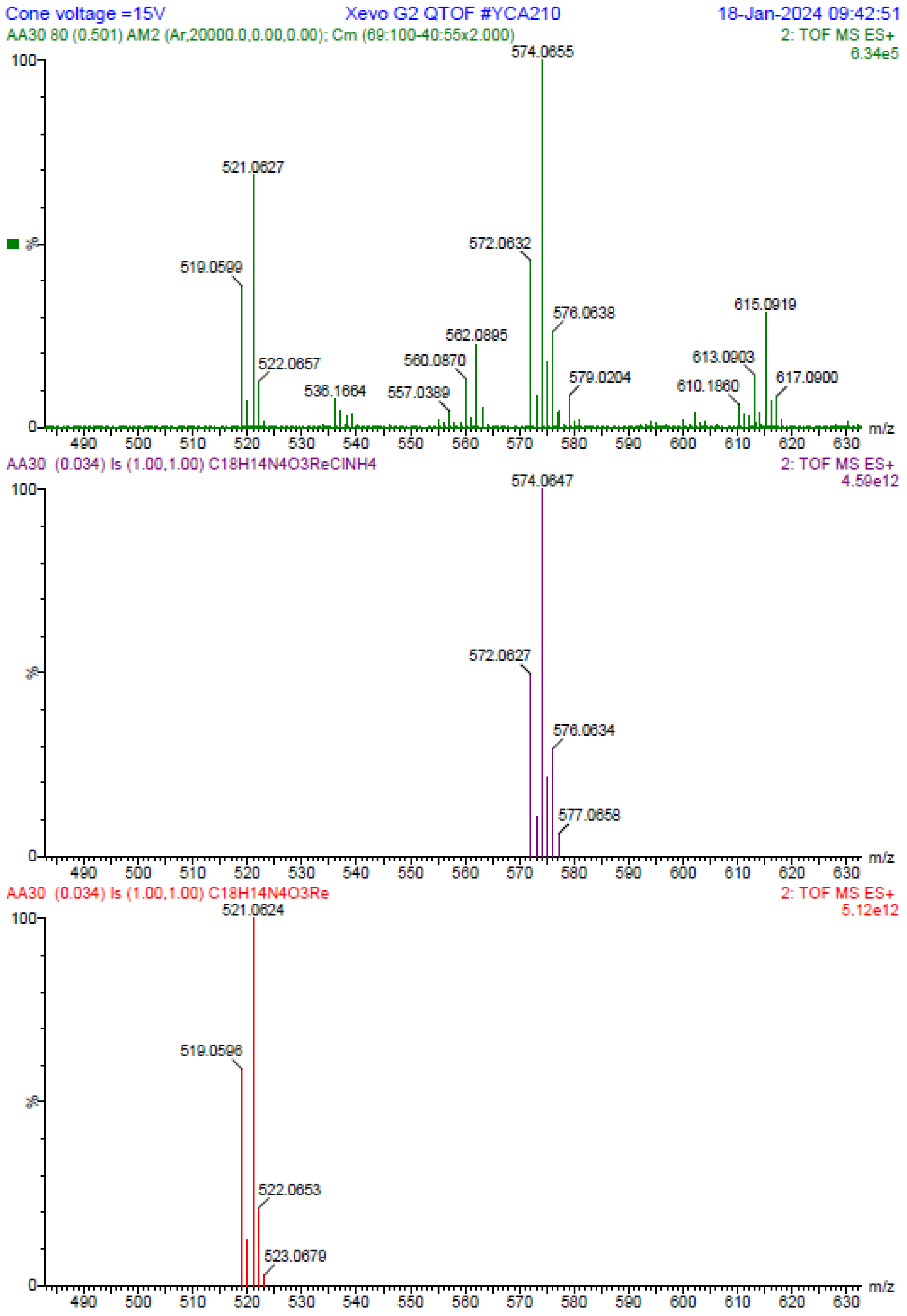

Figure S10. High resolution mass spectra of **Re-T-Pyta$_{(1,2,3)}$-Et** (data).

Elemental Composition Report

Single Mass Analysis
Tolerance = 3.0 PPM / DBE: min = -5.0, max = 100.0
Element prediction: Off
Number of isotope peaks used for i-FIT = 3

Monoisotopic Mass, Odd and Even Electron Ions
813 formula(e) evaluated with 4 results within limits (up to 50 closest results for each mass)
Elements Used:
C: 0-80 H: 0-100 N: 0-5 O: 0-5 Cl: 0-1 187Re: 0-1

Cone voltage =15V Xevo G2 QTOF #YCA210 18-Jan-2024 09:42:51
AA30 80 (0.501) AM2 (Ar,20000.0,0.00,0.00); Cm (69:100-40:55x2.000) 2: TOF MS ES+ 6.34e+005

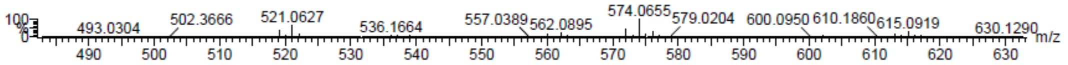

Mass	Calc. Mass	mDa	PPM	DBE	i-FIT	Norm	Conf(%)	Formula
Minimum:				-5.0				
Maximum:		3.0	3.0	100.0				
521.0627	521.0628	-0.1	-0.2	9.5	401.9	17.594	0.00	C15 H17 N5 O2 Cl 187Re
	521.0623	0.4	0.8	14.0	384.4	0.025	97.53	C18 H14 N4 O3 187Re
	521.0637	-1.0	-1.9	13.5	388.0	3.701	2.47	C20 H16 N O4 187Re
	521.0642	-1.5	-2.9	9.0	401.8	17.474	0.00	C17 H19 N2 O3 Cl 187Re

Single Mass Analysis
Tolerance = 3.0 PPM / DBE: min = -5.0, max = 100.0
Element prediction: Off
Number of isotope peaks used for i-FIT = 3

Monoisotopic Mass, Odd and Even Electron Ions
875 formula(e) evaluated with 5 results within limits (up to 50 closest results for each mass)
Elements Used:
C: 0-80 H: 0-100 N: 0-5 O: 0-5 Cl: 0-1 187Re: 0-1

Cone voltage =15V Xevo G2 QTOF #YCA210 18-Jan-2024 09:42:51
AA30 80 (0.501) AM2 (Ar,20000.0,0.00,0.00); Cm (69:100-40:55x2.000) 2: TOF MS ES+ 1.43e+005

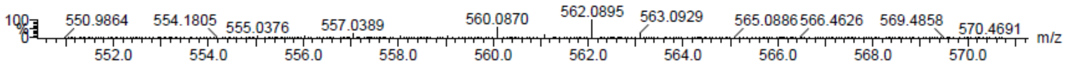

Mass	Calc. Mass	mDa	PPM	DBE	i-FIT	Norm	Conf(%)	Formula
Minimum:				-5.0				
Maximum:		3.0	3.0	100.0				
557.0389	557.0390	-0.1	-0.2	13.0	334.9	0.511	59.96	C18 H15 N4 O3 Cl 187Re
	557.0391	-0.2	-0.4	44.5	343.4	9.059	0.01	C46 H5
	557.0385	0.4	0.7	17.5	342.4	8.041	0.03	C21 H12 N3 O4 187Re
	557.0399	-1.0	-1.8	17.0	343.0	8.605	0.02	C23 H14 O5 187Re
	557.0404	-1.5	-2.7	12.5	335.3	0.917	39.98	C20 H17 N O4 Cl 187Re

Single Mass Analysis
Tolerance = 3.0 PPM / DBE: min = -5.0, max = 100.0
Element prediction: Off
Number of isotope peaks used for i-FIT = 3

Monoisotopic Mass, Odd and Even Electron Ions
905 formula(e) evaluated with 5 results within limits (up to 50 closest results for each mass)
Elements Used:
C: 0-80 H: 0-100 N: 0-5 O: 0-5 Cl: 0-1 187Re: 0-1

Cone voltage =15V Xevo G2 QTOF #YCA210 18-Jan-2024 09:42:51
AA30 80 (0.501) AM2 (Ar,20000.0,0.00,0.00); Cm (69:100-40:55x2.000) 2: TOF MS ES+ 6.34e+005

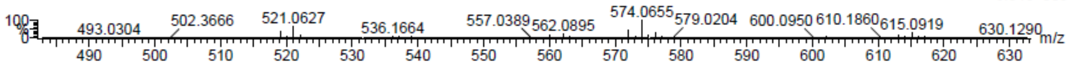

Mass	Calc. Mass	mDa	PPM	DBE	i-FIT	Norm	Conf(%)	Formula
Minimum:				-5.0				
Maximum:		3.0	3.0	100.0				
574.0655	574.0656	-0.1	-0.2	12.0	327.3	0.075	92.74	C18 H18 N5 O3 Cl 187Re
	574.0657	-0.2	-0.3	43.5	342.1	14.826	0.00	C46 H8 N
	574.0651	0.4	0.7	16.5	340.3	13.072	0.00	C21 H15 N4 O4 187Re
	574.0664	-0.9	-1.6	16.0	340.7	13.475	0.00	C23 H17 N O5 187Re
	574.0669	-1.4	-2.4	11.5	329.8	2.623	7.26	C20 H20 N2 O4 Cl 187Re

Figure S11. High resolution mass spectra of **Re-T-Pyta$_{(1,2,3)}$-Et** (calculations).

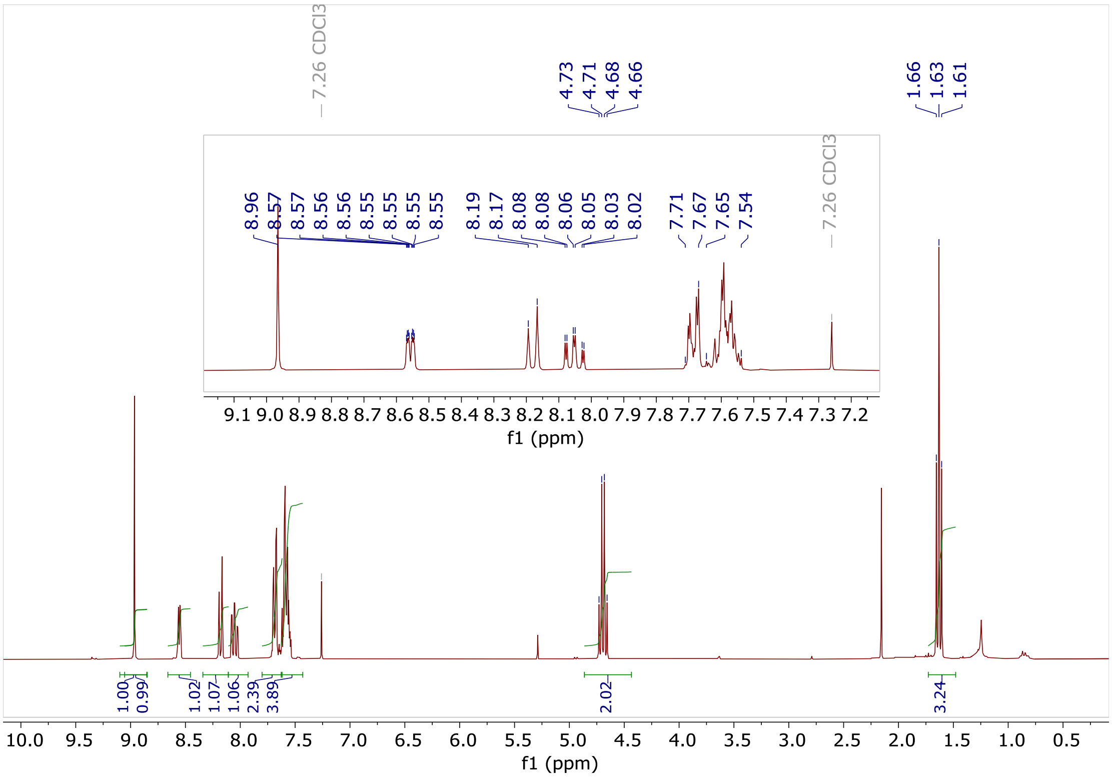

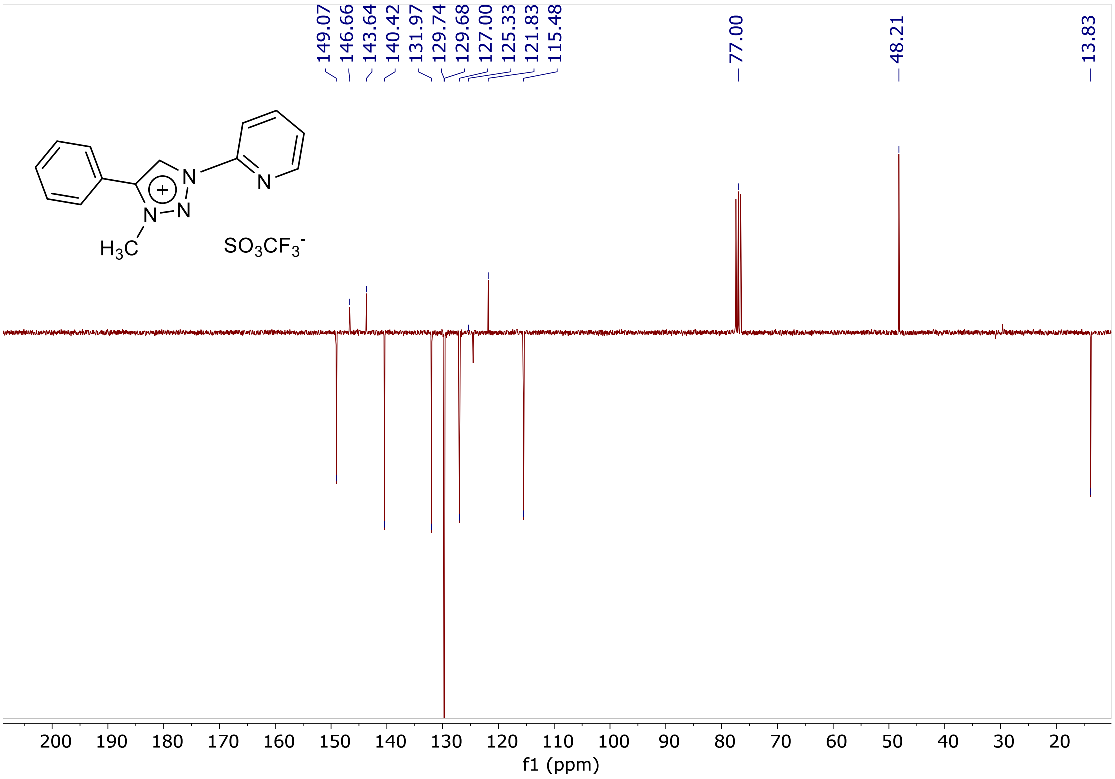

Figure S12. ^{1}H NMR (top) and ^{13}C Jmod NMR (bottom) spectra of **L-T-Tapy-Me** in $CDCl_3$.

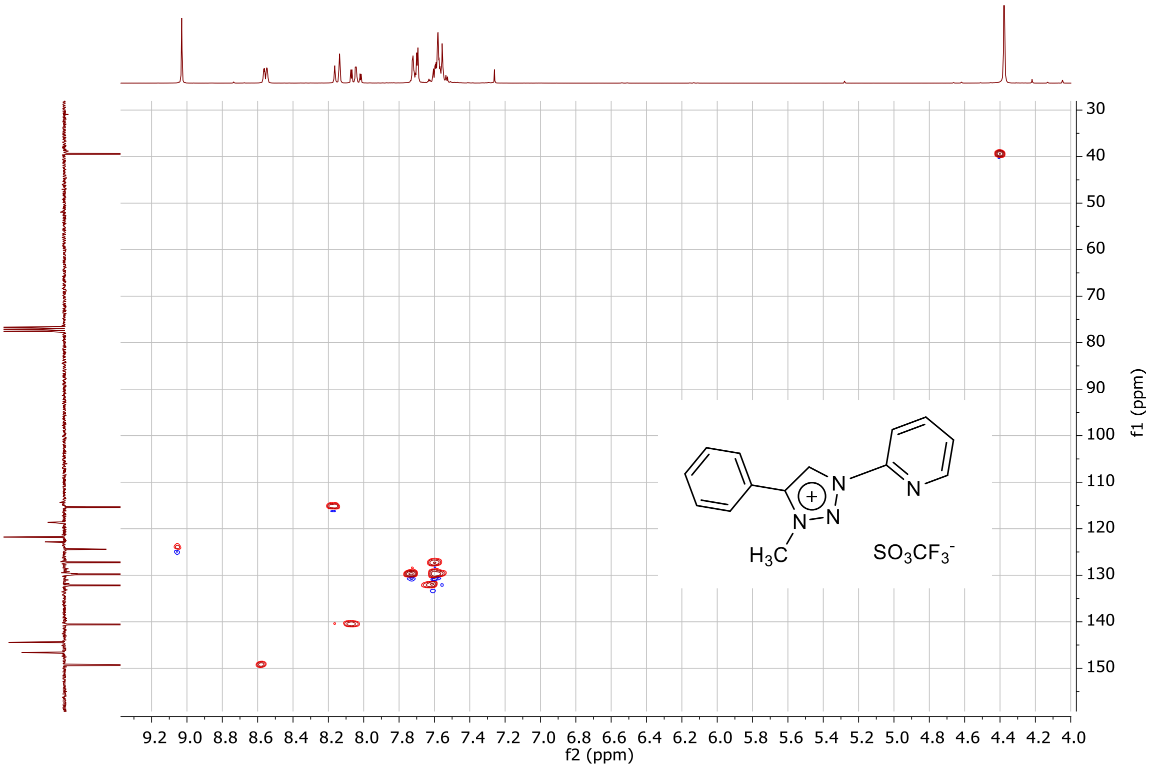

Figure S13. HSQC spectrum of **L-T-Tapy-Me** in $CDCl_3$.

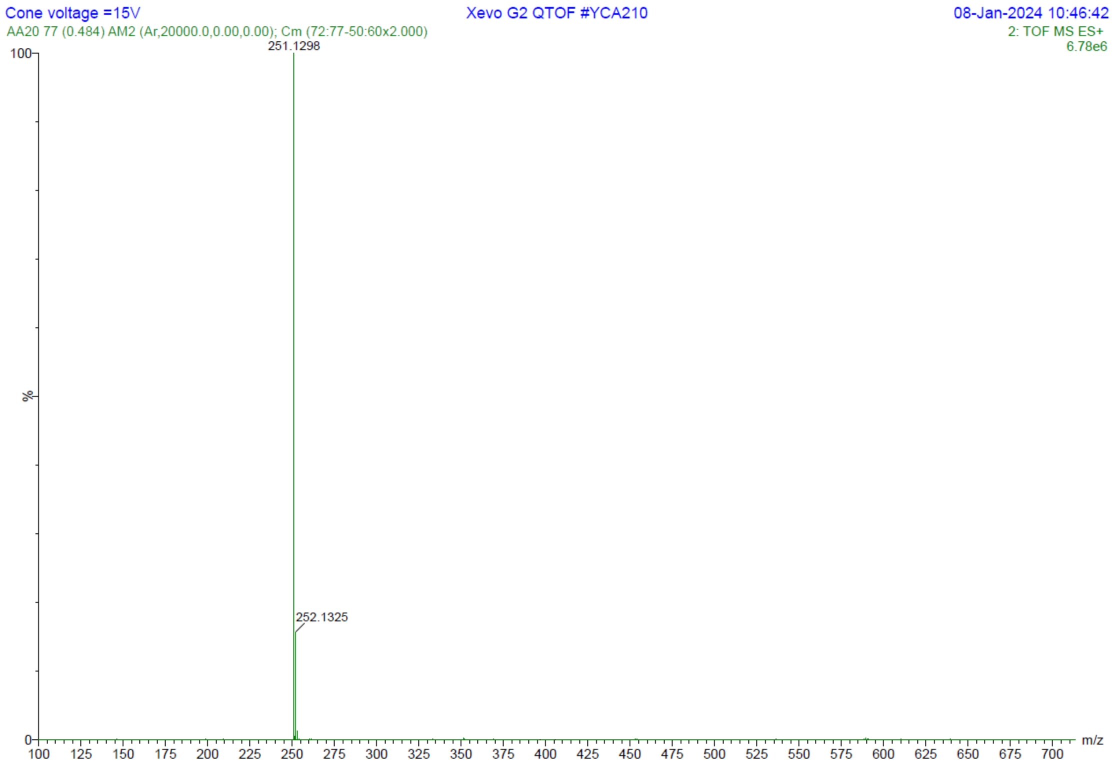

Elemental Composition Report

Page 1

Single Mass Analysis

Tolerance = 3.0 PPM / DBE: min = -5.0, max = 100.0
Element prediction: Off
Number of isotope peaks used for i-FIT = 3

Monoisotopic Mass, Even Electron Ions
352 formula(e) evaluated with 1 results within limits (up to 50 closest results for each mass)
Elements Used:
C: 0-80 H: 0-100 N: 0-10 O: 0-10

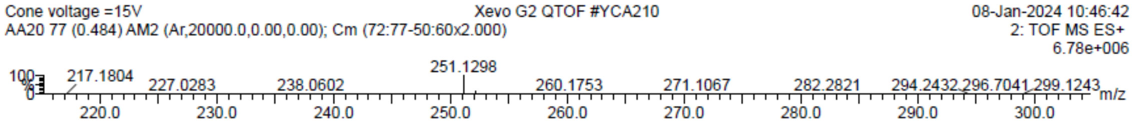

Minimum:				-5.0
Maximum:		3.0	3.0	100.0

Mass	Calc. Mass	mDa	PPM	DBE	i-FIT	Norm	Conf(%)	Formula
251.1298	251.1297	0.1	0.4	10.5	660.0	n/a	n/a	C15 H15 N4

Figure S14. High resolution mass spectrum of **L-T-Tapy-Me**.

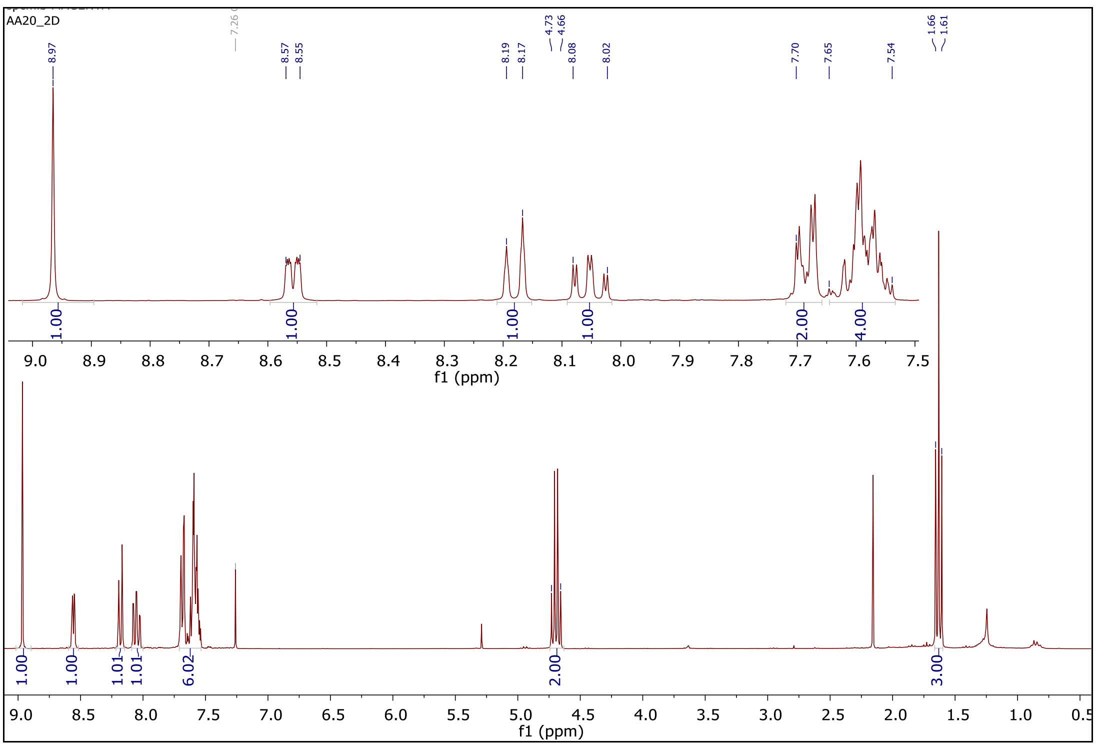

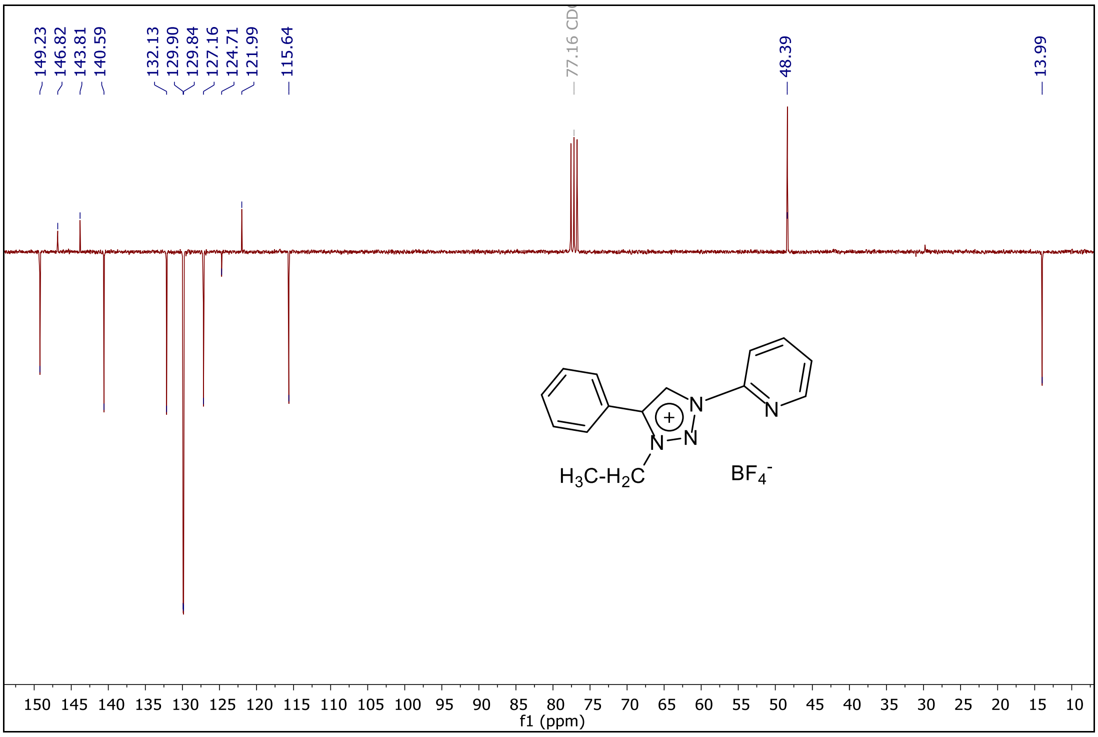

Figure S15. ^{1}H NMR (top) and ^{13}C Jmod NMR (bottom) spectra of **L-T-Tapy-Et** in $CDCl_3$.

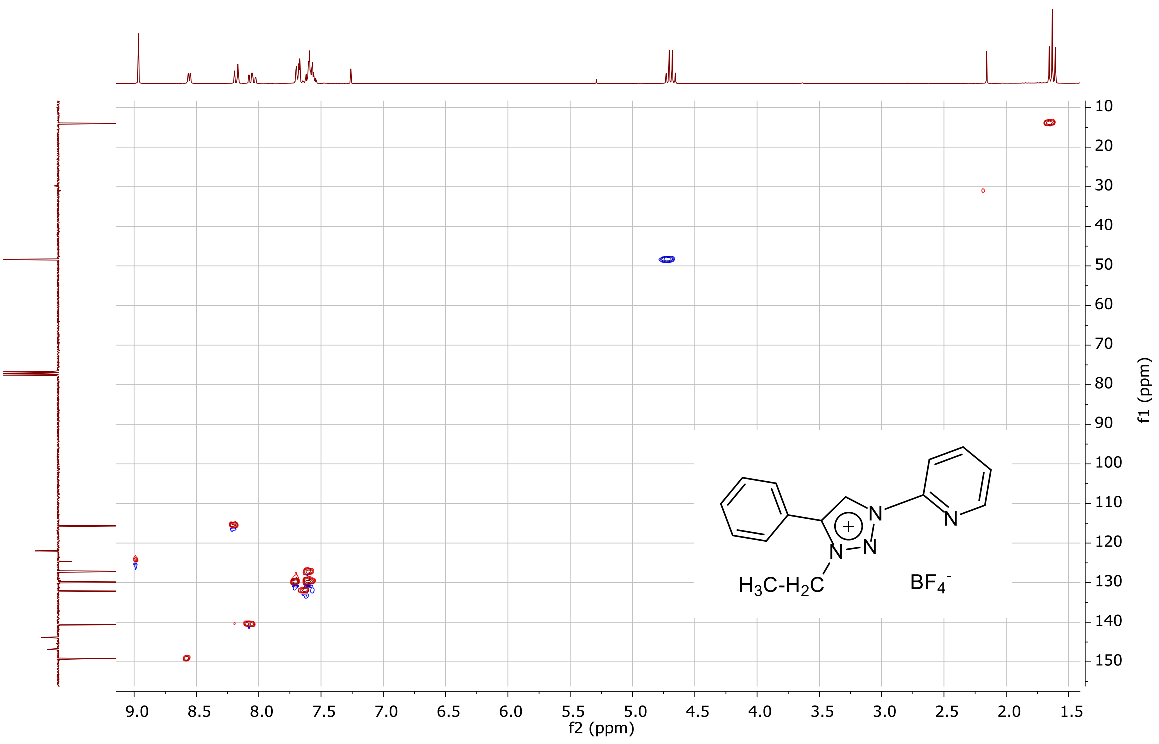

Figure S16. HSQC spectra of **L-T-Tapy-Et** in $CDCl_3$.

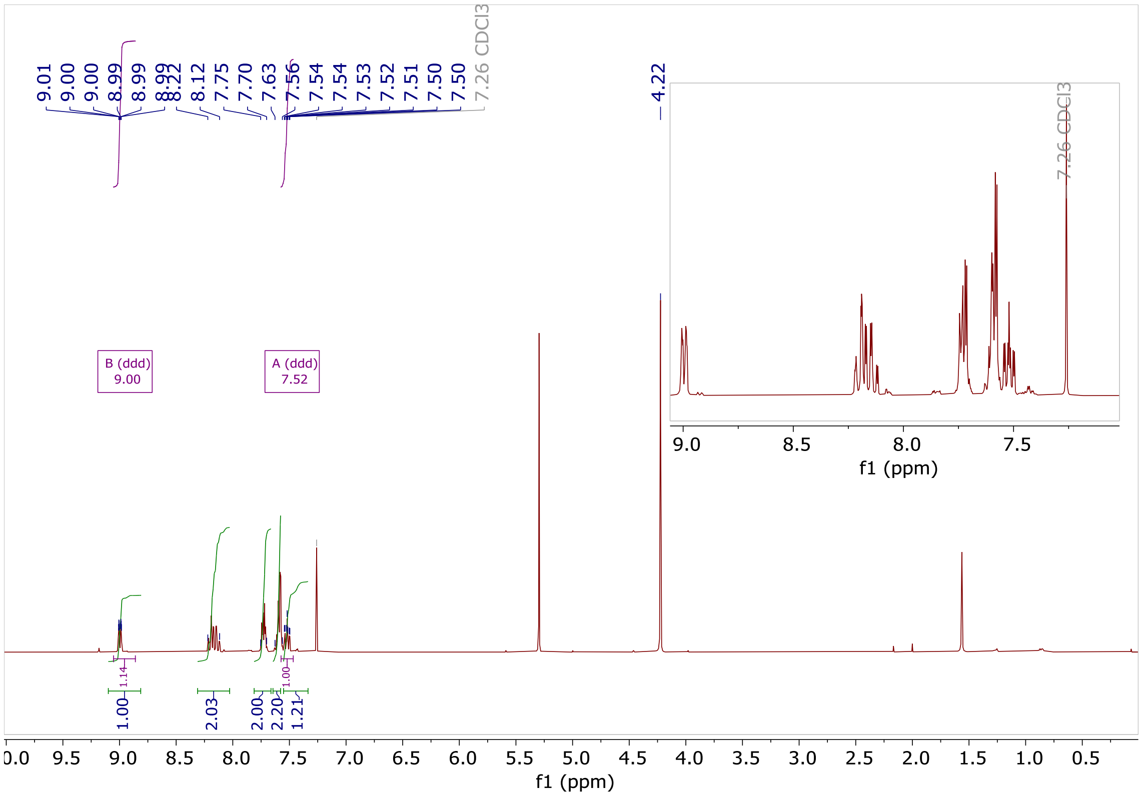

Figure S17. ^{1}H NMR spectrum of **Re-T-Tapy-Me** in $CDCl_3$.

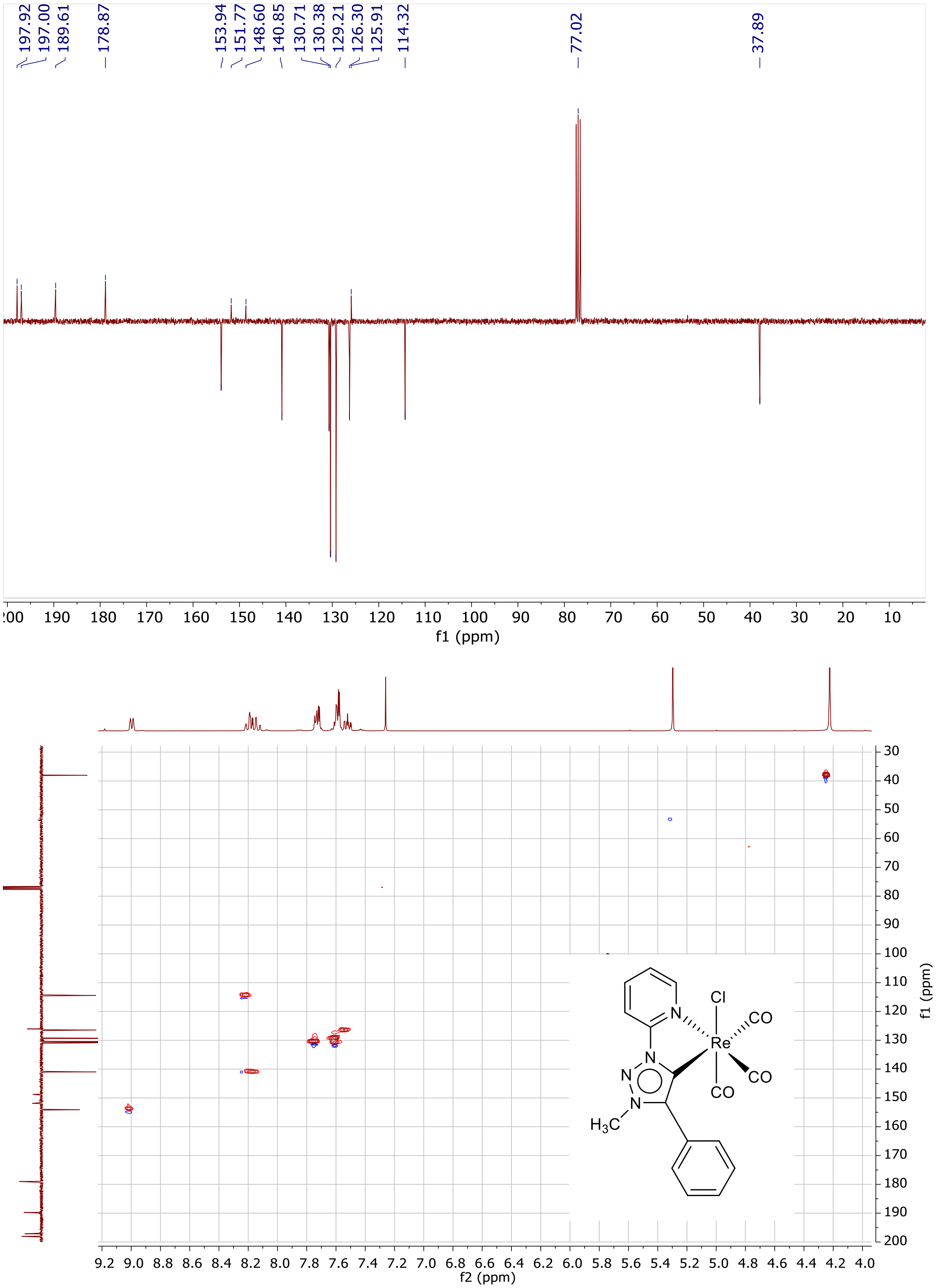

Figure S18. ^{13}C Jmod NMR (top) and HSQC (bottom) spectra of **Re-T-Tapy-Me** in $CDCl_3$.

Elemental Composition Report

Single Mass Analysis

Tolerance = 3.0 PPM / DBE: min = -5.0, max = 100.0
Element prediction: Off
Number of isotope peaks used for i-FIT = 3

Monoisotopic Mass, Odd and Even Electron Ions
789 formula(e) evaluated with 3 results within limits (up to 50 closest results for each mass)
Elements Used:
C: 0-80 H: 0-100 N: 0-5 O: 0-5 Cl: 0-1 187Re: 0-1

Cone voltage =15V Xevo G2 QTOF #YCA210 18-Jan-2024 09:38:49
AA31 83 (0.528) AM2 (Ar,20000.0,0.00,0.00); Cm (71:97-45:59x2.000) 2: TOF MS ES+ 8.23e+005

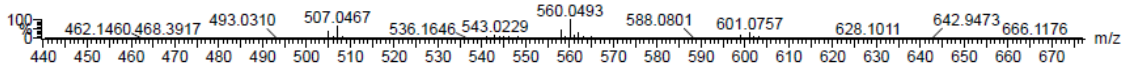

Mass	Calc. Mass	mDa	PPM	DBE	i-FIT	Norm	Conf (%)	Formula
Minimum:				-5.0				
Maximum:		3.0	3.0	100.0				
507.0467	507.0467	0.0	0.0	14.0	407.7	0.006	99.44	C17 H12 N4 O3 187Re
	507.0472	-0.5	-1.0	9.5	427.6	19.940	0.00	C14 H15 N5 O2 Cl 187Re
	507.0480	-1.3	-2.6	13.5	412.9	5.183	0.56	C19 H14 N O4 187Re

Elemental Composition Report

Single Mass Analysis

Tolerance = 3.0 PPM / DBE: min = -5.0, max = 100.0
Element prediction: Off
Number of isotope peaks used for i-FIT = 3

Monoisotopic Mass, Odd and Even Electron Ions
851 formula(e) evaluated with 5 results within limits (up to 50 closest results for each mass)
Elements Used:
C: 0-80 H: 0-100 N: 0-5 O: 0-5 Cl: 0-1 187Re: 0-1

Cone voltage =15V Xevo G2 QTOF #YCA210 18-Jan-2024 09:38:49
AA31 83 (0.528) AM2 (Ar,20000.0,0.00,0.00); Cm (71:97-45:59x2.000) 2: TOF MS ES+ 1.11e+005

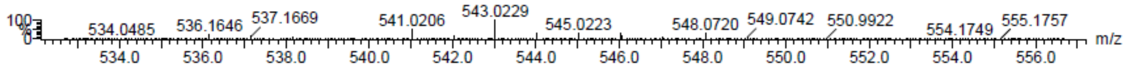

Mass	Calc. Mass	mDa	PPM	DBE	i-FIT	Norm	Conf (%)	Formula
Minimum:				-5.0				
Maximum:		3.0	3.0	100.0				
543.0229	543.0229	0.0	0.0	17.5	341.3	10.011	0.00	C20 H10 N3 O4 187Re
	543.0234	-0.5	-0.9	13.0	331.4	0.188	82.86	C17 H13 N4 O3 Cl 187Re
	543.0235	-0.6	-1.1	44.5	341.7	10.423	0.00	C45 H3
	543.0242	-1.3	-2.4	17.0	341.6	10.325	0.00	C22 H12 O5 187Re
	543.0213	1.6	2.9	35.5	333.0	1.764	17.13	C39 H8 O2 Cl

Figure S19. High resolution mass spectrum of **Re-T-Tapy-Me**.

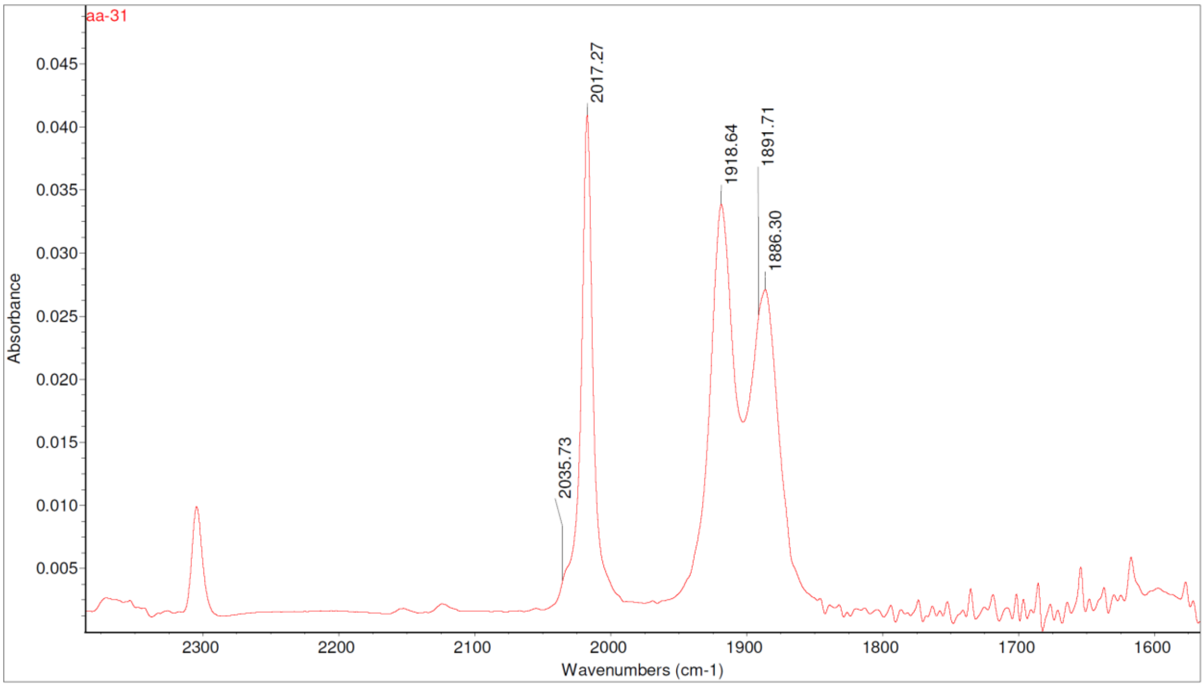

Figure S20. FT-IR spectrum of **Re-T-Tapy-Me**.

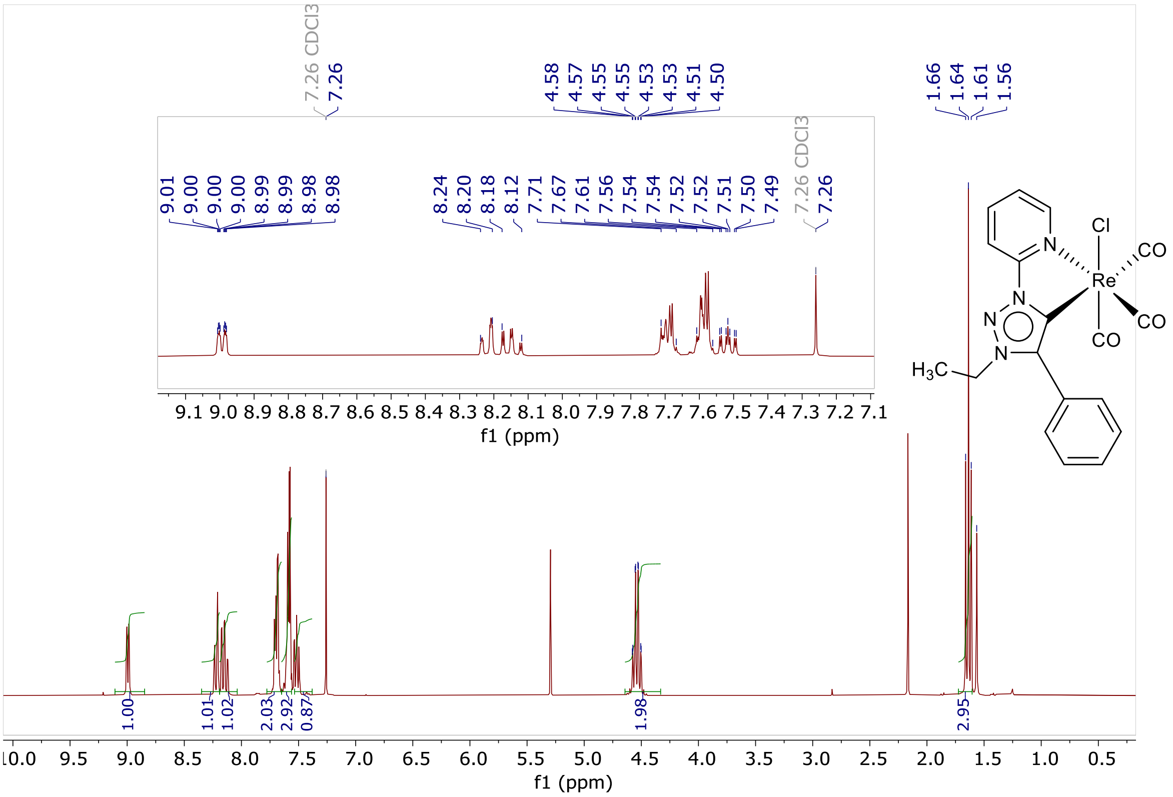

Figure S21. ^{1}H NMR spectrum of **Re-T-Tapy-Et** in $CDCl_3$.

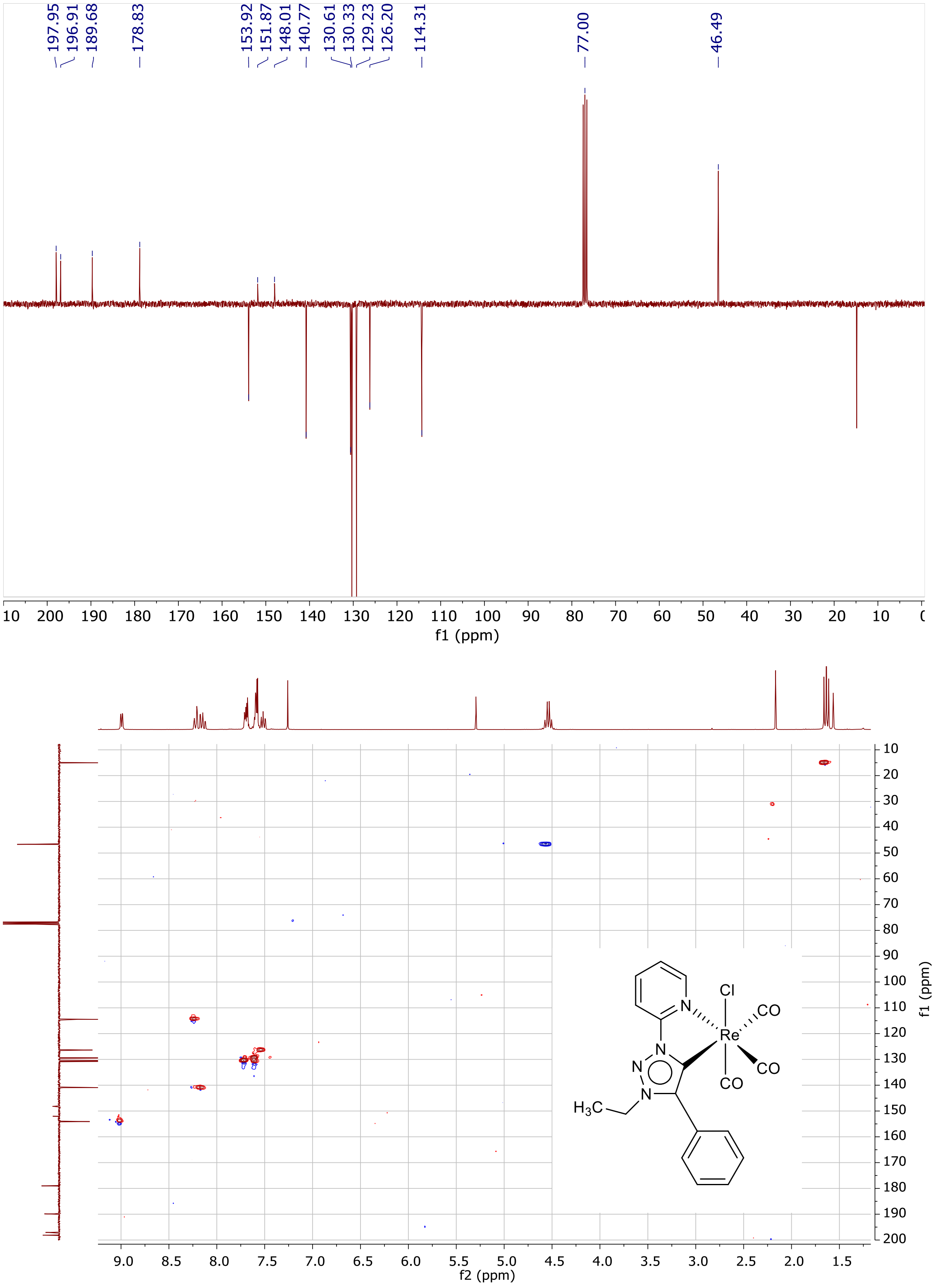

Figure S22. ^{13}C Jmod NMR (top) and HSQC (bottom) spectrum of **Re-T-Tapy-Et** in $CDCl_3$.

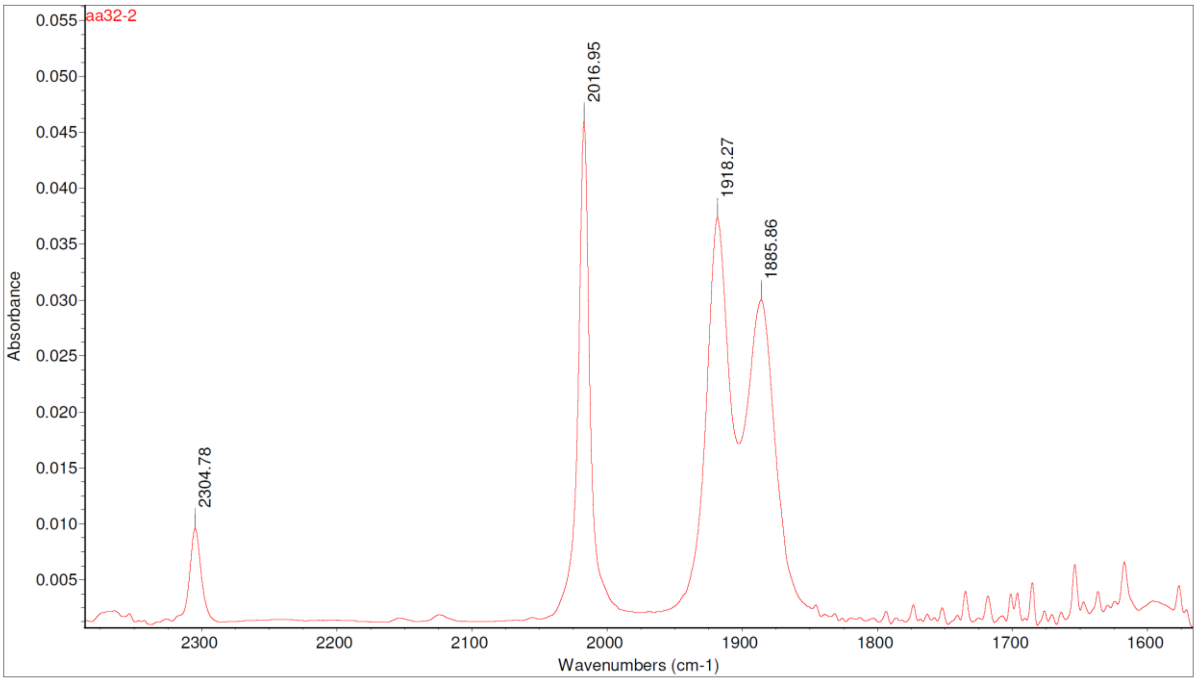

Figure S23. FT-IR spectrum of **Re-T-Tapy-Et**

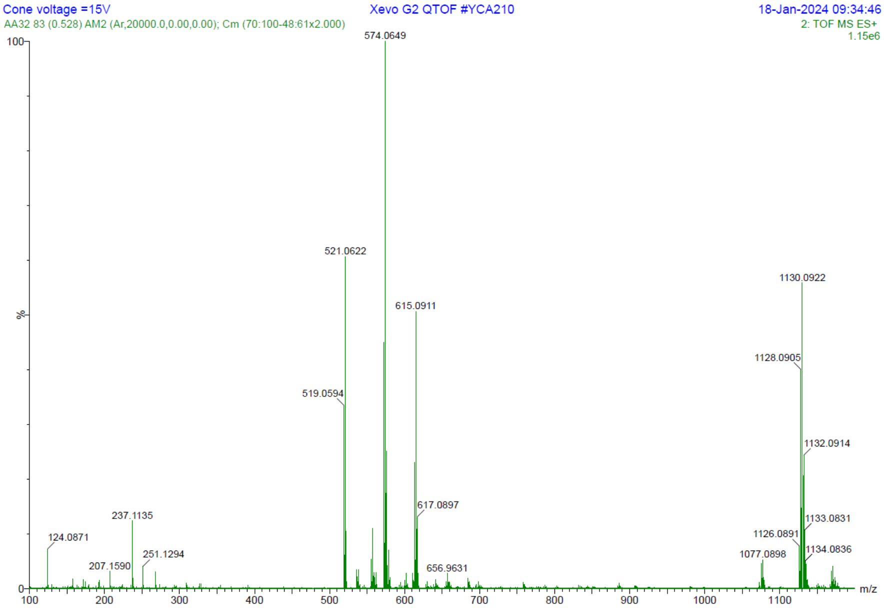

Figure S24. High resolution mass spectrum of **Re-T-Tapy-Et** (data).

Elemental Composition Report

Single Mass Analysis

Tolerance = 3.0 PPM / DBE: min = -5.0, max = 100.0
Element prediction: Off
Number of isotope peaks used for i-FIT = 3

Monoisotopic Mass, Odd and Even Electron Ions
1427 formula(e) evaluated with 9 results within limits (up to 50 closest results for each mass)
Elements Used:
C: 0-80 H: 0-100 N: 0-10 O: 0-5 Cl: 0-1 187Re: 0-1

Cone voltage =15V Xevo G2 QTOF #YCA210 18-Jan-2024 09:34:46
AA32 83 (0.528) AM2 (Ar,20000.0,0.00,0.00); Cm (70:100-48:61x2.000) 2: TOF MS ES+ 1.15e+006

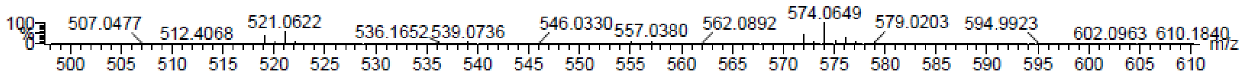

Minimum:				-5.0				
Maximum:		3.0	3.0	100.0				
Mass	Calc. Mass	mDa	PPM	DBE	i-FIT	Norm	Conf(%)	Formula
521.0622	521.0623	-0.1	-0.2	14.0	394.5	0.141	86.82	C18 H14 N4 O3 187Re
	521.0621	0.1	0.2	29.0	400.6	6.313	0.18	C27 H7 N9 O4
	521.0626	-0.4	-0.8	24.5	413.7	19.358	0.00	C24 H10 N10 O3 Cl
	521.0628	-0.6	-1.2	9.5	414.0	19.673	0.00	C15 H17 N5 O2 Cl 187Re
	521.0615	0.7	1.3	10.0	414.2	19.859	0.00	C13 H15 N8 O Cl 187Re
	521.0634	-1.2	-2.3	28.5	401.7	7.337	0.07	C29 H9 N6 O5
	521.0610	1.2	2.3	14.5	396.5	2.212	10.95	C16 H12 N7 O2 187Re
	521.0637	-1.5	-2.9	13.5	398.2	3.922	1.98	C20 H16 N O4 187Re
	521.0607	1.5	2.9	32.0	413.7	19.380	0.00	C37 H12 N O Cl

Elemental Composition Report

Single Mass Analysis

Tolerance = 3.0 PPM / DBE: min = -5.0, max = 100.0
Element prediction: Off
Number of isotope peaks used for i-FIT = 3

Monoisotopic Mass, Odd and Even Electron Ions
1596 formula(e) evaluated with 10 results within limits (up to 50 closest results for each mass)
Elements Used:
C: 0-80 H: 0-100 N: 0-10 O: 0-5 Cl: 0-1 187Re: 0-1

Cone voltage =15V Xevo G2 QTOF #YCA210 18-Jan-2024 09:34:46
AA32 83 (0.528) AM2 (Ar,20000.0,0.00,0.00); Cm (70:100-48:61x2.000) 2: TOF MS ES+ 1.15e+006

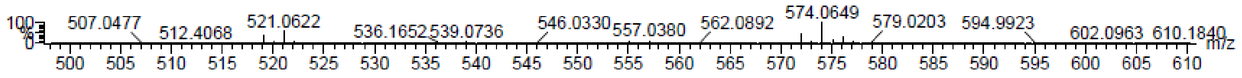

Minimum:				-5.0				
Maximum:		3.0	3.0	100.0				
Mass	Calc. Mass	mDa	PPM	DBE	i-FIT	Norm	Conf(%)	Formula
574.0649	574.0648	0.1	0.2	31.5	370.1	12.573	0.00	C30 H8 N9 O5
	574.0651	-0.2	-0.3	16.5	369.7	12.233	0.00	C21 H15 N4 O4 187Re
	574.0653	-0.4	-0.7	27.0	359.5	1.972	13.92	C27 H11 N10 O4 Cl
	574.0642	0.7	1.2	12.5	358.5	1.041	35.32	C16 H16 N8 O2 Cl 187Re
	574.0656	-0.7	-1.2	12.0	358.2	0.694	49.98	C18 H18 N5 O3 Cl 187Re
	574.0657	-0.8	-1.4	43.5	371.6	14.085	0.00	C46 H8 N
	574.0637	1.2	2.1	17.0	369.3	11.834	0.00	C19 H13 N7 O3 187Re
	574.0635	1.4	2.4	34.5	362.3	4.855	0.78	C40 H13 N O2 Cl
	574.0664	-1.5	-2.6	21.5	370.1	12.586	0.00	C22 H11 N8 187Re
	574.0664	-1.5	-2.6	16.0	370.2	12.693	0.00	C23 H17 N O5 187Re

Figure S25. High resolution mass spectrum of **Re-T-Tapy-Et** (calculations).

Crystallography

1. Octahedral distortion parameters of complexes Re-Tapy, Re-T-Tapy-Me and Re-T-Tapy-Et.
Octahedral distortion parameters are composed of three parameters: one bond-length distortion parameter ζ and two bond-angle distortion parameters Σ and Θ. ζ is the average of the sum of the deviation of 6 unique metal–ligand bond lengths around the central metal atom (d_i) from the average value (d_{mean}). *Σ* can be defined as the sum of the deviation of the 12 *cis* L–Re–L angles ϕ_i from 90°. *Σ* is a general measure of the deviation of a metal ion from an ideal octahedral geometry. *Θ* can be defined as the sum of the deviation of the 24 torsional angles between the ligand atoms on opposite triangular faces of the octahedron viewed along the pseudo-threefold axis (θ_i) from 60°. *Θ* represents the distortion of the MX_6 geometry from perfectly octahedral (O_h) to trigonal prismatic (D_{3h}).
Distortion parameters ζ, Σ and Θ, as well as the volume of the octahedron, were calculated using the OctaDist 3.1.0 software [1]. Distortion parameters and the volume of the octahedron calculated for **Re-Tapy**, **Re-T-Tapy-Me** and **Re-T-Tapy-Et** are listed in Table S1. All values lie in the expected range observed for distorted quasi-octahedral Re(I) complexes. In fact, a perfectly octahedral complex would give ζ = Σ = Θ = 0.

Table S1. Experimental and DFT-optimized octahedral distortion parameters in the singlet (S_0, ground; S_1, excited) and triplet (T_1, excited) states of complexes **Re-Tapy**, **Re-T-Tapy-Me** and **Re-T-Tapy-Et**.

**Complex**	**Re-T-Tapy-Et**	**Re-Tapy**				**Re-T-Tapy-Me**			
**Parameter**	**Exp.**	**Exp.**	**Calc.**			**Exp.**	**Calc.**		
			**S_0**	**S_1**	**T_1**		**S_0**	**S_1**	**T_1**
ζ, Å	1.01	1.04	1.13	0.80	0.92	1.09	1.15	0.83	0.87
Σ, °	57.6	64.5	61.0	54.0	50.9	62.1	55.5	53.3	54.0
Θ, °	157	167	170	189	169	156	153	167	173
V, Å^3	12.30	11.98	12.01	11.85	11.87	12.18	12.22	12.07	12.09
μ, D	–	–	6.35	2.93	4.08	–	5.98	2.39	2.86

2- Intermolecular interactions in crystal structures of complexes Re-Tapy, Re-T-Tapy-Me and Re-T-Tapy-Et and **Re-T-Pyta$_{(1,2,3)}$-Et**.
Molecular graphics were created by Mercury software [2].

Table S2. Short contacts detected in structures of **Re-Tapy**, **Re-T-Tapy-Me**, **Re-T-Tapy-Et** and **Re-T-Pyta$_{(1,2,3)}$-Et**.

D—H•••A	D—H [Å]	H•••A [Å]	D•••A [Å]	D—H•••A [°]	Symmetry codes
**Re-Tapy**					
C7—H7•••O4	0.95	2.52	3.450(11)	166	*x*, *y*, 1+*z*
C9—H9•••O4	0.95	2.12	3.056(11)	170	*x*, *y*, 1+*z*
C9—H9•••O4‘	0.95	2.05	2.97(6)	164' 17' 351	*x*, *y*, 1+*z*
C16—H16•••N4	0.95	2.56	2.889(10)	100	–
C18—H18A•••Cl1	0.98	2.75	3.606(15)	146	1+*x*, *y*, *z*
**Re-T-Tapy-Me**					
C5—H5•••O3	0.95	2.54	3.214(3)	128	1–*x*, 1–*y*, –*z*
C18—H18A•••Cl1	0.99	2.61	3.534(3)	156	1–*x*, –*y*, 1–*z*
C18—H18B•••Cl1	0.99	2.75	3.610(3)	146	*x*, *y*, 1+*z*
**Re-T-Tapy-Et**					
C4—H4•••O3	0.95	2.39	3.241(5)	150	–*x*, –*y*, 1–*z*
C14—H14•••O2	0.95	2.43	3.234(7)	142	–*x*, 1/2+*y*, 3/2–*z*

[1] R. Ketkaew, Y. Tantirungrotechai, P. Harding, G. Chastanet, P. Guionneau, M. Marchivie, D. J. Harding, *Dalton Trans.*, **2021**, *50*, 1086–1096.
[2] C.F. Macrae, I. Sovago, S.J. Cottrell, P.T.A. Galek, P. McCabe, E. Pidcock, M. Platings, G. P. Shields, J. S. Stevens, M. Towler, P. A. Wood, *J. Appl. Crystallogr.*, **2020**, *53*, 226–235.

C15—H15•••Cl1	0.95	2.64	3.595(4)	179	1–*x*, 1/2+*y*, 3/2–*z*
		**Re-T-Pyta$_{(1,2,3)}$-Et**			
C4—H4•••Cl2	0.95	2.77	3.466(5)	130	*x*, –1+*y*, *z*
C7—H7•••Cl1	0.95	2.66	3.398(5)	135	1–*x*, –*y*, 1–*z*
C13—H13•••O1	0.95	2.49	3.243(7)	136	1+*x*, *y*, *z*
C25—H25•••Cl2	0.95	2.78	3.525(5)	136	–*x*, 2–*y*, –*z*
C35—H35A•••Cl1	0.99	2.79	3.751(5)	164	1–*x*, 1–*y*, –*z*

Table S3. Geometrical parameters (Å, °) for C–H···π interactions detected in structures of complexes **Re-Tapy**, **Re-T-Tapy-Me** and **Re-T-Tapy-Et**.

X—H(i)•••Cg(i)	H···Cg [Å]	X···Cg [Å]	X–H···Cg [°]	H-Perp	Gamma
		**Re-Tapy**			
C17—H17A•••Cg(4)$^{\#1}$	2.97	3.411(11)	108	–2.95	7.43
C17—H17E•••Cg(4)$^{\#1}$	2.50	3.411(11)	154	–2.50	2.86
		**Re-T-Tapy-Me**			
C16—H16•••Cg(2)$^{\#2}$	2.86	3.536(2)	129	–2.86	2.08
		**Re-T-Tapy-Et**			
C16—H16•••Cg(4)$^{\#3}$	2.90	3.488(5)	122	2.71	20.39

For complex **Re-Tapy**, Cg(4) is the centroid of the ring (C11–C16).
For complex **Re-T-Tapy-Me**, Cg(2) is the centroid of the ring (N2/N3/N4/C10/C9).
For complex **Re-T-Tapy-Et**, Cg(4) is the centroid of the ring (C11–C16).
Symmetry codes: #1: *1–x, 1/2+y, 1–z*; #2: *1–x, –y,1–z*; #3: 1–*x*, –1/2+*y*, 3/2–*z*.

Table S4. Geometrical parameters (Å, °) for *π*···*π* interactions detected in structure of complexes **Re-Tapy**, **Re-T-Tapy-Et** and **Re-T-Pyta$_{(1,2,3)}$-Et**.

Cg(i)•••Cg(j)	Cg···Cg [Å]	*α* [°]	*β* [°]	*γ* [°]	Cg(i)_Perp	Cg(j)_Perp	Slippage
			**Re-Tapy**				
Cg(3)•••Cg(4)$^{\#1}$	3.955(5)	3.8(4)	29.7	28.6	3.473(3)	3.436(4)	1.958
Cg(4)•••Cg(3)$^{\#2}$	3.955(5)	3.8(4)	28.6	29.7	3.436(4)	3.473(3)	1.893
			**Re-T-Tapy-Et**				
Cg(3)•••Cg(3)$^{\#3}$	3.489(2)	0.00(18)	19.3	19.3	3.2928(15)	3.2926(15)	1.155
			**Re-T-Pyta$_{(1,2,3)}$-Et**				
Cg(6)•••Cg(7)$^{\#4}$	3.962(3)	9.2(2)	27.6	30.2	3.4263(18)	3.5120(19)	1.835
Cg(7)•••Cg(6)$^{\#4}$	3.962(3)	9.2(2)	30.2	27.6	3.5121(19)	3.4263(18)	1.990

Symmetry codes: #1: –1+*x*, *y*, *z*; #2: 1+*x*, *y*, *z*; #3: 1–*x*, 1–*y*, 1–*z*.
Cg(3) and Cg(4) of **Re-Tapy** are the centroids of the rings (N1/C4–C8) and (C11–C16), respectively.
Cg(3) of **Re-T-Tapy-Et** is the centroid of the ring (N1/C4–C8).
Cg(6) and Cg(7) of **Re-T-Pyta$_{(1,2,3)}$-Et** are the centroids of the rings (N6–N8/C27/C28) and (N5/C22–C25), respectively.

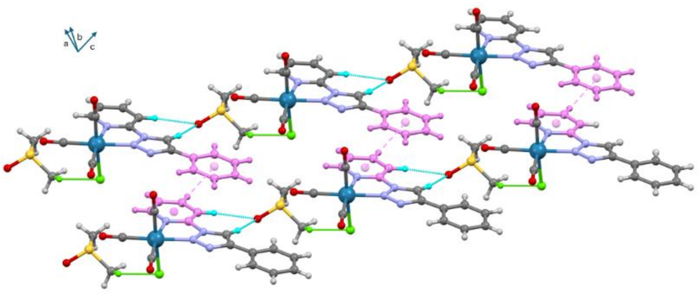

Figure S26. (a) Molecules of **Re-Tapy** linked by DMSO *via* intermolecular C7–H7$_{(py)}$···O4$_{(DMSO)}$, C9–H9$_{(trz)}$···O4$_{(DMSO)}$ (cyan color) and C18–H18A$_{(DMSO)}$···Cl1 (neon green color) interactions into a stair-

type 1D chain viewed propagating in the *ac* direction. (b) $\pi_{(Ph)}\cdots\pi_{(py)}$ (violet color) stacking interactions stabilizing the structure of **Re-Tapy**.

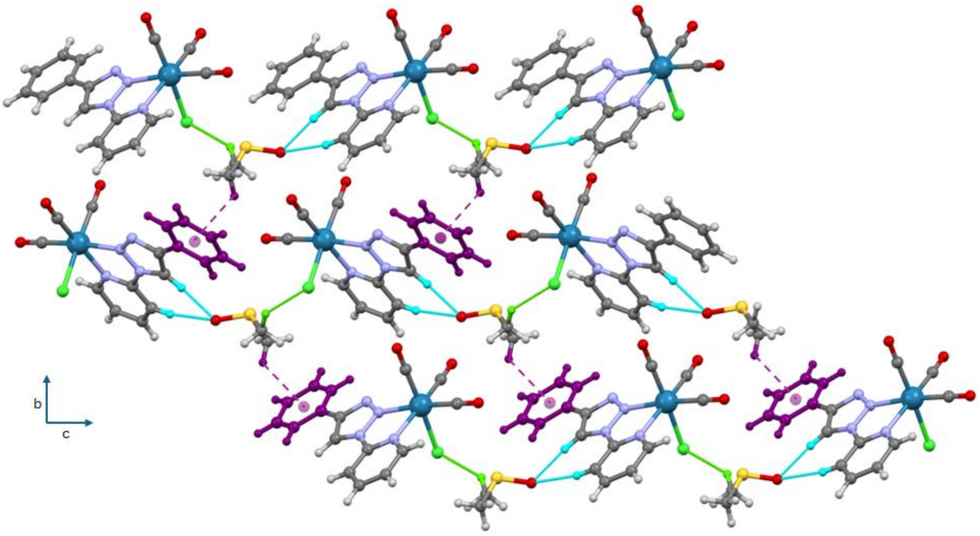

Figure S27. Weak intramolecular C17–H17$_{(DMSO)}\cdots\pi_{(Ph)}$ (tyrian purple color) interactions stabilizing the structure of **Re-Tapy**.

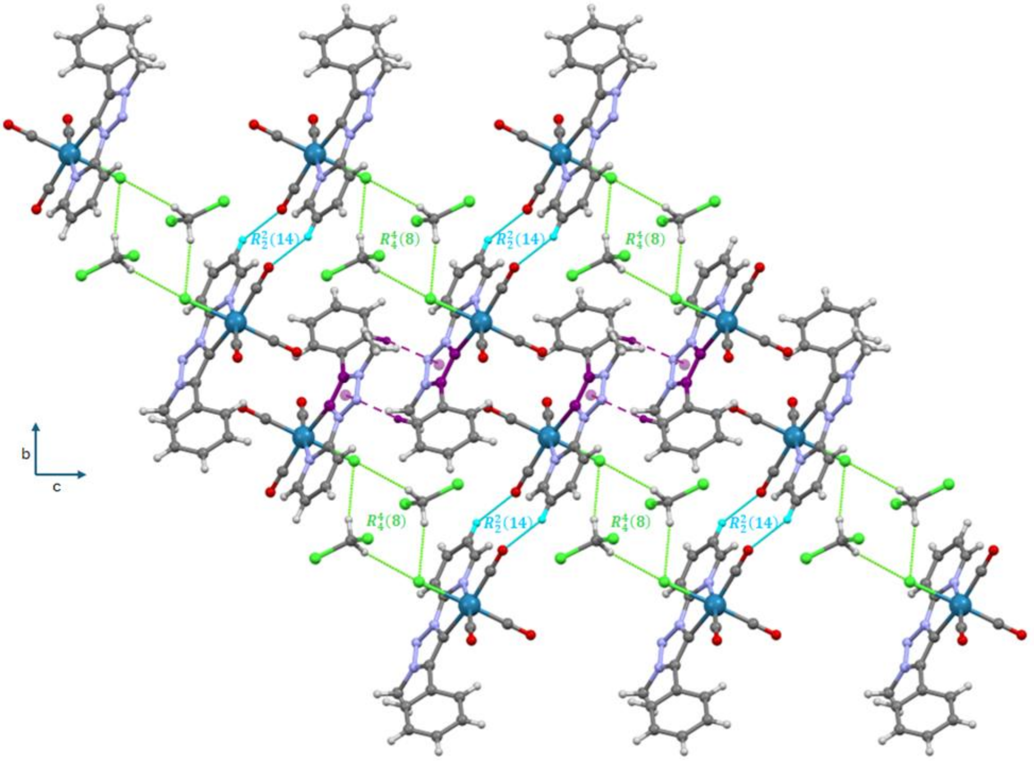

Figure S28. (a) $R_4^4(8)$ tetramers formed *via* intermolecular C18–H18A$_{(DCM)}\cdots$Cl1 and C18–H18B$_{(DCM)}\cdots$Cl1 (neon green color) interactions of two molecules of **Re-T-Tapy-Me** and two molecules of DCM. (b) $R_2^2(14)$ dimers formed *via* two intermolecular C5–H5$_{(py)}\cdots$O3$_{(CO)}$ (cyan color) interactions of two antiparallel molecules of **Re-T-Tapy-Me**. (c) Weak intermolecular C16–H16$_{(Ph)}\cdots\pi_{(trz)}$ (tyrian purple color) interactions stabilizing the structure.

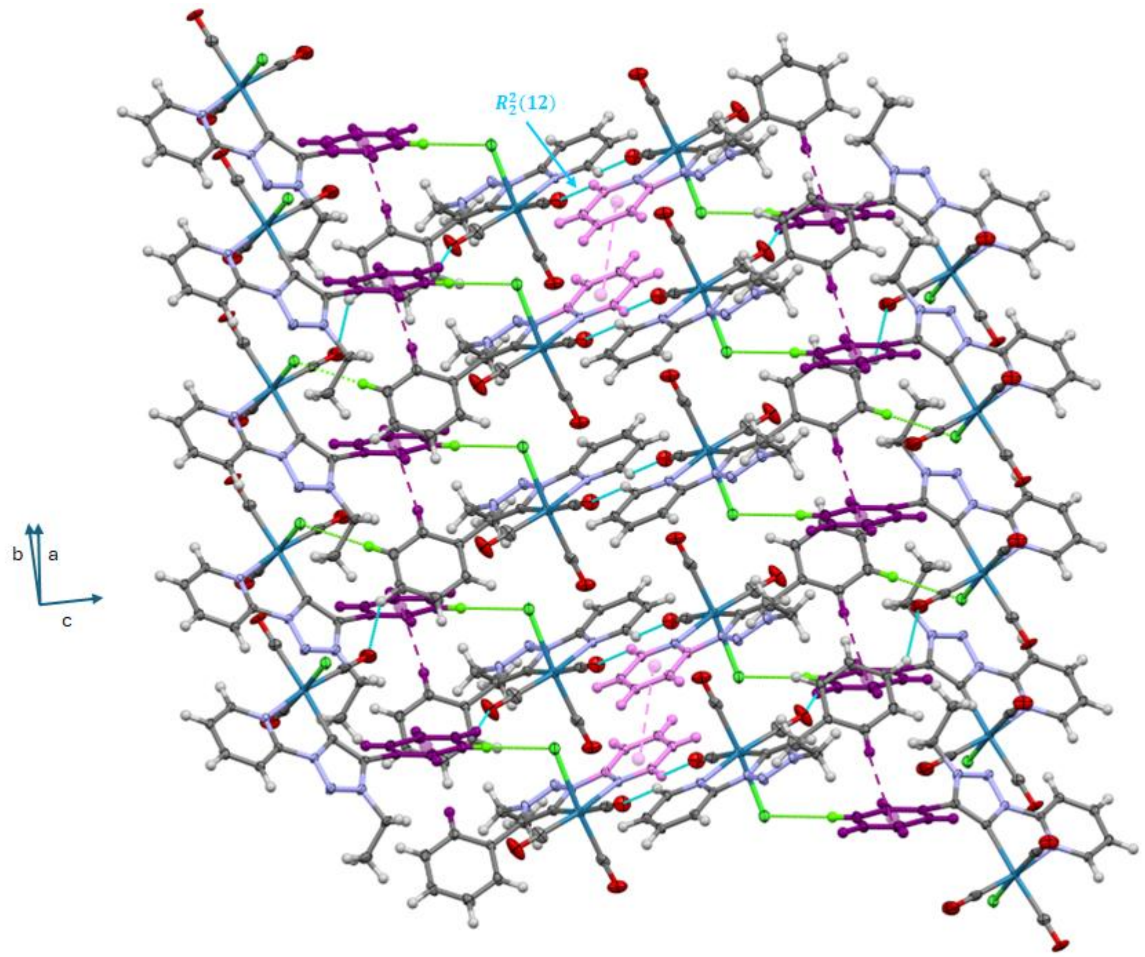

Figure S29. (a) $R_2^2(12)$ dimers formed *via* two intermolecular C4–H4$_{(py)}\cdots$O3$_{(CO)}$ (cyan color) interactions of two antiparallel molecules of **Re-T-Tapy-Et**. (b) $\pi_{(py)}\cdots\pi_{(py)}$ (violet color) stacking interactions and (c) weak intermolecular C16–H16$\cdots\pi_{(Ph)}$ (tyrian purple color) interactions stabilizing the structure of **Re-T-Tapy-Et**.

3. Hirshfeld surface (HS) analysis.

The HS mapping of curvedness and shape index is used to identify the presence of π–π stacking interactions. Flat regions and edges in the curvedness surface are indicated as green and blue areas, respectively. Low curvedness indicates the presence of π–π stacking in the crystal, whereas a high curvedness suggests an absence of π–π stacking. Hydrogen donor and acceptor groups in the shape index are represented as blue (bumps) or red (hollows) regions, respectively.

The intensity of the d_{norm} point provides a simple visual information about important regions of intermolecular interaction through color mapping. Intermolecular contacts shorter than the sum of the van der Waals radii (d_{norm}<0) of the interacting atoms are denoted as red spots on the surfaces, whereas longer than the sum of the van der Waals radii (d_{norm}>0) of the interacting atoms are represented by blue regions. The van der Waals contacts (d_{norm}=0) are coloured white. 2D fingerprint plots provide correlation between d_i and d_e.

	**Re-Tapy**	**Re-T-Tapy-Me**	**Re-T-Tapy-Et**
d_{norm}	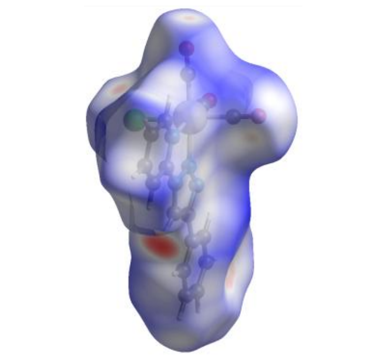	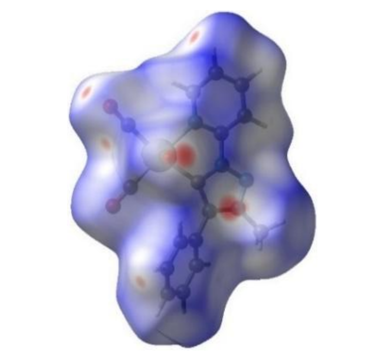	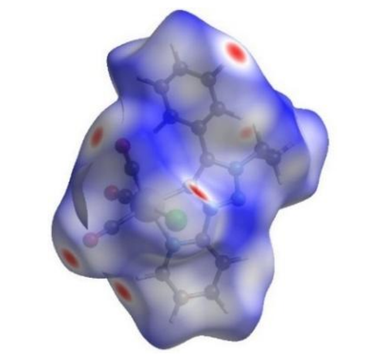
d_i	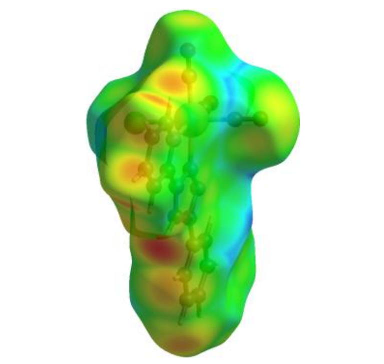	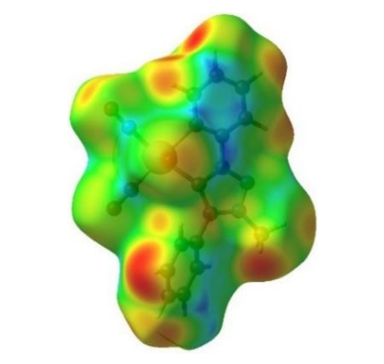	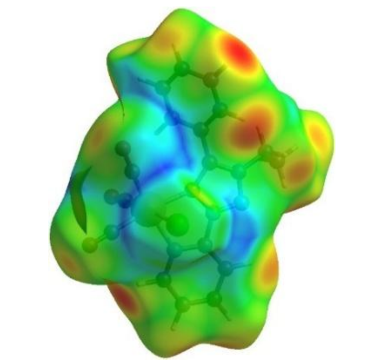
d_e	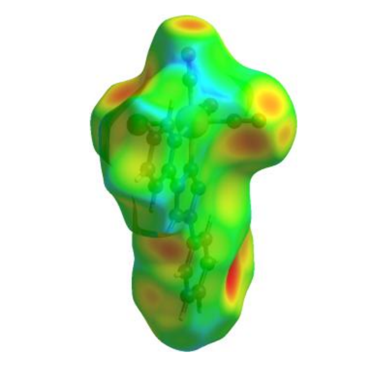	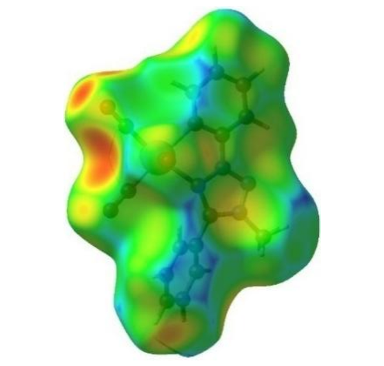	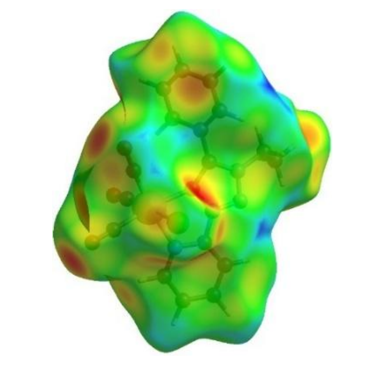

Figure S30. HS plotted over d_{norm}, d_i, d_e of **Re-Tapy**, **Re-T-Tapy-Me**, and **Re-T-Tapy-Et**.

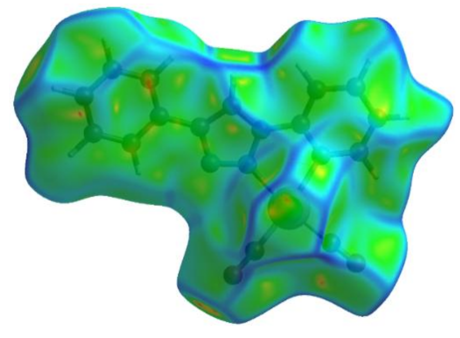 **Re-Tapy**	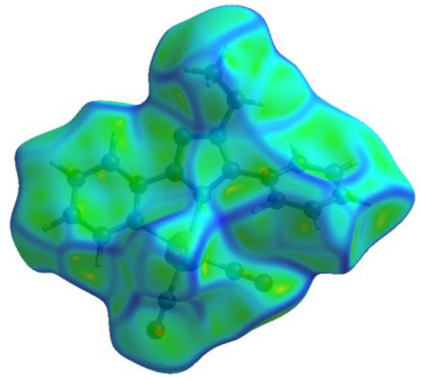 **Re-T-Tapy-Et**

Figure S31. HS plotted over curvedness and shape index of **Re-Tapy** and **Re-T-Tapy-Et**.

Analysis of the HS of Re-Tapy.

HS of **Re-Tapy** showed a surface having an area of 367.20 Å^2, which is spread over volume of 412.62 Å^3, in which the surface is generated between –0.5174 a.u. (red spot) and 1.4123 a.u. (blue colour) for d_{norm}, 0.8236 a.u. and 2.8763 a.u. for d_{i}, 0.8383 a.u. and 2.8409 a.u. for d_{e} (Fig. S31). The curvedness and shape index plots are generated from –3.6186 to 0.3477 for curvedness and from –0.9927 to 0.9992 a.u. for SI (Fig. S32 and Fig.3a). 2D Fingerprint plots of Hirshfeld surface for **Re-Tapy** with relative contributions of different interactions are shown in Fig. S33. The presence of the short contacts C7–H7$_{\text{(py)}}\cdots$O4$_{\text{(DMSO)}}$, C9–H9$_{\text{(trz)}}\cdots$O4$_{\text{(DMSO)}}$ and C18–H18A$_{\text{(DMSO)}}\cdots$Cl1 and [O···H/H···O = 32.7%, Cl···H/H···Cl = 11.4%, N···H/H···N = 7.7%] between the neighboring molecules are detected in the 2D FP of **Re-Tapy**. These interactions are viewed on the d_{norm} surface by light and bright red spots, respectively (Fig. S31). The contribution of the C···C and C···N/N···C short contacts by 5.6% and 2.9% in the FP plots, along with the green flat area and red/blue triangles in the curvedness and shape index maps, respectively, confirm the existence of $\pi_{\text{(Ph)}}\cdots\pi_{\text{(py)}}$ stacking interactions between the parallel phenyl and pyridine rings of the neighboring ligands (Fig. S3a). In addition, C17–H17$_{\text{(DMSO)}}\cdots\pi_{\text{(Ph)}}$ [C···H/H···C = 16.7%, N···H/H···N = 7.7%] and various other interactions [H···H = 16.8%, C···O/O···C = 2.4%] stabilizing the structure of **Re-Tapy** are also found in the 2D FP.

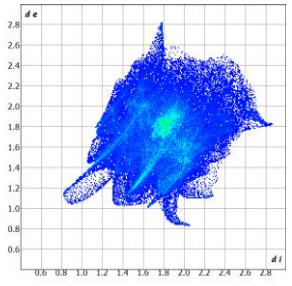	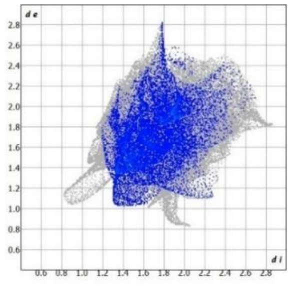	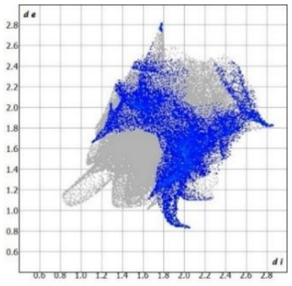
**Overall interactions**	**H···H = 16.8%**	**C···H = 16.7%**
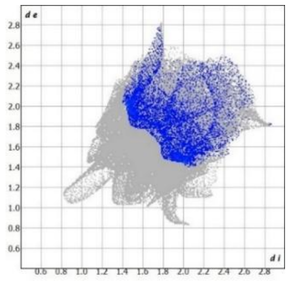	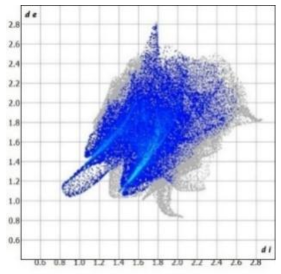	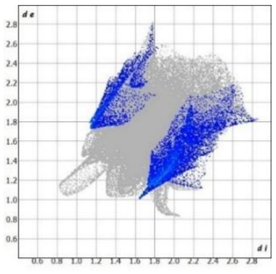
**N···H = 7.7%**	**O···H = 32.7%**	**Cl···H = 11.4%**
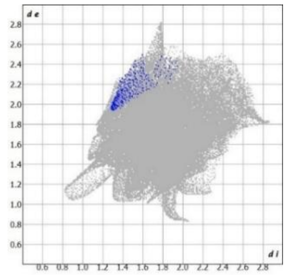	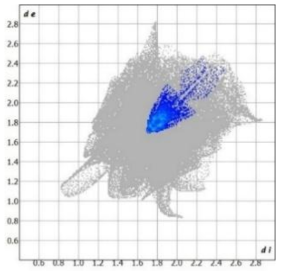	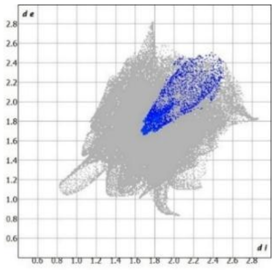
**S···H = 0.3%**	**C···C = 5.6%**	**C···N = 2.9%**

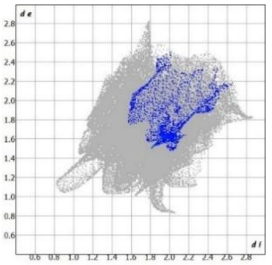	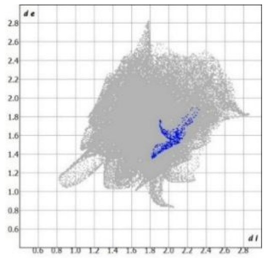	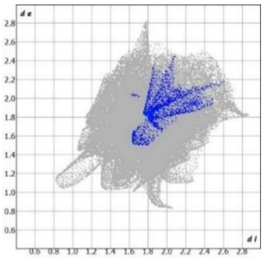
**C···O = 2.4%**	**C···S = 0.6%**	**O···O = 2.2%**
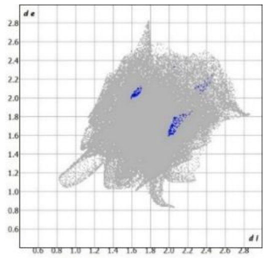	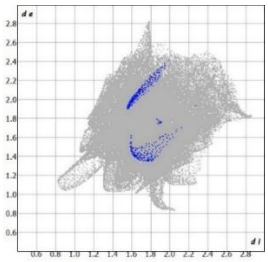	
**O···N = 0.2%**	**O···S = 0.5%**	

Figure S32. Two-dimensional fingerprint plots for overall interactions and individual interactions in crystal packing of **Re-Tapy**.

Analysis of the HS of Re-T-Tapy-Me.

The HS analysis reveals that **Re-T-Tapy-Me** is covering an area of 370.99 Å^2 and a volume of 438.26 Å^3. The colour scale on the HS ranges from –0.2250 a.u. (red spot) and 1.3776 a.u. (blue colour), 1.0720 a.u. and 2.7730 a.u. for d_i, 0.9585 a.u. and 2.7913 a.u. for d_e (Fig. S31). 2D Fingerprint plots of Hirshfeld surface for **Re-T-Tapy-Me** with relative contributions of different interactions are shown in Fig. S34. Weak intermolecular interactions C5–H5$_{(py)}$···O3$_{(CO)}$, C18–H18A$_{(DCM)}$···Cl1 and C18–H18B$_{(DCM)}$···Cl1 [O···H/H···O = 21.3%, Cl···H/H···Cl = 18.7%], through which the adjacent molecules interact are detected in 2D FP. The curvedness map showed no green flat areas, and the shape index map has no complementary red/blue triangles, which are distinct for the identification of $\pi\cdots\pi$ stacking. **Re-T-Tapy-Me** is further stabilized by C16–H16$_{(Ph)}\cdots\pi_{(trz)}$ [C···H/H···C = 17.0%, N···H/H···N = 5.9%] and various other interactions [H···H = 19.1%, C···O/O···C = 4.6%].

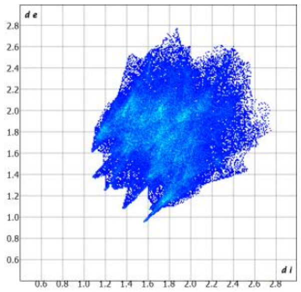	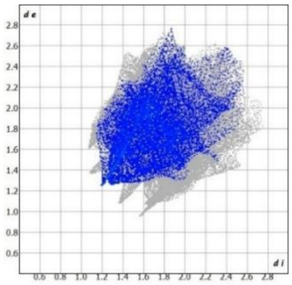	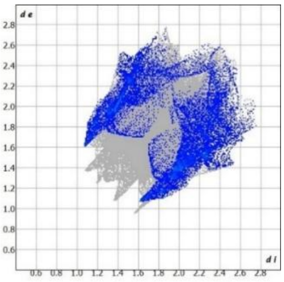
**Overall interactions**	**H···H = 19.1%**	**C···H = 17.0%**

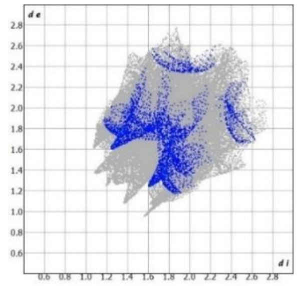	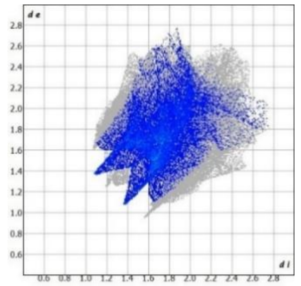	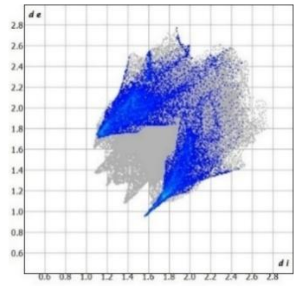
**N···H = 5.9%**	**O···H = 21.3%**	**Cl···H = 18.7%**
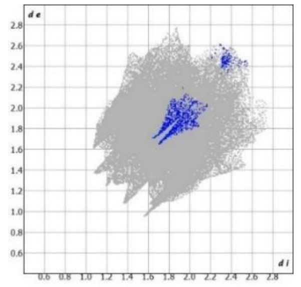	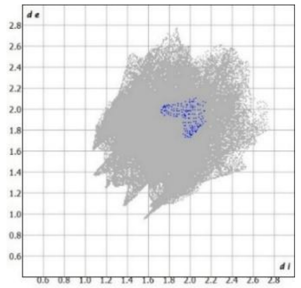	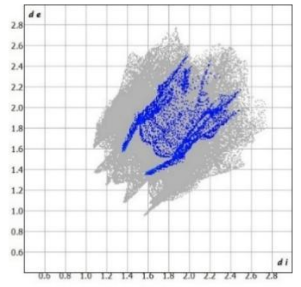
**C···C = 1.1%**	**C···N = 0.2%**	**C···O = 4.6%**
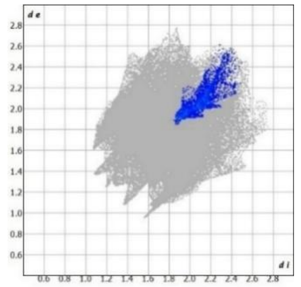	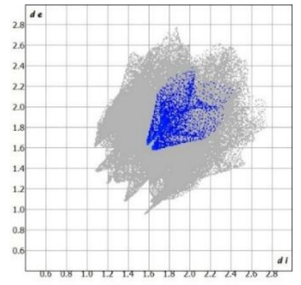	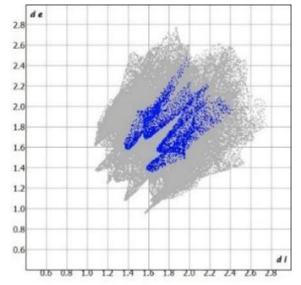
**C···Cl = 3.7%**	**O···O = 3.0%**	**O···N = 3.9%**
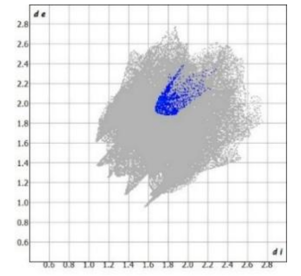	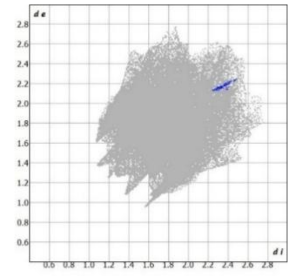	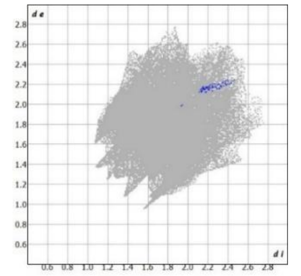
**O···Cl = 1.2%**	**N···Cl = 0.1%**	**Cl···Cl = 0.1%**

Figure S33. Two-dimensional fingerprint plots for overall interactions and individual interactions in crystal packing of **Re-T-Tapy-Me**.

Analysis of the HS of Re-T-Tapy-Et.

In **Re-T-Tapy-Et**, the HS spread over an area of 387.98 Å^2 and volume of 458.62 Å^3. The colour scale on the HS ranges from –0.2491 a.u. (red spot) and 1.3483 a.u. (blue colour), 0.9487 a.u. and 2.7563 a.u. for d_i, 0.9496 a.u. and 2.7397 a.u. for d_e (Fig. S31). The curvedness and shape index plots are generated from –4.0988 a.u. and 0.2873 a.u. for curvedness and from –0.9949 to 0.9983 a.u. for SI (Fig. S32 and 3a). 2D Fingerprint plots of Hirshfeld surface for **Re-T-Tapy-Et** with relative contributions of different interactions are shown in Fig. S35. Weak intermolecular interactions C4–H4$_{(py)}$···O3$_{(CO)}$, C14–

H14$_{(Ph)}$···O2$_{(CO)}$ and C15–H15$_{(Ph)}$···Cl1 [O···H/H···O = 30.3%, Cl···H/H···Cl = 12.2%] which are linking the adjacent molecules of **Re-T-Tapy-Et** are found in 2D FP. The contribution of the C···C and C···N/N···C short contacts by 3.3% and 2.2% in the FP plots, along with the green flat area and red/blue triangles in the curvedness and shape index maps, respectively, confirm the existence of $\pi_{(py)}\cdots\pi_{(py)}$ stacking interactions between the parallel pyridine rings of the neighboring ligands (Fig. S32 and 3a). **Re-T-Tapy-Et** is further stabilized by C16–H16···$\pi_{(Ph)}$ [C···H/H···C = 18.7%, N···H/H···N = 4.8%] and various other interactions [H···H = 24.0%, C···O/O···C = 0.8%].

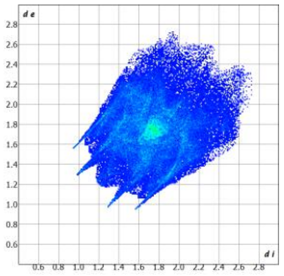	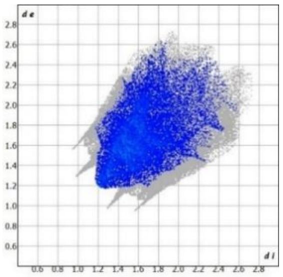	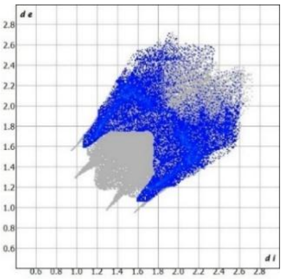
**Overall interactions**	**H···H = 24.0%**	**C···H = 18.7%**
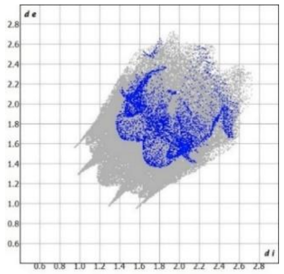	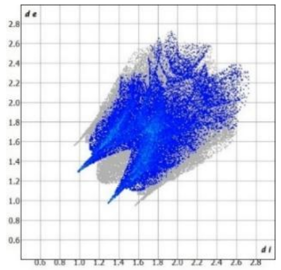	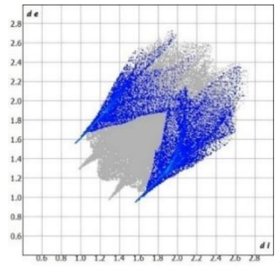
**N···H = 4.8%**	**O···H = 30.3%**	**Cl···H = 12.2%**
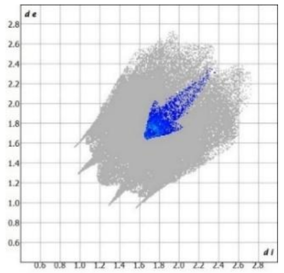	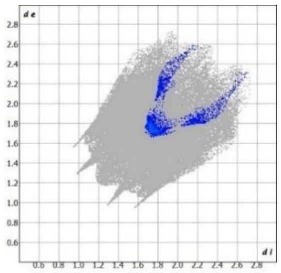	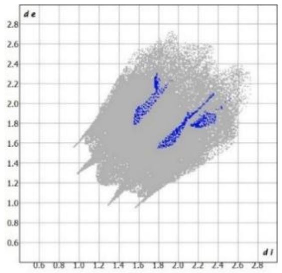
**C···C = 3.3%**	**C···N = 2.2%**	**C···O = 0.8%**
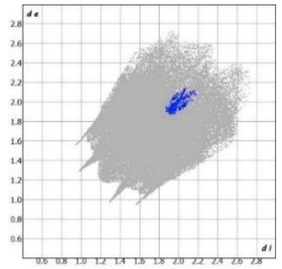	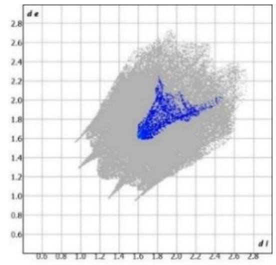	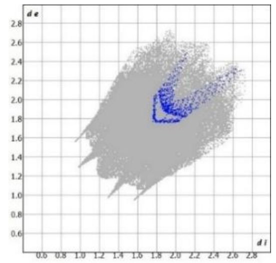
**C···Cl = 0.8%**	**O···O = 2.1%**	**O···N = 0.8%**

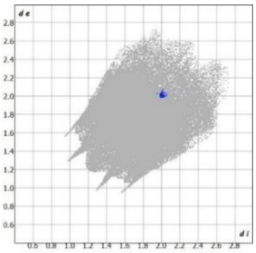		
**N···Cl = 0.1%**		

Figure S34. Two-dimensional fingerprint plots for overall interactions and individual interactions in crystal packing of **Re-T-Tapy-Et**.

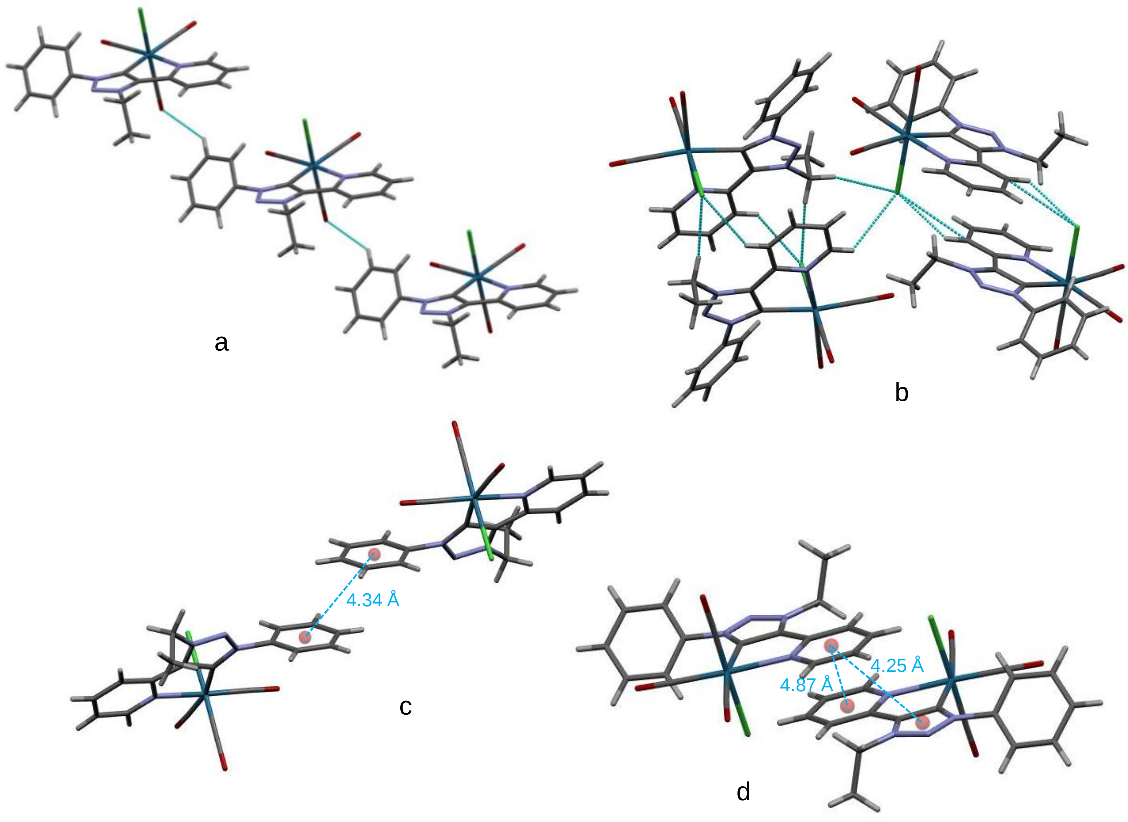

Figure S35. Molecular arrangement of **Re-T-Pyta$_{(1,2,3)}$-Et**. a) Intermolecular C-H···O interactions induce the formation of an infinite one-dimensional chain. b) Stabilization of the 3D structure by C-H···Cl intermolecular interactions. c) Intermolecular distances between centroids, showing the absence of $\pi_{(Ph)}\cdots\pi_{(Ph)}$ interactions. d) Intermolecular distances between centroids, showing the absence of $\pi_{(py)}\cdots\pi_{(py)}$ interactions.

DFT and TD-DFT Calculations

Table S5. Selected calculated bond lengths [Å] and angles [°] in the ground (S_0) first singlet excited (S_1) and first triplet excited (T_1) states for **Re-Tapy**, together with the experimental data.

Bond lengths	Exp.	Optimized			Bond angles	Exp.	Optimized		
		S_0	S_1	T_1			S_0	S_1	T_1
Re(1)-C(1)	1.924(9)	1.900	1.956	1.940	C(2)-Re(1)-C(1)	88.3(4)	89.90	90.13	87.95
Re(1)-C(2)	1.918(6)	1.914	1.951	1.982	C(2)-Re(1)-C(3)	89.3(3)	89.07	84.65	90.22
Re(1)-C(3)	1.926(8)	1.913	1.950	1.926	C(1)-Re(1)-C(3)	90.1(3)	89.90	92.40	90.25
Re(1)-N(1)	2.194(6)	2.202	2.153	2.174	C(2)-Re(1)-N(1)	99.0(3)	99.51	101.52	98.52
Re(1)-N(3)	2.144(6)	2.152	2.108	2.034	C(1)-Re(1)-N(1)	95.9(3)	93.91	89.02	94.37
Re(1)-Cl(1)	2.4715(18)	2.506	2.399	2.456	C(3)-Re(1)-N(1)	169.9(3)	170.60	173.68	170.23
					C(2)-Re(1)-N(3)	172.8(4)	172.01	176.35	174.12
C(1)-O(1)	1.134(10)	1.155	1.139	1.144	C(1)-Re(1)-N(3)	94.1(3)	94.20	91.04	91.77
C(2)-O(2)	1.140(8)	1.151	1.143	1.140	C(3)-Re(1)-N(3)	97.5(3)	97.78	98.76	95.66
C(3)-O(3)	1.145(10)	1.150	1.141	1.146	N(1)-Re(1)-N(3)	74.0(2)	73.39	75.04	75.64
					C(2)-Re(1)-Cl(1)	93.8(4)	92.06	90.93	86.47
					C(1)-Re(1)-Cl(1)	177.4(2)	176.80	175.49	174.20
					C(3)-Re(1)-Cl(1)	91.4(2)	92.66	92.06	91.38
					N(1)-Re(1)-Cl(1)	82.28(17)	83.28	86.47	84.87
					N(3)-Re(1)-Cl(1)	83.66(15)	83.56	87.65	93.61
					O(1)-C(1)-Re(1)	179.5(8)	179.43	179.46	179.01
					O(2)-C(2)-Re(1)	178.9(10)	179.37	178.96	179.28
					O(3)-C(3)-Re(1)	177.3(7)	178.77	179.58	178.37

Table S6. Selected calculated bond lengths [Å] and angles [°] in the ground (S_0) first singlet excited (S_1) and first triplet excited (T_1) states for **Re-T-Tapy-Me**, together with the experimental data.

Bond lengths	Exp.	Optimized			Bond angles	Exp.	Optimized		
		S_0	S_1	T_1			S_0	S_1	T_1
Re(1)-C(1)	1.924(2)	1.907	1.932	1.923	C(2)-Re(1)-C(1)	87.42(9)	90.12	91.88	90.38
Re(1)-C(2)	1.905(2)	1.893	1.947	1.941	C(2)-Re(1)-C(3)	90.39(10)	91.16	90.81	87.61
Re(1)-C(3)	1.942(2)	1.947	1.993	2.015	C(1)-Re(1)-C(3)	91.22(9)	89.73	86.23	91.64
Re(1)-C(9)	2.1480(19)	2.141	2.134	2.080	C(2)-Re(1)-C(9)	94.69(9)	93.66	85.22	85.40
Re(1)-N(1)	2.2133(17)	2.224	2.134	2.193	C(1)-Re(1)-C(9)	98.63(8)	98.56	99.91	97.73
Re(1)-Cl(1)	2.5008(5)	2.531	2.400	2.438	C(3)-Re(1)-C(9)	169.10(8)	170.39	172.78	168.33
					C(2)-Re(1)-N(1)	96.37(8)	94.58	91.26	95.15
C(1)-O(1)	1.148(3)	1.153	1.146	1.148	C(1)-Re(1)-N(1)	172.97(7)	172.02	174.78	171.51
C(2)-O(2)	1.147(3)	1.158	1.142	1.144	C(3)-Re(1)-N(1)	94.66(8)	96.62	97.90	95.02
C(3)-O(3)	1.149(3)	1.151	1.141	1.139	C(9)-Re(1)-N(1)	75.21(7)	74.73	76.19	76.35
					C(2)-Re(1)-Cl(1)	178.08(7)	177.20	174.67	177.12
					C(1)-Re(1)-Cl(1)	93.60(6)	92.21	90.77	90.27
					C(3)-Re(1)-Cl(1)	91.21(6)	90.41	93.98	89.57
					C(9)-Re(1)-Cl(1)	83.56(5)	177.20	89.77	97.29
					N(1)-Re(1)-Cl(1)	82.44(4)	92.21	85.77	84.53
					O(1)-C(1)-Re(1)	178.55(19)	179.25	178.96	178.54
					O(2)-C(2)-Re(1)	177.3(2)	179.58	178.92	179.12
					O(3)-C(3)-Re(1)	178.08(19)	179.60	177.28	177.60

Table S7. Torsion (dihedral) angle value between pyta and R calculated using the density functional theory (DFT) method at the PBE1PBE/LANL2DZ level for the ground state (S_0) and first singlet excited (S_1) and first triplet excited state (T_1) of **Re-Tapy** and **Re-T-Tapy-Me** obtained from crystallographic data.

Complex	Bond angle [°]	SXRD	S_0	S_1	T_1
**Re-Tapy**	N(4)–C(10)–C(11)–C(16)	3.85	13.68	4.79	–0.06
**Re-T-Tapy-Me**	C(9)–C(10)–C(11)–C(12)	–43.28	–52.81	–57.56	–56.62

Table S8. The frontier molecular orbital compositions (%) and energy levels for **Re-Tapy** (in dichloromethane).

Orbital		Energy (eV)	MO Contribution (%)					Main bond type
			Re	CO	Cl	pytrz	R	
96	L+5	–0.65	5	5	0	18	71	π*(R)/π*(tapy)
95	L+4	–0.71	33	50	-1	13	6	p(Re)+π*(CO)
94	L+3	–0.89	30	65	0	3	3	p(Re)+π*(CO)
93	L+2	–1.32	1	3	0	37	59	π*(R)/π*(tapy)
92	L+1	–1.87	0	1	0	95	3	π*(tapy)
91	L	–2.59	3	5	0	91	2	π*(tapy)
HOMO–LUMO gap **(E = 4.03 eV)**								
90	H	–6.62	46	23	19	8	5	d(Re)+π(CO)/π(Cl)
89	H–1	–6.74	47	22	23	6	2	d(Re)+π(CO)/π(Cl)
88	H–2	–7.21	8	3	10	23	56	π(R)/π(tapy)
87	H–3	–7.29	63	29	2	1	4	d(Re)+π(CO)
86	H–4	–7.69	0	0	0	1	99	π(R)
85	H–5	–8.19	12	4	55	29	1	π(Cl)/π(tapy)/d(Re)
84	H–6	–8.24	15	5	61	17	2	π(Cl)/π(tapy)/d(Re)
83	H–7	–8.39	4	1	13	68	13	π(tapy)/π(R)
82	H–8	–8.88	8	15	70	6	1	π(Cl)
81	H–9	–9.33	1	4	1	89	6	π(tapy)
80	H–10	–9.48	4	2	5	70	18	π(tapy)/π(R)

R = $–C_6H_5$

Table S9. The frontier molecular orbital compositions (%) and energy levels for **Re-T-Tapy-Me** (in dichloromethane).

Orbital		Energy (eV)	MO Contribution (%)					Main bond type
			Re	CO	Cl	pytrz	R	
100	L+5	–0.41	31	52	-2	7	13	p(Re)+π*(CO)
99	L+4	–0.64	21	25	0	26	28	p(Re)+π*(CO)/π*(L)
98	L+3	–0.75	15	31	0	7	47	p(Re)+π*(CO)
97	L+2	–1.18	5	7	0	36	53	π*(R)/π*(trzpy)
96	L+1	–1.80	1	2	0	93	4	π*(trzpy)
95	L	–2.43	3	3	0	94	0	π*(trzpy)
HOMO–LUMO gap **(E = 3.92 eV)**								
94	H	–6.35	49	23	18	7	3	d(Re)+π(CO)/π(Cl)
93	H–1	–6.50	50	24	21	4	0	d(Re)+π(CO)/π(Cl)
92	H–2	–6.90	68	30	1	1	1	d(Re)+π(CO)
91	H–3	–7.37	1	0	24	28	47	π(R)/π(trzpy)/π(Cl)
90	H–4	–7.72	0	0	0	1	99	π(R)
89	H–5	–7.91	6	4	39	32	19	π(Cl)/π(trzpy)/π(R)
88	H–6	–8.00	13	4	67	11	5	π(Cl)/π(trzpy)/d(Re)
87	H–7	–8.20	3	2	7	75	13	π(trzpy)/π(R)

86	H–8	–8.62	9	13	61	12	5	π(Cl)
85	H–9	–8.94	7	7	16	62	9	π(trzpy)
84	H–10	–9.46	2	13	9	72	4	π(trzpy)/π(R)

R = $–C_6H_5$

Table S10. The main electronic transitions for **Re-Tapy**, calculated with TDDFT method at the PBE1PBE/LANL2DZ level (in dichloromethane).

Electronic transition	Contribution	Assignment		E_{calc} /eV	$λ_{calc}$ /nm	*f*	$λ_{exp}$ /nm
$S_0→S_1$	H→L	d(Re)+π(CO)/π(Cl)→π*(tapy)	MLCT/LLCT	3.05	406.4	*0.0051*	
$S_0→S_2$	H–1→L	d(Re)+π(CO)/π(Cl)→π*(tapy)	MLCT/LLCT	3.24	382.6	*0.0984*	
$S_0→S_4$	H–2→L	π(R)/π(tapy)→π*(tapy)	ILCT	3.89	318.6	*0.0510*	
$S_0→S_{12}$	H→L+2	d(Re)+π(CO)/π(Cl)→π*(R)/π*(tapy)	MLCT/LLCT	4.62	268.2	*0.1124*	
$S_0→S_{13}$	H–5→L	π(Cl)/π(tapy)/d(Re)→π*(tapy)	LLCT/ILCT	4.64	267.4	*0.1782*	
	H–2→L+1	π(R)/π(tapy)→π*(tapy)	ILCT				
$S_0→S_{15}$	H–6→L	π(Cl)/π(tapy)/d(Re)→π*(tapy)	LLCT/ILCT	4.70	263.8	*0.0650*	
$S_0→S_{16}$	H–5→L	π(Cl)/π(tapy)/d(Re)→π*(tapy)	LLCT/ILCT	4.74	261.7	*0.1641*	
$S_0→S_{17}$	H–1→L+4	d(Re)+π(CO)/π(Cl)→p(Re)+π*(CO)	MLCT/LLCT	4.88	253.8	*0.1131*	
$S_0→S_{18}$	H–7→L	π(tapy)/π(R)→π*(tapy)	ILCT	4.90	252.9	*0.2760*	
$S_0→S_{21}$	H–3→L+3	d(Re)+π(CO)→p(Re)+π*(CO)	MLCT/ILCT	5.09	243.5	*0.1550*	
$S_0→S_{22}$	H–2→L+2	π(R)/π(tapy)→π*(R)/π*(tapy)	ILCT	5.19	238.9	*0.1904*	
$S_0→S_{23}$	H→L+5	d(Re)+π(CO)/π(Cl)→π*(R)/π*(tapy)	MLCT/LLCT	5.28	234.7	*0.0522*	
$S_0→S_{29}$	H–5→L+1	π(Cl)/π(tapy)/d(Re)→π*(tapy)	LLCT/ILCT	5.48	226.3	*0.1285*	
$S_0→S_{36}$	H–7→L+1	π(tapy)/π(R)→π*(tapy)	ILCT	5.71	217.2	*0.0508*	
$S_0→S_{43}$	H–2→L+5	π(R)/π(tapy)→π*(R)/π*(tapy)	ILCT	5.98	207.2	*0.0601*	
	H→L+7	d(Re)+π(CO)/π(Cl)→p(Re)+π*(CO)/π*(L)	MLCT/LLCT				
$S_0→S_{49}$	H–6→L+2	π(Cl)/π(tapy)/d(Re)→π*(R)/π*(tapy)	LLCT/ILCT	6.15	201.5	*0.0708*	

MLCT: metal-to-ligand charge transfer; LMCT: ligand-to-metal charge transfer; LLCT: ligand-to-ligand charge transfer; ILCT: intraligand charge transfer.

Table S11. The main electronic transitions for **Re-T-Tapy-Me**, calculated with TDDFT method at the PBE1PBE/LANL2DZ level (in dichloromethane).

Electronic transition	Contribution	Assignment		E_{calc} /eV	$λ_{calc}$ /nm	*f*	$λ_{exp}$ /nm
$S_0→S_1$	H→L	d(Re)+π(CO)/π(Cl)→π*(trzpy)	MLCT/LLCT	2.99	415.0	*0.0085*	
$S_0→S_2$	H–1→L	d(Re)+π(CO)/π(Cl)→π*(trzpy)	MLCT/LLCT	3.18	389.8	*0.0665*	
$S_0→S_7$	H–3→L	π(R)/π(trzpy)/π(Cl)→π*(trzpy)	ILCT/LLCT	4.18	296.8	*0.0491*	
$S_0→S_{17}$	H–3→L+1	π(R)/π(trzpy)/π(Cl)→π*(trzpy)	ILCT/LLCT	4.82	257.0	*0.2063*	
$S_0→S_{19}$	H–7→L	π(trzpy)/π(R)→π*(trzpy)	ILCT	4.91	252.4	*0.0947*	
$S_0→S_{25}$	H–5→L+1	π(Cl)/π(trzpy)/π(R)→π*(trzpy)	LLCT/ILCT	5.26	235.9	*0.0793*	
	H→L+6	d(Re)+π(CO)/π(Cl)→π*(R)/π*(trzpy)	MLCT/LLCT				
$S_0→S_{27}$	H–8→L	π(Cl)→π*(trzpy)	LLCT	5.34	232.2	*0.0760*	
$S_0→S_{28}$	H–3→L+2	π(R)/π(trzpy)/π(Cl)→π*(R)/π*(trzpy)	ILCT/LLCT	5.35	231.7	*0.0930*	
$S_0→S_{29}$	H–3→L+2	π(R)/π(trzpy)/π(Cl)→π*(R)/π*(trzpy)	ILCT/LLCT	5.38	230.4	*0.0972*	
	H–6→L+1	π(Cl)/π(trzpy)/d(Re)→π*(trzpy)	LLCT/ILCT				
	H–8→L	π(Cl)→π*(trzpy)	LLCT				
$S_0→S_{32}$	H–2→L+8	d(Re)+π(CO)→p(Re)+π*(CO)/π*(L)	MLCT/LLCT	5.44	227.8	*0.0635*	
$S_0→S_{33}$	H–4→L+1	π(R)→π*(trzpy)	ILCT	5.50	225.6	*0.0472*	
$S_0→S_{39}$	H–2→L+6	d(Re)+π(CO)→π*(R)/π*(trzpy)	MLCT/LLCT	5.71	217.3	*0.0470*	
$S_0→S_{55}$	H–3→L+5	π(R)/π(trzpy)/π(Cl)→π*(R)/π*(trzpy)	ILCT/LLCT	6.14	201.9	*0.0453*	

MLCT: metal-to-ligand charge transfer; LMCT: ligand-to-metal charge transfer; LLCT: ligand-to-ligand charge transfer; ILCT: intraligand charge transfer.

Table S12. Excitation energies and oscillator strengths calculated on the optimized geometry of the first singlet excited state (S_1) of complexes **Re-Tapy** and **Re-T-Tapy-Me** (in dichloromethane) with TDDFT method at the PBE1PBE/LANL2DZ level.

State	Contribution	Assignment		E_{calc} /eV	λ_{calc} /nm	f
		**Re- Tapy**				
1	HOMO→LUMO	d(Re)+π(CO)/π(Cl)→π*(tapy)	MLCT/LLCT	2.36	525.1	*0.0074*
2	H–1→LUMO	d(Re)+π(CO)/π(Cl)→π*(tapy)	MLCT/LLCT	2.72	456.3	*0.1475*
3	H–2→LUMO	π(R)/π(tapy)→π*(tapy)	ILCT	3.13	395.7	*0.0009*
4	HOMO→L+1	d(Re)+π(CO)/π(Cl)→π*(tapy)	MLCT/LLCT	3.55	349.1	*0.0473*
		**Re-T-Tapy-Me**				
1	HOMO→LUMO	d(Re)+π(CO)/π(Cl)→π*(trzpy)	MLCT/LLCT	2.20	563.2	*0.0103*
2	H–1→LUMO	d(Re)+π(CO)/π(Cl)→π*(trzpy)	MLCT/LLCT	2.57	483.0	*0.0841*
3	H–2→LUMO	d(Re)+π(CO)→π*(rzpy)	MLCT/LLCT	2.93	423.1	*0.0001*
4	HOMO→L+1	d(Re)+π(CO)/π(Cl)→π*(trzpy)	MLCT/LLCT	3.26	380.9	*0.0232*

Table S13. Phosphorescence emission energies of **Re-Tapy** and **Re-T-Tapy-Me** calculated with DFT and TDDFT methods at the PBE1PBE/LANL2DZ level, in comparison with the experimental values.

Complex	Exp.	DFT			TDDFT			
		ΔE_{T1}–S_0		Character	eV	nm	Major contribution	Character
	nm	eV	nm					
**Re-Tapy**	600	1.99	623.0	^{3}MLCT/^{3}LLCT	1.90	653.6	H→L	^{3}MLCT/^{3}LLCT
**Re-T-Tapy-Me**	614	1.80	688.8	^{3}MLCT/^{3}LLCT	1.78	697.0	H→L	^{3}MLCT/^{3}LLCT

ΔE_{T1}–S_0 is the energy difference between the ground singlet and triplet states.

Table S14. Natural populations of the $5d_{xy}$, $5d_{xz}$, $5d_{yz}$, $5d_{x^2-y^2}$ and $5d_{z^2}$ orbitals of the central atom in **Re-Tapy** and **Re-T-Tapy-Me**.

Orbital	Complex	
	**Re-Tapy**	**Re-T-Tapy-Me**
$5d_{xy}$	1.217	1.457
$5d_{xz}$	1.203	1.231
$5d_{yz}$	1.366	1.336
$5d_{x^2-y^2}$	1.499	1.256
$5d_{z^2}$	1.413	1.501

The population of $5d$ orbitals ($5d_{xy}$, $5d_{xz}$, $5d_{yz}$, $5d_{x^2-y^2}$ and $5d_{z^2}$) of the central atoms shows that in free Re (+1) state, the population of $5d_{xy}$, $5d_{xz}$ and $5d_{yz}$ orbitals are 2.0, 2.0 and 2.0 (e) and the other two ($5d_{x^2-y^2}$ and $5d_{z^2}$) orbitals remain vacant. On complex formation, some decrease in populations for the $5d_{xy}$, $5d_{xz}$ and $5d_{yz}$ orbital and some increase in the populations of $5d_{x^2-y^2}$ and $5d_{z^2}$ orbital can be observed in comparison to free Re (+1) state.

Table S15. Atomic charges from the Natural Population Analysis (NPA) for **Re-Tapy** and **Re-T-Tapy-Me**.

Atom	Complex	
	**Re-Tapy**	**Re-T-Tapy-Me**
Re(1)	–1.01	–1.18
C(1)	+0.74	+0.78
C(2)	+0.76	+0.75
C(3)	+0.78	+0.75
N(1)	–0.41	–0.40
N(3)/C(9)	+0.01	–0.11
Cl(1)	–0.45	–0.46
O(1)	–0.48	–0.49
O(2)	–0.47	–0.50
O(3)	–0.47	–0.48

Natural population analysis (NPA) calculates the charge that is transferred between the donor and acceptor moieties and this charge transfer indicates electrovalent bonding between the Re and ligand. The calculated charge on the rhenium atoms is –0.99 (e) in **Re-Tapy** and **Re-T-Tapy-Me** which is slightly lower than the formal charge of +1, as a result of charge donation from the N atoms of the ligand. The charge on the nitrogen atoms is smaller and less negative, indicating that there is higher electron density delocalization from the N atoms to Re atoms. The positively charged carbon atoms of the carbonyl ligands are found to accept as much as ~0.74/0.77/0.76 (e) from Re atoms, while the Re atoms donates charge 0.99 (e). The negatively charged nitrogen atoms N(1), N(2) and chlorine atom Cl(1) are found to donate as much as ~0.39 (e), –0.23 (e) and ~0.46 (e) to Re atoms, respectively.

Table S16. Absolute electronegativity, absolute hardness, dipole moment (μ), electrophilicity index (ω) and global softness (σ) of complexes **Re-Tapy** and **Re-T-Tapy-Me**.

Parameter	Complex	
	**Re-Tapy**	**Re-T-Tapy-Me**
Total Energy (Hartree)	–1153.6711642	–1192.9217412
E_{HOMO} (eV)	–6.62	–6.35
E_{LUMO} (eV)	–2.59	–2.43
Energy gap ΔE (eV)	4.03	3.92
Ionization Potential I	6.62	6.35
Electron Affinity A	2.59	2.43
Electronegativity χ (eV)	4.61	4.39
Chemical potential μ	–4.61	–4.39
Hardness η (eV)	2.02	1.96
Softness σ (eV)	0.50	0.51
Electrophilicity ω (D/eV)	5.26	4.92
Dipole moment μ (D)	6.35	5.98

The determination of HOMO and LUMO energies is very important in view of chemical reaction. The HOMO (Highest Occupied Molecular Orbitals) is the orbital that primarily acts as an electron donor and the LUMO (Lowest Unoccupied Molecular Orbital) is the orbital that largely acts as the electron acceptor. The gap between HOMO and LUMO reflects the chemical reactivity and stability of the molecule.

The frontier molecular orbital descriptors such as ionization potential (IP = $-E_{HOMO}$), electron affinity (EA = $-E_{LUMO}$), electronegativity (χ = (I + A)/2), chemical potential ($\mu = -\chi$), hardness (η = (I – A)/2), softness ($\sigma = 1/\eta$) and electrophilicity index ($\omega = \mu^2/2\eta$) were calculated according to Koopmans theorem [T. Koopmans, *Physica*, 1933, 1, 104.]. Dipole moment using the equation: $\mu = 2.54 \times (x^2 + y^2 + z^2)^{1/2}$.

Occupied orbitals			Unoccupied orbitals	
**HOMO / 90** (–6.62 eV)	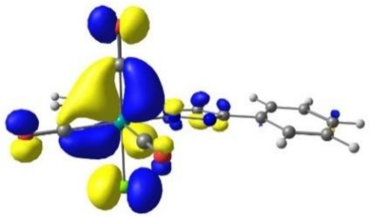		**LUMO / 91** (–2.59 eV)	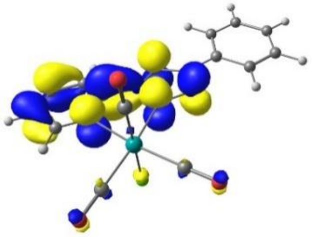
**HOMO–1 / 89** (–6.74 eV)	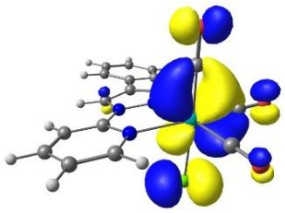		**LUMO+1 / 92** (–1.87 eV)	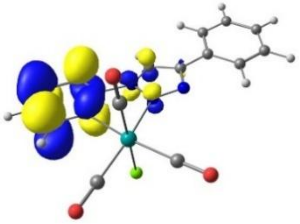
**HOMO–2 / 88** (–7.21 eV)	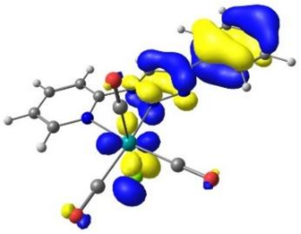		**LUMO+2 / 93** (–1.32 eV)	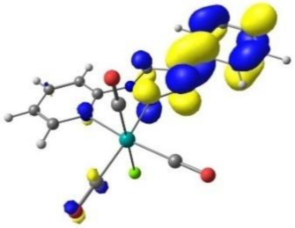
**HOMO–3 / 87** (–7.29 eV)	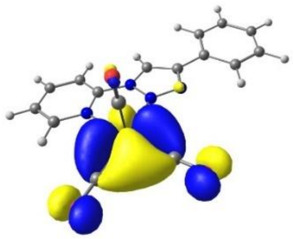		**LUMO+3 / 94** (–0.89 eV)	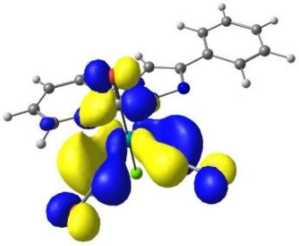
**HOMO–4 / 86** (–7.69 eV)	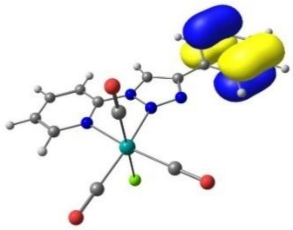		**LUMO+4 / 95** (–0.71 eV)	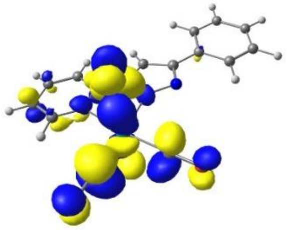
**HOMO–5 / 85** (–8.19 eV)	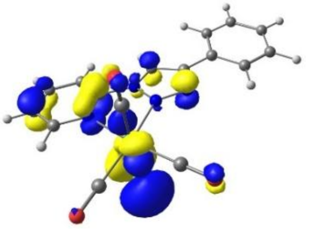		**LUMO+5 / 96** (–0.65 eV)	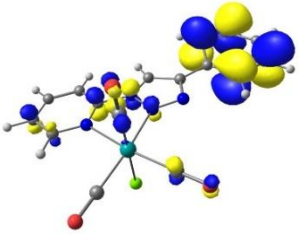

Figure S36. The isodensity plots of the frontier molecular orbitals of **Re-Tapy** (in dichloromethane).

Occupied orbitals			Unoccupied orbitals	
**HOMO / 94**<br>(–6.35 eV)	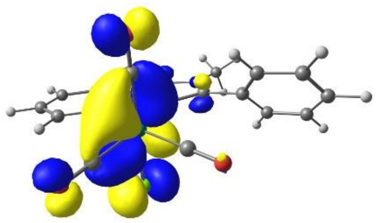		**LUMO / 95**<br>(–2.43 eV)	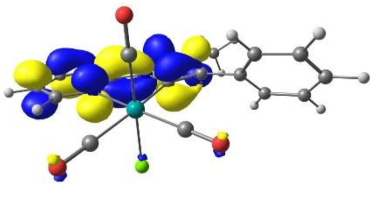
**HOMO–1 / 93**<br>(–6.50 eV)	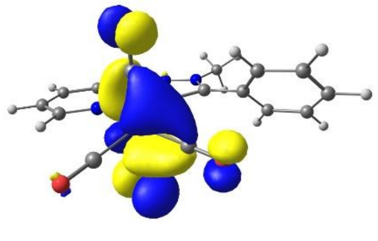		**LUMO+1 / 96**<br>(–1.80 eV)	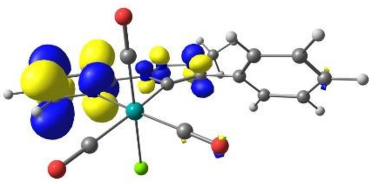
**HOMO–2 / 92**<br>(–6.90 eV)	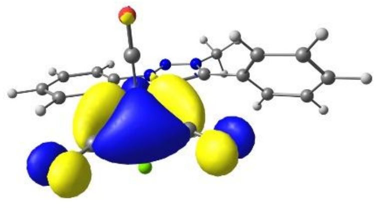		**LUMO+2 / 97**<br>(–1.18 eV)	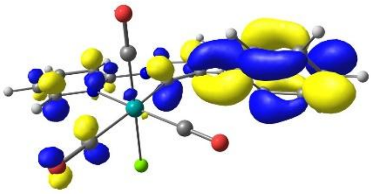
**HOMO–3 / 91**<br>(–7.37 eV)	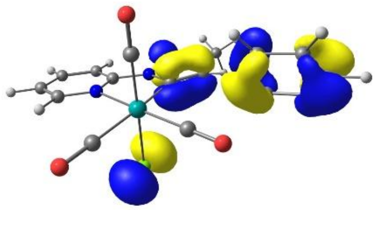		**LUMO+3 / 98**<br>(–0.75 eV)	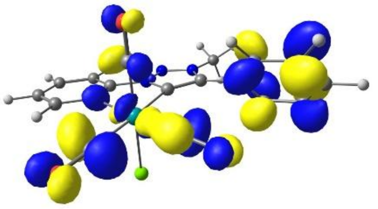
**HOMO–4 / 90**<br>(–7.72 eV)	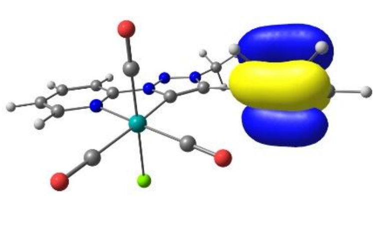		**LUMO+4 / 99**<br>(–0.64 eV)	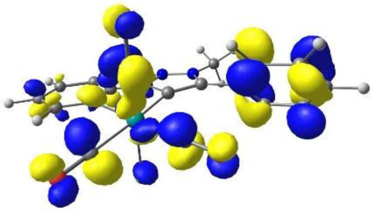
**HOMO–5 / 89**<br>(–7.91 eV)	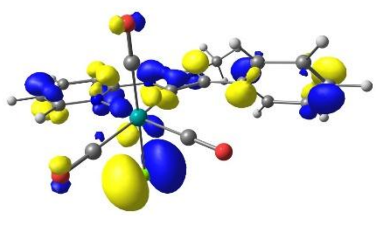		**LUMO+5 / 100**<br>(–0.41 eV)	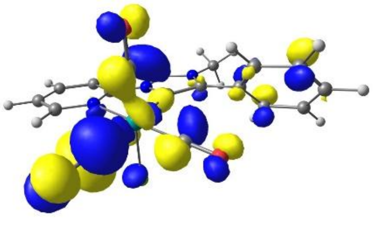

Figure S37. The isodensity plots of the frontier molecular orbitals of **Re-T-Tapy-Me** (in dichloromethane).

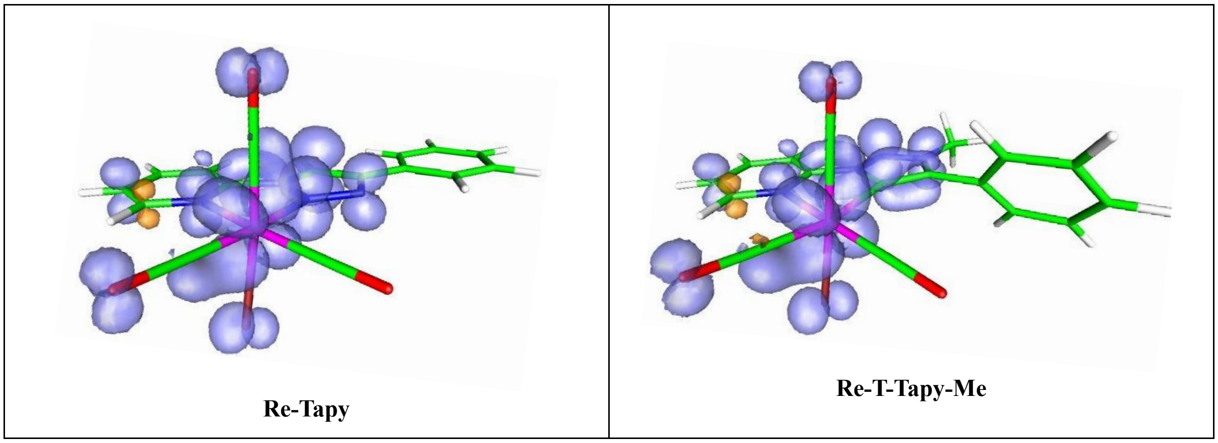

Figure S38. Spin density distribution for the lowest triplet state (T_1) of **Re-Tapy** and **Re-T-Tapy-Me** (in dichloromethane) calculated based on the optimized triplet state with DFT method at the PBE1PBE/LanL2DZ level (isovalue surface of 0.0045 and –0.0035 au).

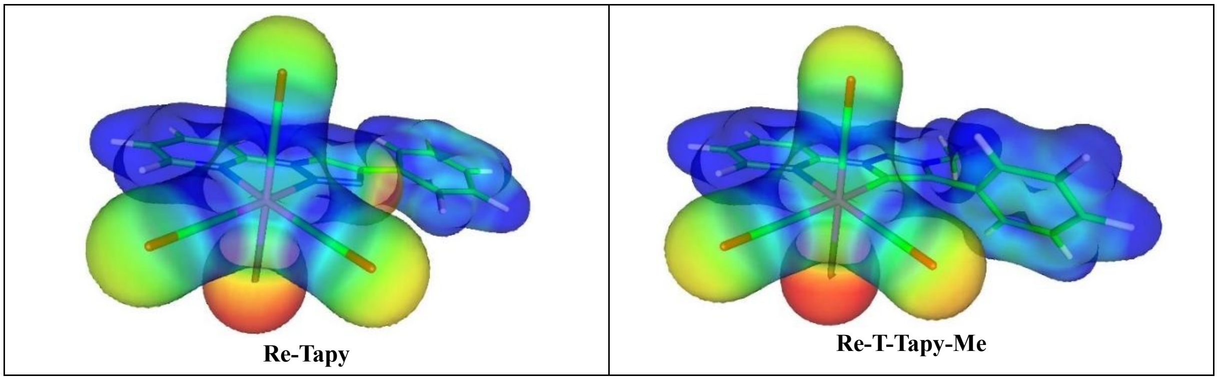

Figure S39. Molecular Electrostatic Potential (MEP) of **Re-Tapy** and **Re-T-Tapy-Me** (in dichloromethane) on the $\rho(r) = 0.02$ au isodensity surface, calculated based on the optimized ground state geometry with DFT method at the PBE1PBE/LanL2DZ level. Mapping colours range from red –0.08 au to blue +0.08 au.

MEP surface plot helps to understand visually the relative polarity of the molecule, as shown in Figure 9. It is also useful to explain quantitatively hydrogen bonding, reactivity and structure–activity relationship of molecules including the biomolecules and drugs. MEP helps to find the sites for electrophilic and nucleophilic attacks as well as hydrogen bonding interactions. The MEP surfaces of **Re-Tapy** and **Re-T-Tapy-Me** studied by PBE1PBE/LanL2DZ were generated by mapping electrostatic potential onto the molecular electron density surface. In the MEP surface map, regions are represented by different colors which corresponds to different values of the electrostatic potential. The maximum negative region which preferred site for electrophilic attack is indicated as red color, whereas the maximum positive region which preferred site for nucleophilic attack is indicated as blue color. Potential increases in the order red < orange < yellow < green < cyan < blue, where red shows the strongest repulsion and blue shows the strongest attraction. Regions having the negative potential are over the electronegative atoms while the regions having the positive potential are over the electropositive atoms.

Negative electrostatic potential regions (red colour) of complexes **Re-Tapy** and **Re-T-Tapy-Me** are mainly localized around the chlorine Cl, the nitrogens N of the ligand as well as carbonyl oxygens. The positive electrostatic potential regions (blue colour) are around the hydrogen atoms.

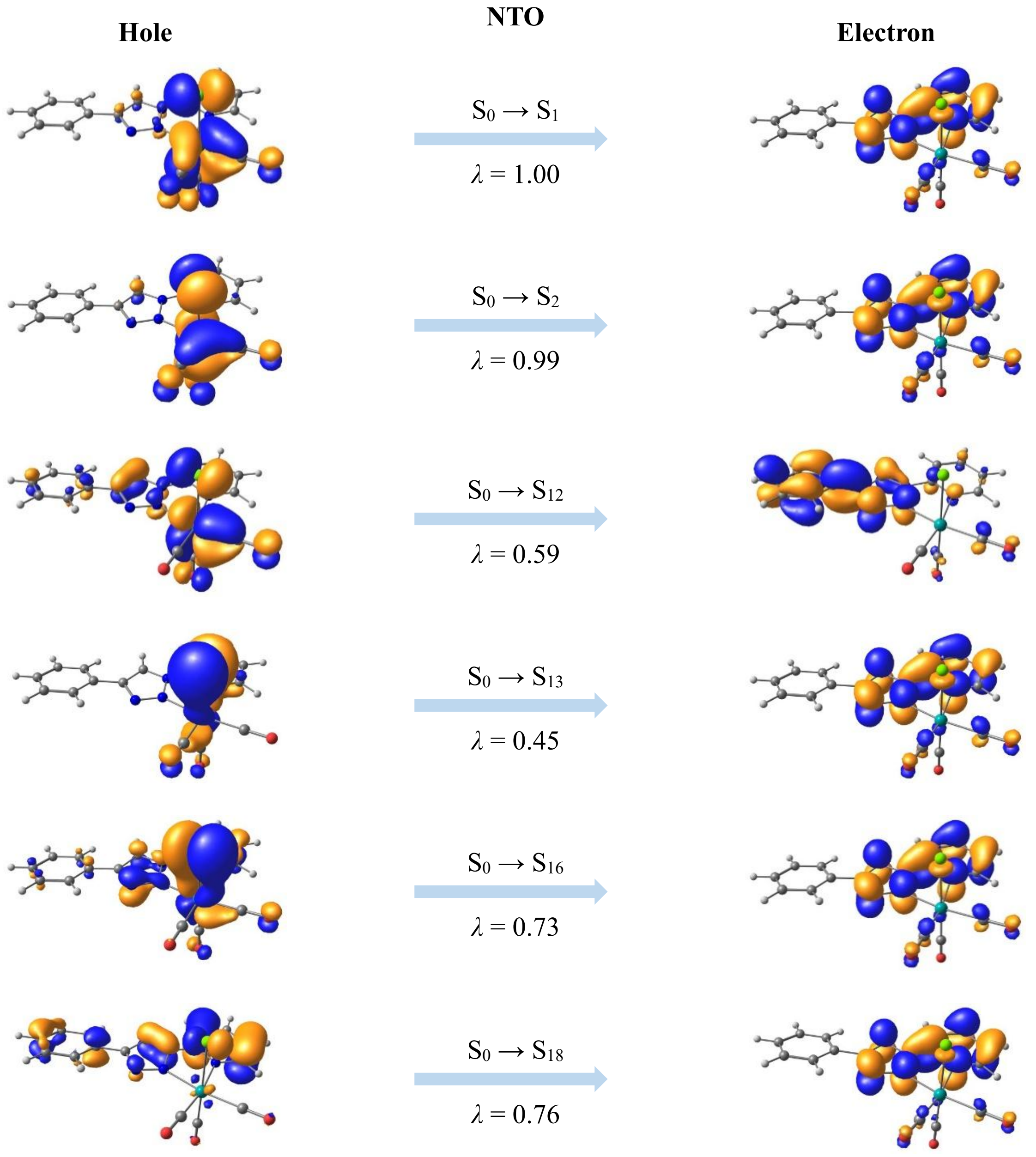

Figure S40. The natural transition orbitals (NTOs) of transitions $S_0 \rightarrow S_1$, $S_0 \rightarrow S_2$, $S_0 \rightarrow S_{12}$, $S_0 \rightarrow S_{13}$, $S_0 \rightarrow S_{16}$ and $S_0 \rightarrow S_{18}$ in **Re-Tapy**; λ is the fraction of the hole–particle contribution to the excitation.

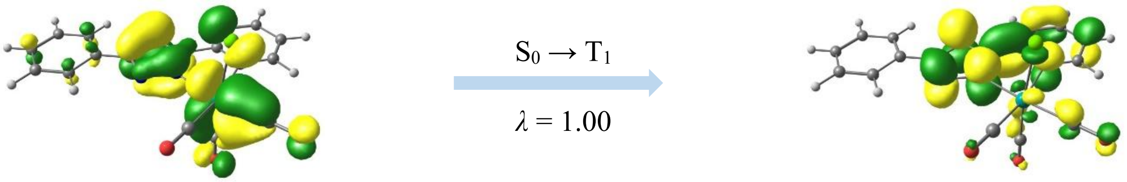

Figure S41. The natural transition orbitals (NTOs) of transition $S_0 \rightarrow T_1$ in **Re-Tapy**; λ is the fraction of the hole–particle contribution to the excitation.

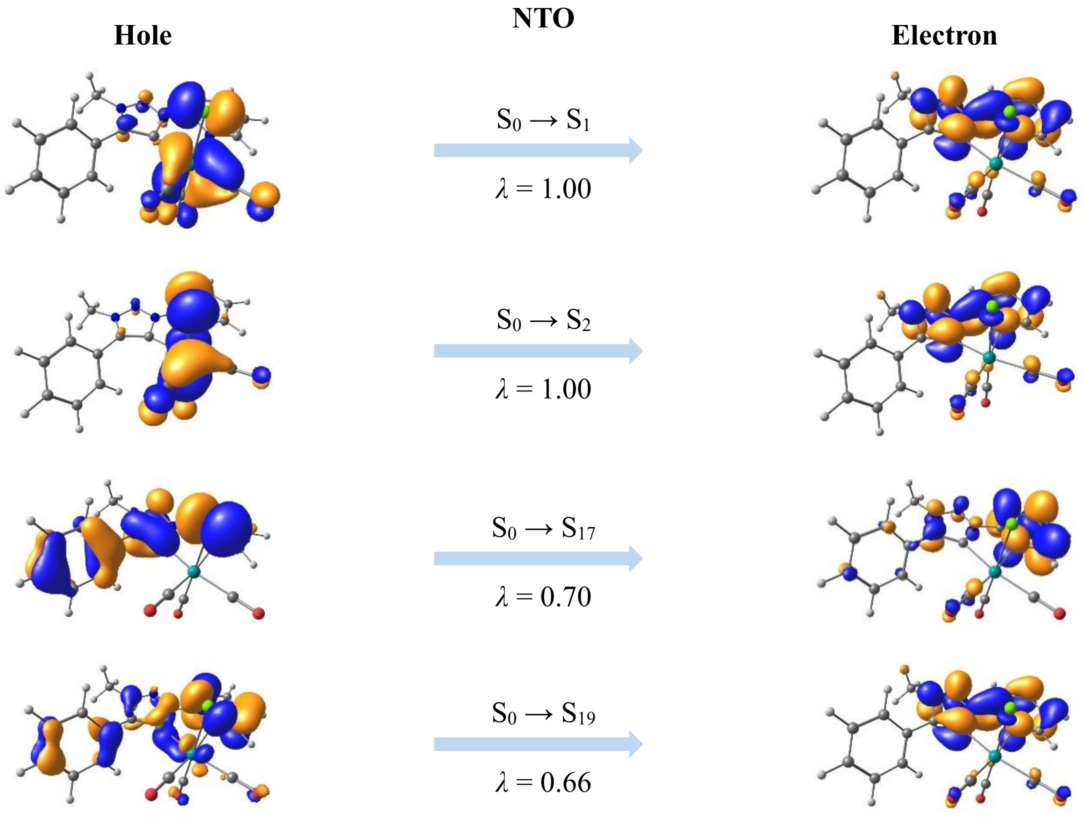

Figure S42. The natural transition orbitals (NTOs) of transitions $S_0 \rightarrow S_1$, $S_0 \rightarrow S_2$, $S_0 \rightarrow S_{17}$ and $S_0 \rightarrow S_{19}$ in **Re-T-Tapy-Me**; λ is the fraction of the hole–particle contribution to the excitation.

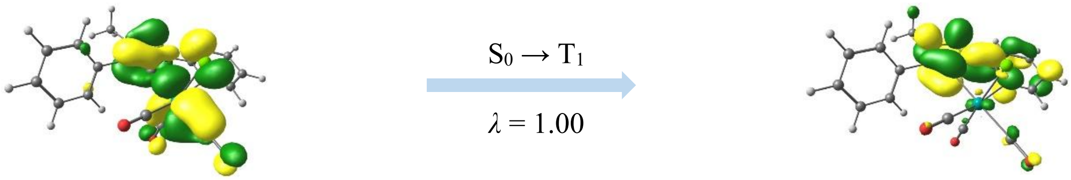

Figure S43. The natural transition orbitals (NTOs) of transition $S_0 \rightarrow T_1$ in **Re-T-Tapy-Me**; λ is the fraction of the hole–particle contribution to the excitation.

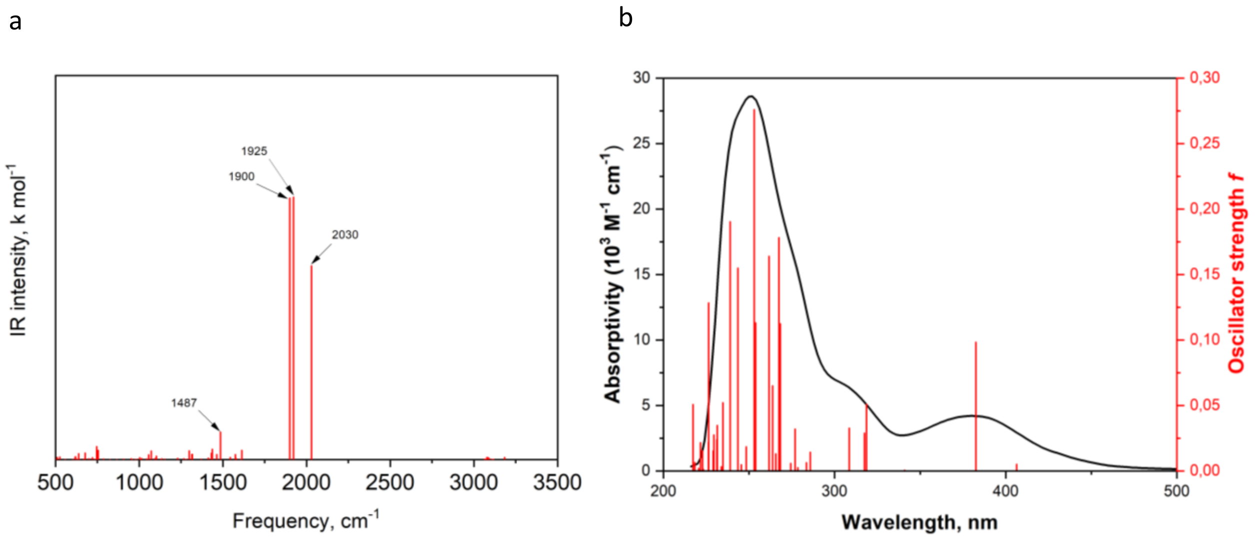

Figure S44. The experimental (black) and simulated (red) FT-IR spectra (a) and UV-Vis absorption spectra (b) of **Re-Tapy** (in dichloromethane).

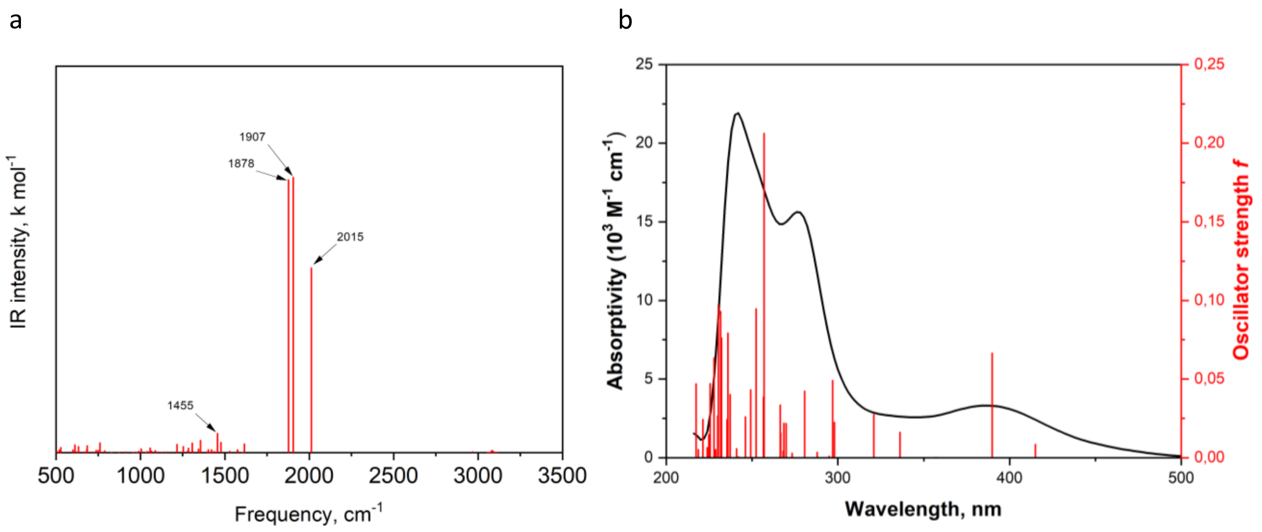

Figure S45. The experimental (black) and simulated (red) FT-IR spectra (a) and UV-Vis absorption spectra (b) of **Re-T-Tapy-Me** (in dichloromethane).

Spectroscopy

a) Re-Tapy (λ_{em} = 620 nm)

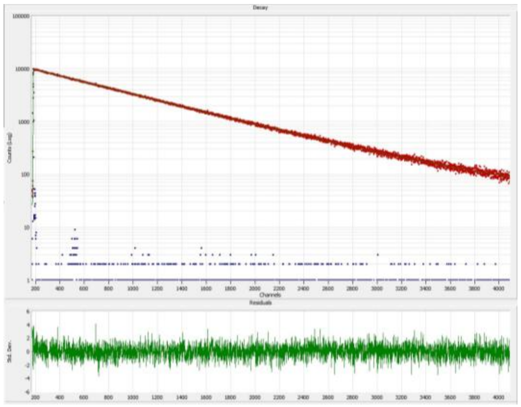

SHIFT = 0.503383 ch
2.093341E-10 sec
S.Dev = 5.632416E-12 sec

T1 = 736.6143 ch
3.063243E-07 sec
S.Dev = 1.968081E-10 sec

A = 26.52323
S.Dev = 0.3351659

B1 = 0.2604217
[100.00 Rel.Ampl]
S.Dev = 1.066516E-04

CHISQ = 1.03644
[3913 degrees of freedom]

Chi-squared Probability = 5.48033%
Durbin-Watson Parameter = 1.980603

b) Re-T-Tapy-Me (λ_{em} = 600 nm)

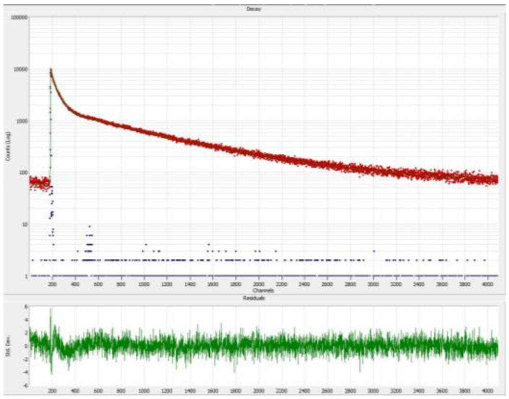

SHIFT = 9.087509E-03 ch
3.779081E-12 sec
S.Dev = 5.000592E-12 sec

T1 = 60.41254 ch
2.512282E-08 sec
S.Dev = 1.372031E-10 sec
T2 = 758.4584 ch
3.154083E-07 sec
S.Dev = 7.388005E-10 sec
T3 = 11.69857 ch
4.864904E-09 sec
S.Dev = 1.472847E-10 sec

A = 57.3094
S.Dev = 0.2263212

B1 = 0.1586759
[22.42 Rel.Ampl]
S.Dev = 5.065177E-04
B2 = 4.231613E-02
[75.07 Rel.Ampl]
S.Dev = 5.385738E-05
B3 = 9.180132E-02
[2.51 Rel.Ampl]
S.Dev = 1.452228E-03

CHISQ = 1.10868
[4078 degrees of freedom]

Chi-squared Probability = 1.0297E-04%
Durbin-Watson Parameter = 1.850376

c) Re-T-Tapy-Et (λ_{em} = 632 nm)

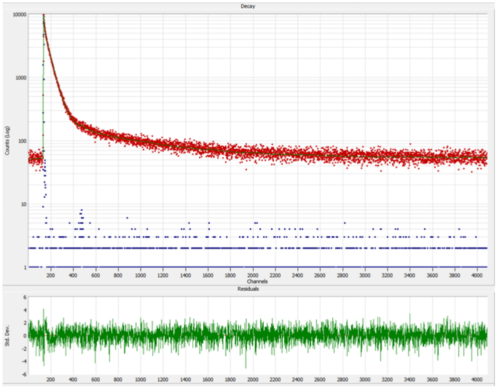

SHIFT = 0.1477196 ch
6.142986E-11 sec
S.Dev = 7.0428E-12 sec

T1 = 55.32145 ch
2.300567E-08 sec
S.Dev = 8.633903E-11 sec
T2 = 655.7765 ch
2.727076E-07 sec
S.Dev = 3.536494E-09 sec
T3 = 4.944054 ch
2.056007E-09 sec
S.Dev = 6.753221E-11 sec

A = 49.5909
S.Dev = 0.1577041

B1 = 0.1640381
[70.41 Rel.Ampl]
S.Dev = 3.545575E-04
B2 = 4.248792E-03
[21.62 Rel.Ampl]
S.Dev = 2.722986E-05
B3 = 0.2078022
[7.97 Rel.Ampl]
S.Dev = 2.132139E-03

CHISQ = 1.1185
[4078 degrees of freedom]

Chi-squared Probability = 1.2324E-05%
Durbin-Watson Parameter = 1.862169

d) Re-T-Pyta$_{(1,2,3)}$-Et (λ_{em} = 580 nm)

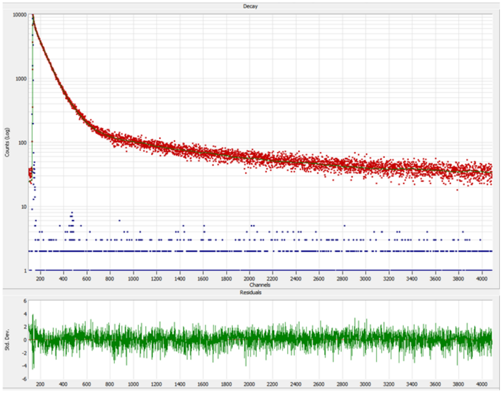

SHIFT = 0.3425743 ch
1.424611E-10 sec
S.Dev = 6.396288E-12 sec

T1 = 8.608401 ch
3.579842E-09 sec
S.Dev = 1.101378E-10 sec
T2 = 99.35941 ch
4.131905E-08 sec
S.Dev = 1.037177E-10 sec
T3 = 944.6835 ch
3.928508E-07 sec
S.Dev = 5.342656E-09 sec

A = 24.14287
S.Dev = 0.1635802

B1 = 0.132036
[4.78 Rel.Ampl]
S.Dev = 1.488522E-03
B2 = 0.1856836
[77.62 Rel.Ampl]
S.Dev = 2.767447E-04
B3 = 4.426842E-03
[17.59 Rel.Ampl]
S.Dev = 2.518684E-05

CHISQ = 1.097658
[3979 degrees of freedom]

Chi-squared Probability = 1.1758E-03%
Durbin-Watson Parameter = 1.863274

Figure S46. Emission decays of (a) **Re-Tapy,** (b) **Re-T-Tapy-Me**, (c) **Re-T-Tapy-Et** and (d) **Re-T-Pyta-Et** (~ 2 × 10^{-5} M) in aerated dichloromethane solutions. λ_{ex} = 371 nm. Emission wavelength indicated on the spectra.

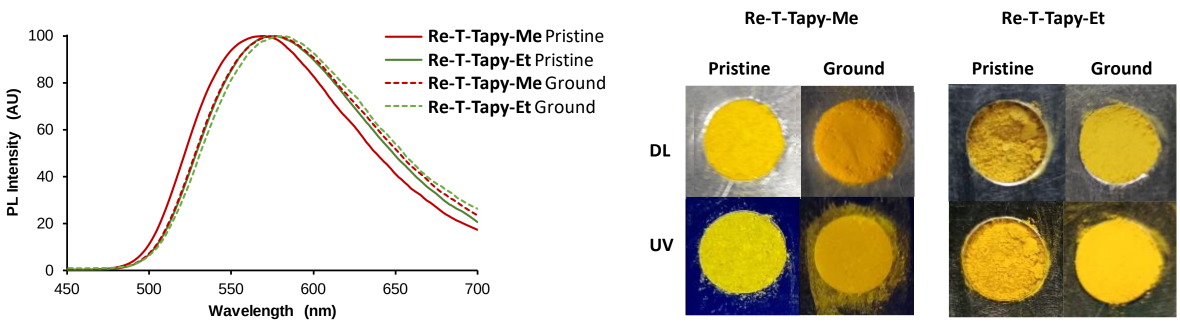

Figure S47. Right: Normalized emission spectra of **Re-T-Tapy-Me** and **Re-T-Tapy-Et** as pristine powders (solid lines) and ground powders (dotted line) (λ_{ex} = 380 nm). Left: Images of the corresponding samples in the daylight and under UV illumination.

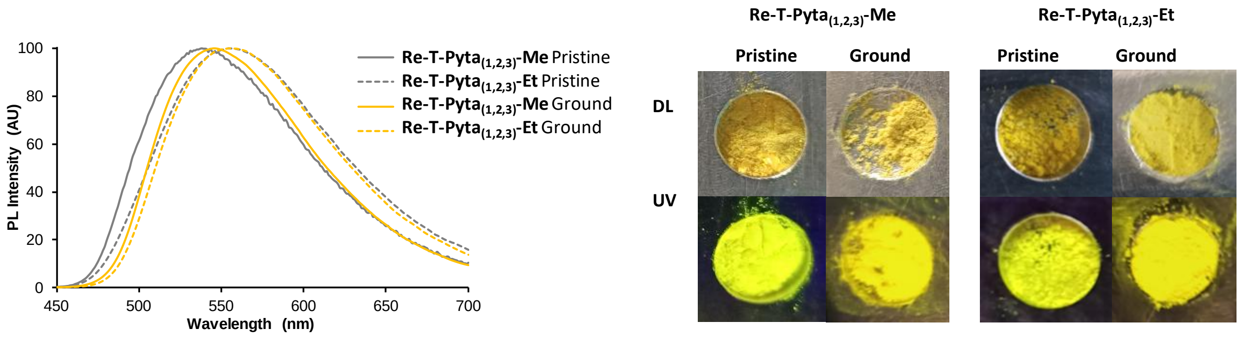

Figure S48. Right: Normalized emission spectra of **Re-T-Pyta$_{(1,2,3)}$-Me** and **Re-T-Pyta$_{(1,2,3)}$-Et** as pristine powders (solid lines) and ground powders (dotted line) (λ_{ex} = 380 nm). Left: Images of the corresponding samples in the daylight and under UV illumination.

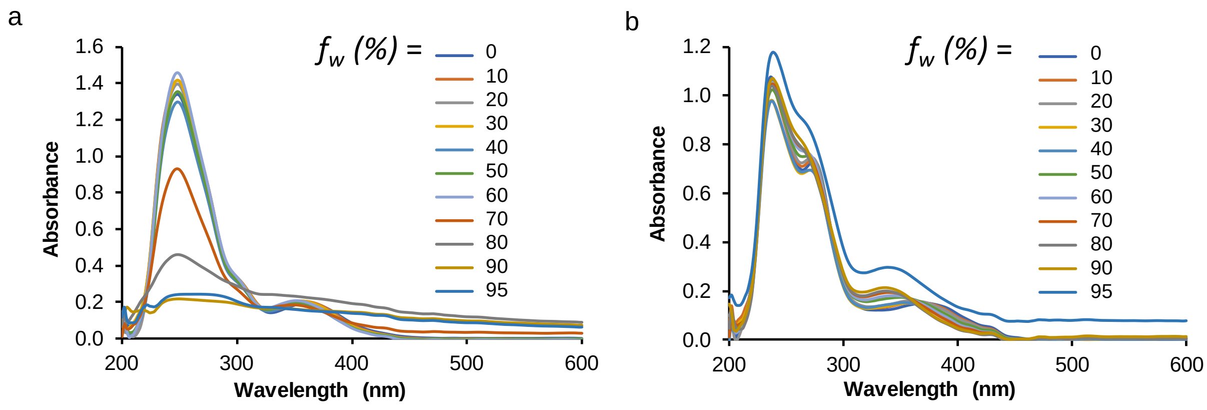

Figure S49. Absorption spectra of (a) **Re-Tapy** (5 × 10^{-5} M) and (b) **Re-T-Tapy-Me** (5 × 10^{-5} M) in acetonitrile solutions containing from 0 to 95% water.

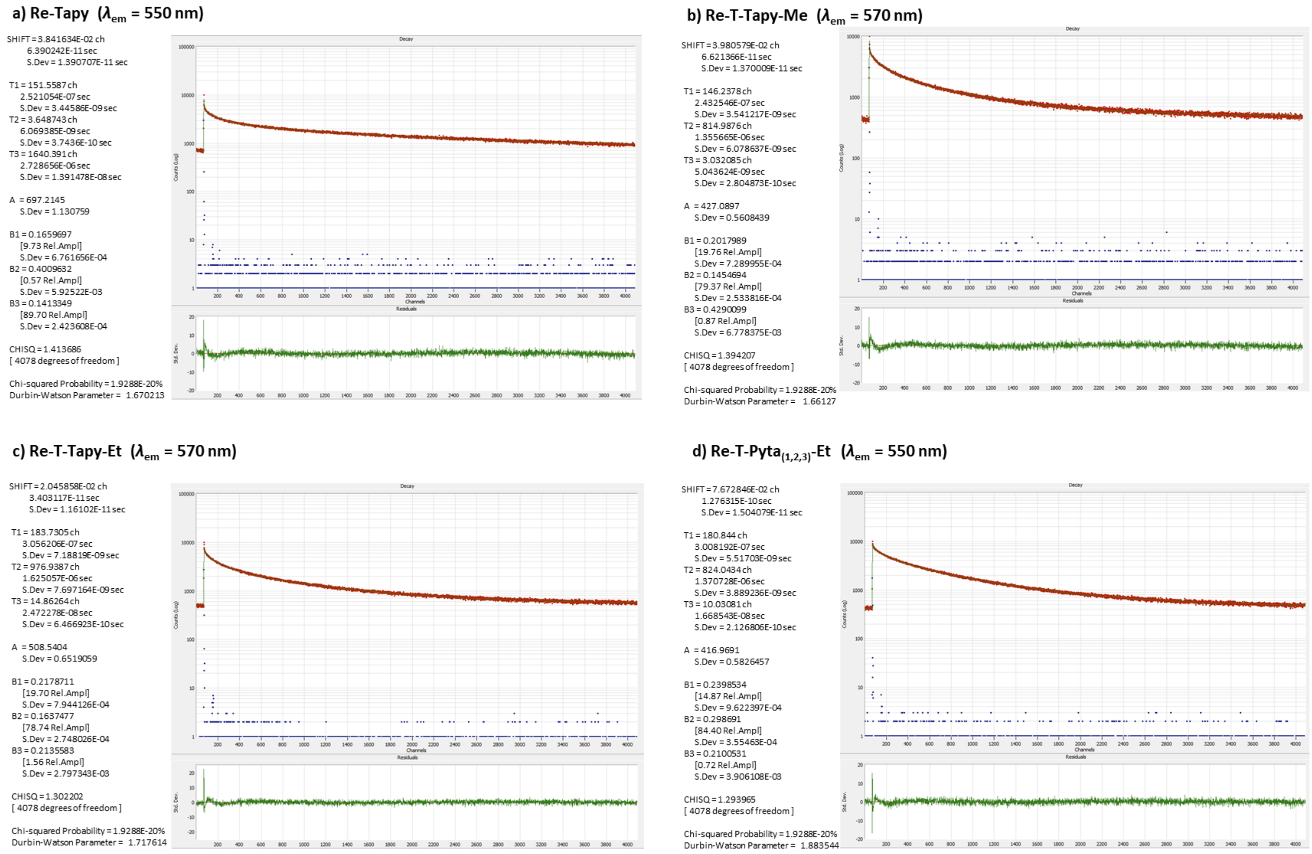

Figure S50. Emission decays of (a) **Re-Tapy,** (b) **Re-T-Tapy-Me**, (c) **Re-T-Tapy-Et** and (d) **Re-T-Pyta$_{(1,2,3)}$-Et** in the microcrystalline state (pristine powders). λ_{ex} = 371 nm. Emission wavelength indicated on the spectra.

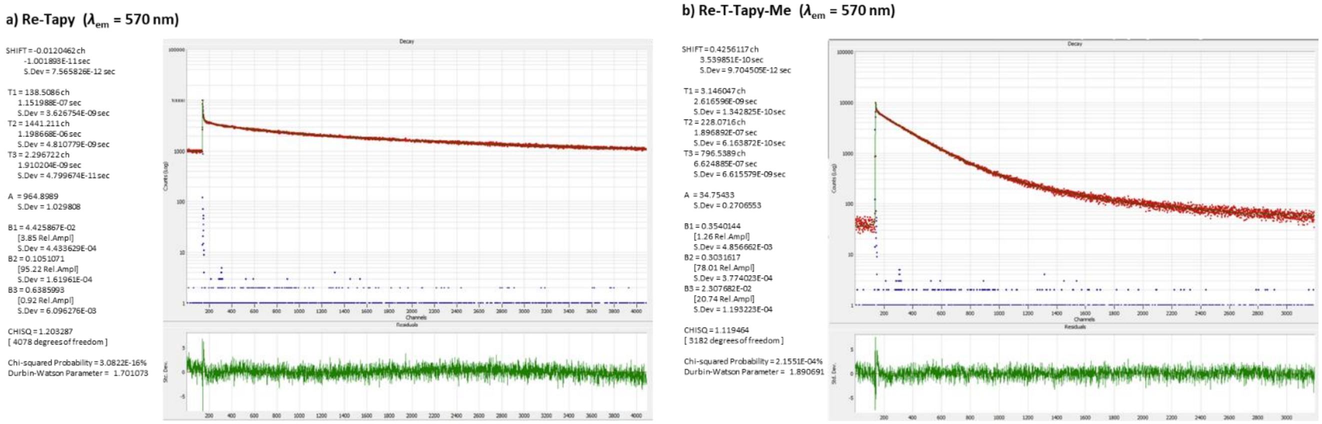

Figure S51. Emission decays of (a) **Re-Tapy** and (b) **Re-T-Tapy-Me** suspensions in water/acetonitrile 80:20 v/v. Concentration: 5×10^{-5} M. λ_{ex} = 371 nm. Emission wavelength indicated on the spectra.

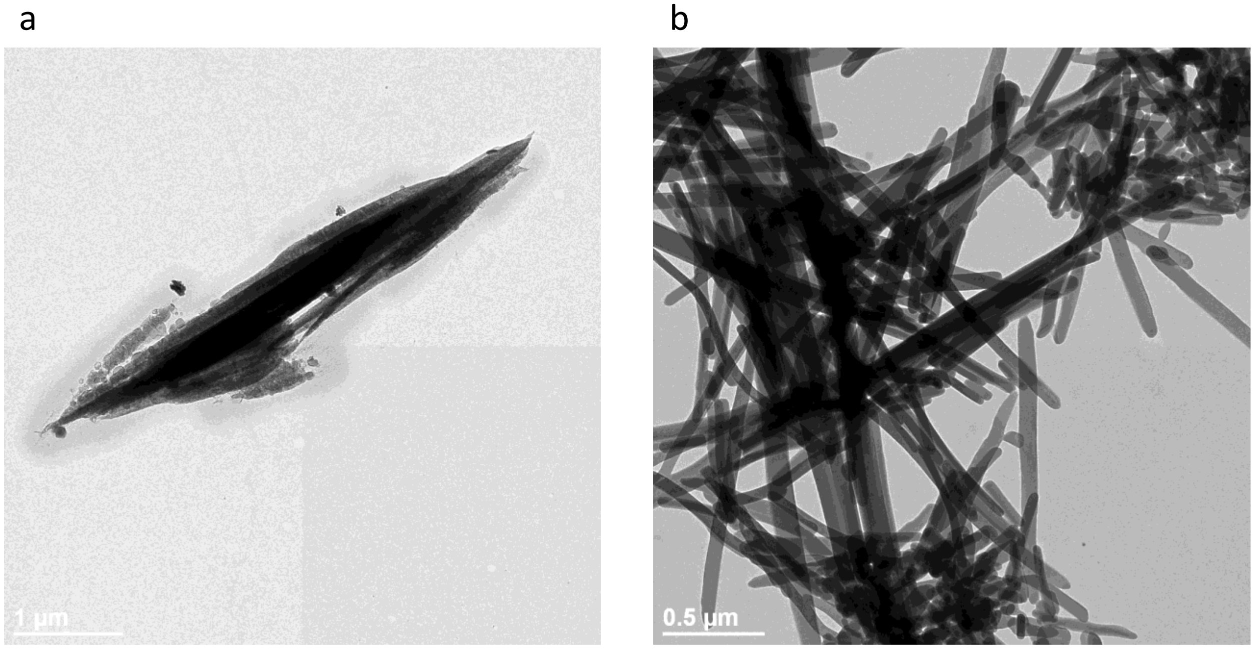

Figure S52. TEM images of the suspension of **Re-Tapy** (5×10^{-5} M) in water/acetonitrile 80:20 v/v.

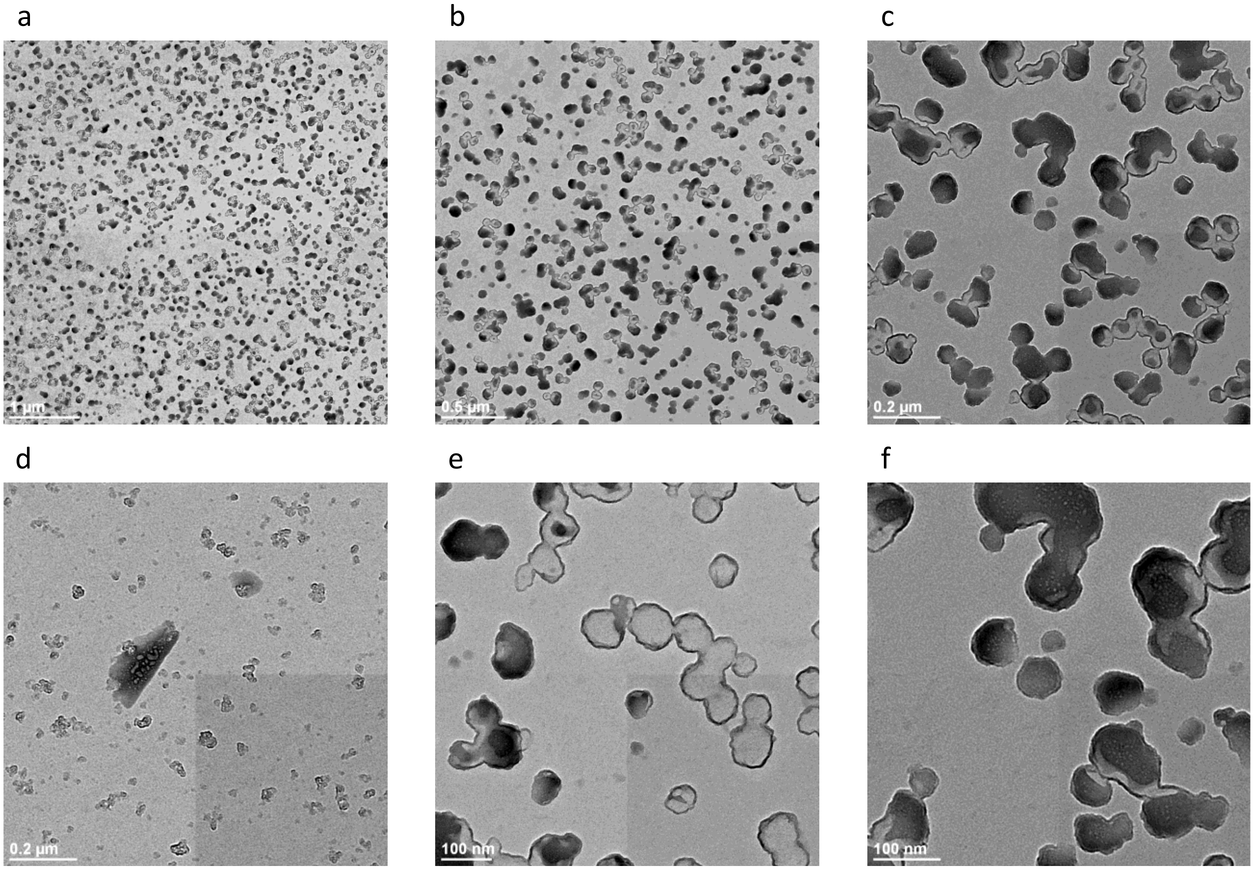

Figure S53. TEM images of the suspension of **Re-Tapy** (5×10^{-5} M) in water/acetonitrile 95:5 v/v.

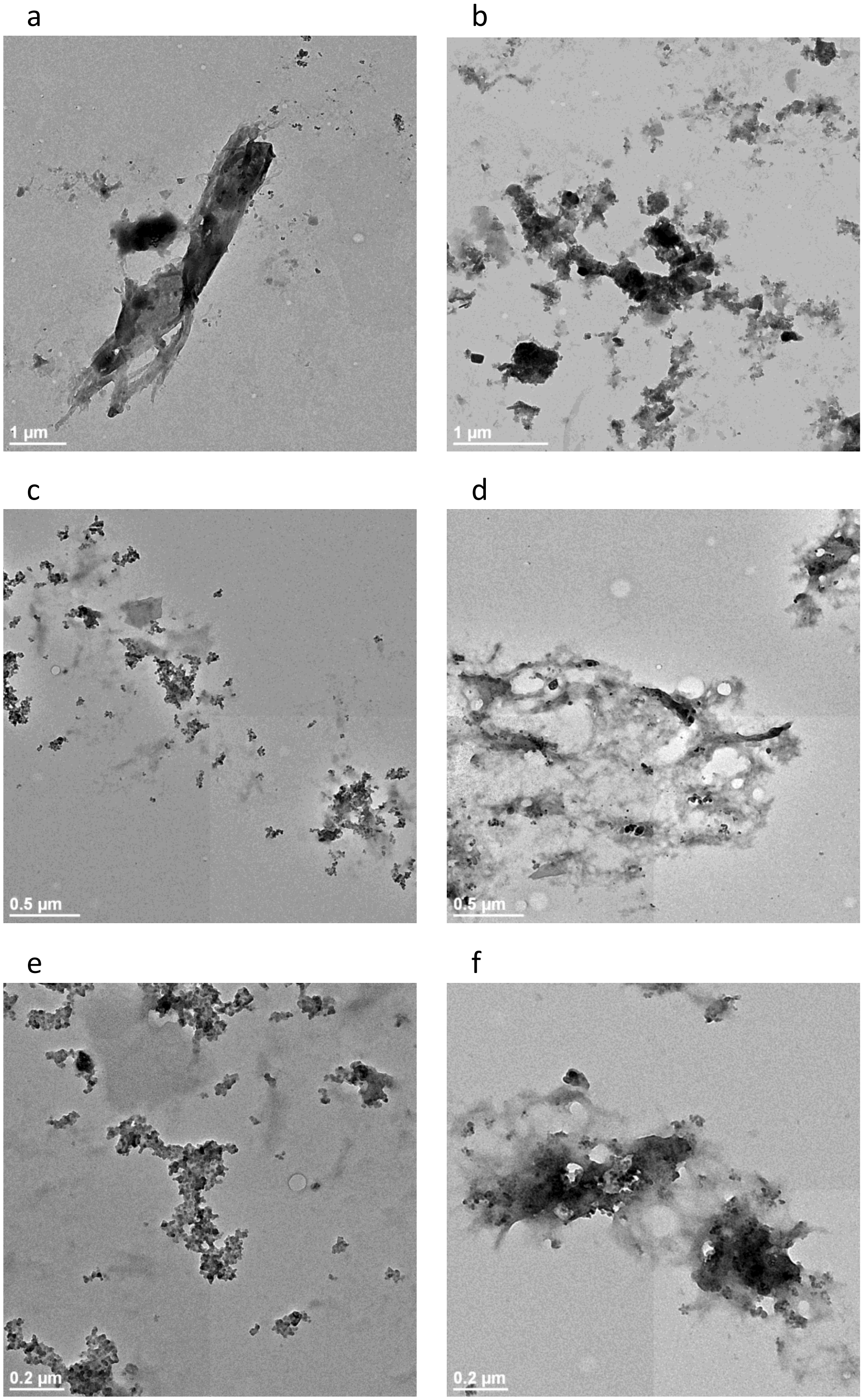

Figure S54. TEM images of the suspension of **Re-T-Tapy-Me** (5×10^{-5} M) in water/acetonitrile 80:20 v/v.

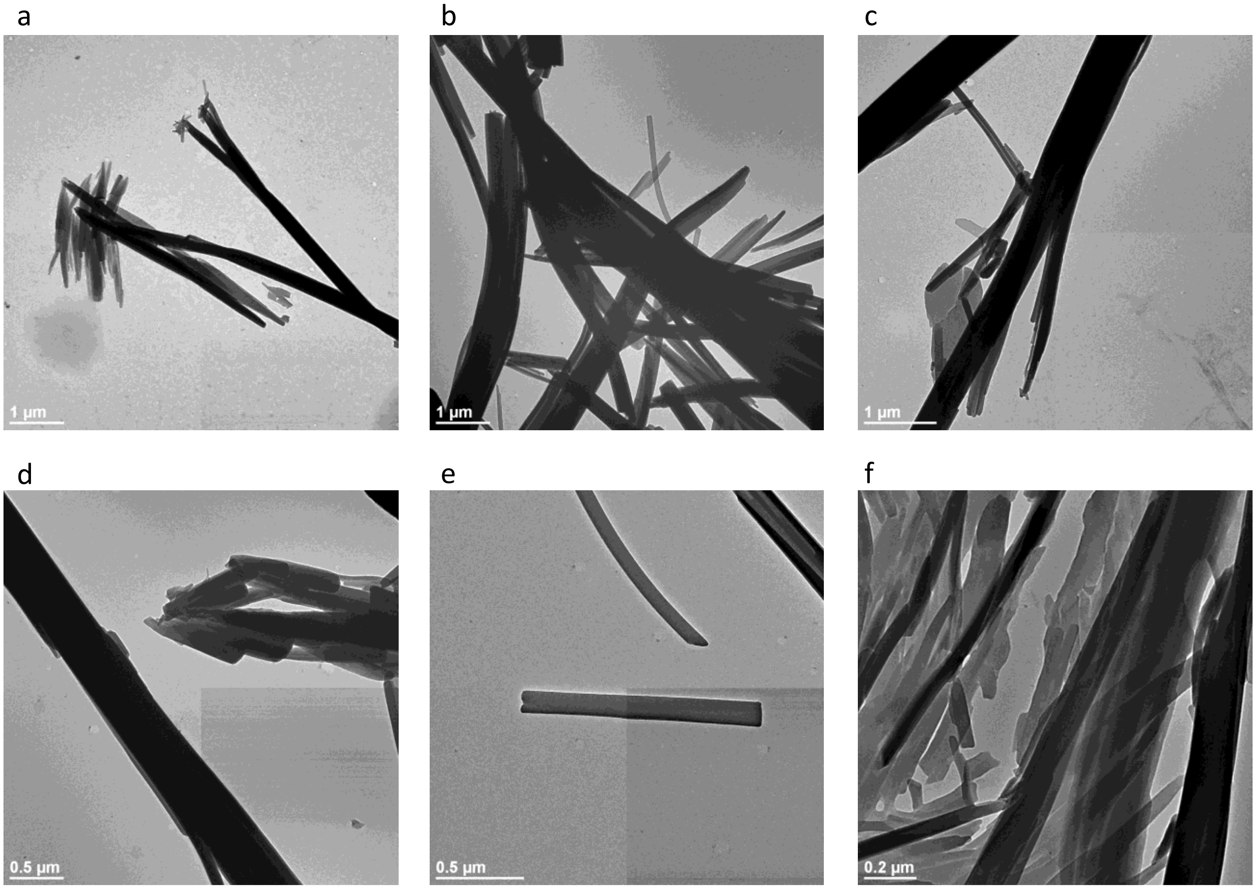

Figure S55. TEM images of the suspension of **Re-T-Tapy-Me** (5×10^{-5} M) in water/acetonitrile 95:5 v/v.

Table S17. Dynamic light scattering (DLS) results for suspensions of complexes **Re-Tapy** and **Re-T-Tapy** at 5×10^{-5} M in two different water/acetonitrile mixtures, (a) without filtration and (b) after filtration on 0.45 µm PTFE hydrophilic microfilters. f_w = water fraction; Z-average: Intensity-weighted mean hydrodynamic size; PDI: polydispersity index.

a

Complex	f_w (%)	Z-AVERAGE (nm)	PDI
Re-Tapy	80	48750 ± 19450	0.52 ± 0.14
	95	514 ± 60	0.57 ± 0.15
Re-T-Tapy-Me	80	1029 ± 256	0.84 ± 0.17
	95	4644 ± 5257	0.84 ± 0.20

b

Complex	f_w (%)	Z-AVERAGE (nm)	PDI
Re-Tapy	80	147 ± 10	0.34 ± 0.05
	95	335 ± 6	0.31 ± 0.03
Re-T-Tapy-Me	80	145 ± 14	0.18 ± 0.11
	95	123 ± 7	0.55 ± 0.14

Cartesian coordinates

Table S18. Cartesian coordinates of **Re-Tapy** in S_0 (in dichloromethane).

E = –1153.6711642			
75	-1.639988000	6.578663000	3.313888000
17	-0.399105000	8.588683000	2.477871000
6	-2.600744000	5.108260000	4.036995000
8	-3.186808000	4.210645000	4.465879000
6	-2.712658000	6.496788000	1.730668000
8	-3.361853000	6.459262000	0.781422000
6	-0.402997000	5.366333000	2.501083000
8	0.356435000	4.642901000	2.030575000
7	-2.783224000	8.078753000	4.450980000
6	-3.948161000	8.619020000	4.063170000
1	-4.361751000	8.243603000	3.136461000
6	-4.594162000	9.598826000	4.793446000
1	-5.533045000	10.000792000	4.434838000
6	-4.016345000	10.045119000	5.974924000
1	-4.494524000	10.812665000	6.571917000
6	-2.813451000	9.497587000	6.390420000
1	-2.337541000	9.821887000	7.306281000
6	-2.242201000	8.521117000	5.592761000
7	-1.027997000	7.900594000	5.935270000
7	-0.536462000	6.950964000	5.123334000
7	0.565300000	6.502879000	5.609549000
6	-0.195518000	8.064086000	6.987137000
1	-0.376187000	8.782673000	7.767524000
6	0.824757000	7.161538000	6.772395000
6	2.016242000	6.882077000	7.573172000
6	2.124926000	7.355642000	8.883877000
1	1.315116000	7.924102000	9.329783000
6	3.264043000	7.091039000	9.632270000
1	3.336167000	7.462215000	10.649051000
6	4.305097000	6.347765000	9.083938000
1	5.193675000	6.140310000	9.670746000
6	4.200385000	5.870473000	7.781001000
1	5.008666000	5.291510000	7.346880000
6	3.064953000	6.136086000	7.027264000
1	2.985912000	5.769766000	6.009885000

Table S19. Cartesian coordinates of **Re-Tapy** in S_1 (in dichloromethane).

E = –1153.6585879			
75	-1.588653000	6.680384000	3.296406000
17	-0.455218000	8.566965000	2.340669000
6	-2.593884000	5.242050000	4.160074000
8	-3.188662000	4.408005000	4.658925000
6	-2.664048000	6.468901000	1.682642000
8	-3.285131000	6.326625000	0.734127000
6	-0.374149000	5.423465000	2.431878000
8	0.336354000	4.682919000	1.932686000
7	-2.754588000	8.111219000	4.405584000
6	-3.930571000	8.662240000	4.029750000
1	-4.314575000	8.337200000	3.070466000
6	-4.612969000	9.576903000	4.787262000
1	-5.548302000	9.982393000	4.424888000
6	-4.068637000	9.969074000	6.034574000
1	-4.584566000	10.688062000	6.659939000
6	-2.878789000	9.430113000	6.447664000
1	-2.436537000	9.708933000	7.396006000
6	-2.233221000	8.500371000	5.617113000
7	-1.042806000	7.917455000	5.939172000
7	-0.490502000	7.025146000	5.062172000
7	0.645045000	6.565533000	5.551698000
6	-0.206805000	8.029093000	7.011722000
1	-0.410441000	8.689058000	7.835644000
6	0.844834000	7.162768000	6.738939000
6	2.028589000	6.881120000	7.553340000
6	2.171037000	7.434679000	8.829601000
1	1.398253000	8.080120000	9.234173000
6	3.297615000	7.161378000	9.594985000
1	3.393424000	7.597977000	10.583653000
6	4.297151000	6.330359000	9.098550000
1	5.176362000	6.116898000	9.697370000
6	4.162032000	5.774794000	7.829014000
1	4.937508000	5.126583000	7.434106000
6	3.038379000	6.047718000	7.060875000
1	2.933423000	5.618122000	6.070987000

Table S20. Cartesian coordinates of **Re-Tapy** in T_1 (in dichloromethane).

E = –1153.5808207			
75	-1.560588000	6.691891000	3.327497000
17	-0.663446000	8.663450000	2.170916000
6	-2.420409000	5.142088000	4.116070000
8	-2.928671000	4.220191000	4.562932000
6	-2.744081000	6.459578000	1.754656000
8	-3.433044000	6.316809000	0.857416000
6	-0.238861000	5.553594000	2.510505000
8	0.569241000	4.887841000	2.045827000
7	-2.765747000	8.128138000	4.428211000
6	-3.935094000	8.673364000	4.044545000
1	-4.317787000	8.343642000	3.086605000
6	-4.622841000	9.593280000	4.800737000
1	-5.558889000	9.996289000	4.437111000
6	-4.080797000	9.986533000	6.037928000
1	-4.598168000	10.707435000	6.660870000
6	-2.887556000	9.454729000	6.457233000
1	-2.444786000	9.740621000	7.402840000
6	-2.243738000	8.523522000	5.623968000
7	-1.053990000	7.947639000	5.935213000
7	-0.498534000	7.045482000	5.025599000
7	0.644593000	6.558578000	5.536358000
6	-0.208838000	8.039817000	7.000638000
1	-0.393230000	8.689321000	7.837598000
6	0.846330000	7.142744000	6.707182000
6	2.014833000	6.855733000	7.530024000
6	2.196613000	7.492662000	8.763274000
1	1.467440000	8.211091000	9.121898000
6	3.312369000	7.211583000	9.540263000
1	3.442925000	7.711306000	10.494104000
6	4.258921000	6.293387000	9.096974000
1	5.130669000	6.074922000	9.704728000
6	4.084858000	5.655301000	7.869947000
1	4.821205000	4.939154000	7.520985000
6	2.972886000	5.932645000	7.090812000
1	2.833159000	5.440260000	6.135189000

Table S21. Cartesian coordinates of **Re-T-Tapy-Me** in S_0 (in dichloromethane).

E = –1192.9217412			
75	4.718312000	3.676625000	3.694436000
17	4.170356000	1.403023000	2.726176000
8	2.099297000	3.748756000	5.276469000
8	5.517028000	6.377666000	4.868361000
8	3.306107000	5.063519000	1.311675000
7	6.664827000	3.338050000	2.672993000
7	7.119316000	2.184748000	4.593594000
7	7.860682000	1.440280000	5.391107000
7	7.119211000	1.321393000	6.454484000
6	3.088444000	3.730078000	4.683626000
6	5.217445000	5.352954000	4.418931000
6	3.827134000	4.544726000	2.196900000
6	6.994647000	3.773001000	1.448503000
1	6.258883000	4.387437000	0.946319000
6	8.201909000	3.462656000	0.848059000
1	8.417896000	3.841228000	-0.142908000
6	9.110897000	2.667199000	1.534962000
1	10.064064000	2.404723000	1.090998000
6	8.786861000	2.208921000	2.802202000
1	9.455979000	1.587577000	3.382599000
6	7.556523000	2.576112000	3.315775000
6	5.902256000	2.566269000	5.090733000
6	5.921448000	1.974748000	6.349624000
6	4.924084000	2.011298000	7.423542000
6	4.450541000	3.247600000	7.870687000
1	4.836835000	4.159280000	7.427753000
6	3.497526000	3.307211000	8.878890000
1	3.137239000	4.271378000	9.221320000
6	3.008894000	2.135539000	9.448386000
1	2.264019000	2.183651000	10.235715000
6	3.472629000	0.901672000	9.003413000
1	3.086199000	-0.014716000	9.436440000
6	4.424883000	0.836991000	7.993916000
1	4.762748000	-0.129541000	7.634984000
6	7.636813000	0.556788000	7.578704000
1	7.282296000	-0.472497000	7.520777000
1	7.290592000	1.017705000	8.501912000
1	8.722946000	0.579925000	7.526734000

Table S22. Cartesian coordinates of **Re-T-Tapy-Me** in S_1 (in dichloromethane).

E = –1192.9069135			
75	4.728215000	3.593679000	3.681175000
17	4.042790000	1.494642000	2.739888000
8	2.082322000	3.790910000	5.241107000
8	5.846973000	6.143706000	5.018421000
8	3.287329000	5.337400000	1.512175000
7	6.604782000	3.266715000	2.635165000
7	7.171359000	2.194457000	4.583659000
7	7.923192000	1.461603000	5.412265000
7	7.154846000	1.364642000	6.508626000
6	3.074183000	3.719612000	4.671340000
6	5.425469000	5.208636000	4.516111000
6	3.817721000	4.678177000	2.278207000
6	6.917077000	3.657675000	1.384279000
1	6.150654000	4.216541000	0.860677000
6	8.118144000	3.378285000	0.778992000
1	8.301929000	3.719700000	-0.231214000
6	9.087230000	2.643006000	1.500994000
1	10.045454000	2.407020000	1.052338000
6	8.805476000	2.231049000	2.777334000
1	9.512235000	1.667627000	3.373178000
6	7.553730000	2.556351000	3.324884000
6	5.930055000	2.553113000	5.104505000
6	5.951488000	2.004679000	6.362689000
6	4.924229000	2.029684000	7.411528000
6	4.447176000	3.252034000	7.890578000
1	4.863119000	4.176336000	7.504162000
6	3.457789000	3.285860000	8.865983000
1	3.095790000	4.240987000	9.231689000
6	2.938552000	2.100323000	9.375307000
1	2.166947000	2.127489000	10.137590000
6	3.410713000	0.878598000	8.904508000
1	3.004267000	-0.049065000	9.293396000
6	4.397643000	0.841469000	7.927588000
1	4.748832000	-0.113480000	7.549682000
6	7.694589000	0.668629000	7.651725000
1	7.681788000	-0.411966000	7.487242000
1	7.092141000	0.909612000	8.525289000
1	8.721538000	0.998240000	7.815728000

Table S23. Cartesian coordinates of **Re-T-Tapy-Me** in T_1 (in dichloromethane).

E = –1192.8368669			
75	4.735786000	3.568558000	3.708423000
17	3.966778000	1.628906000	2.448275000
8	2.118828000	3.544583000	5.313258000
8	5.661659000	6.109418000	5.192741000
8	3.499514000	5.496768000	1.540532000
7	6.622009000	3.255656000	2.634628000
7	7.173319000	2.176046000	4.561939000
7	7.920081000	1.423643000	5.393595000
7	7.126933000	1.337115000	6.504519000
6	3.102380000	3.568468000	4.722666000
6	5.326084000	5.168445000	4.634554000
6	3.934272000	4.776495000	2.308643000
6	6.917925000	3.643075000	1.382877000
1	6.149865000	4.205266000	0.866075000
6	8.115834000	3.354697000	0.765529000
1	8.294597000	3.694811000	-0.246124000
6	9.076909000	2.617860000	1.476967000
1	10.030399000	2.375160000	1.020813000
6	8.805051000	2.202266000	2.758006000
1	9.511347000	1.631105000	3.346238000
6	7.558749000	2.536984000	3.310717000
6	5.916561000	2.541753000	5.079234000
6	5.941045000	2.001680000	6.351487000
6	4.917945000	2.044379000	7.398701000
6	4.432733000	3.273631000	7.852925000
1	4.836971000	4.193134000	7.444586000
6	3.453380000	3.318898000	8.837158000
1	3.086715000	4.278379000	9.186057000
6	2.948402000	2.139974000	9.375170000
1	2.182370000	2.176900000	10.142559000
6	3.427457000	0.911868000	8.927443000
1	3.031169000	-0.010447000	9.338607000
6	4.409595000	0.861588000	7.947574000
1	4.768149000	-0.098019000	7.589323000
6	7.686887000	0.688362000	7.663101000
1	7.753630000	-0.391863000	7.506924000
1	7.050377000	0.890565000	8.522246000
1	8.686064000	1.087869000	7.850825000
